# Increasing protein stability by engineering the n → π* interaction at the β-turn†

**DOI:** 10.1039/d0sc03060k

**Published:** 2020-07-30

**Authors:** Bhavesh Khatri, Puja Majumder, Jayashree Nagesh, Aravind Penmatsa, Jayanta Chatterjee

**Affiliations:** Molecular Biophysics Unit, Indian Institute of Science Bangalore 560012 India penmatsa@iisc.ac.in jayanta@iisc.ac.in; Solid State and Structural Chemistry Unit, Indian Institute of Science Bangalore India

## Abstract

Abundant n → π* interactions between adjacent backbone carbonyl groups, identified by statistical analysis of protein structures, are predicted to play an important role in dictating the structure of proteins. However, experimentally testing the prediction in proteins has been challenging due to the weak nature of this interaction. By amplifying the strength of the n → π* interaction *via* amino acid substitution and thioamide incorporation at a solvent exposed β-turn within the **GB1** proteins and Pin 1 WW domain, we demonstrate that an n → π* interaction increases the structural stability of proteins by restricting the *ϕ* torsion angle. Our results also suggest that amino acid side-chain identity and its rotameric conformation play an important and decisive role in dictating the strength of an n → π* interaction.

## Abstract 2

Amino acid residues adopt a right-handed α-helical conformation with increasing strength of the n → π* interaction. We also demonstrate a direct consequence of n → π* interactions on enhancing the structural stability of proteins.

## Graphical abstract

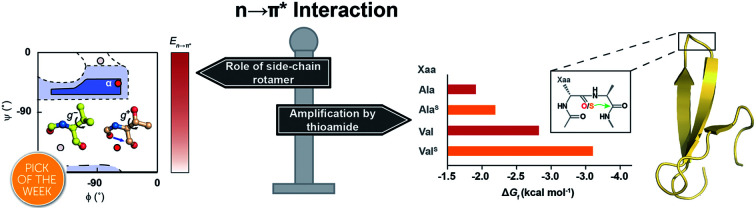

## Introduction

**Fig. 1:**
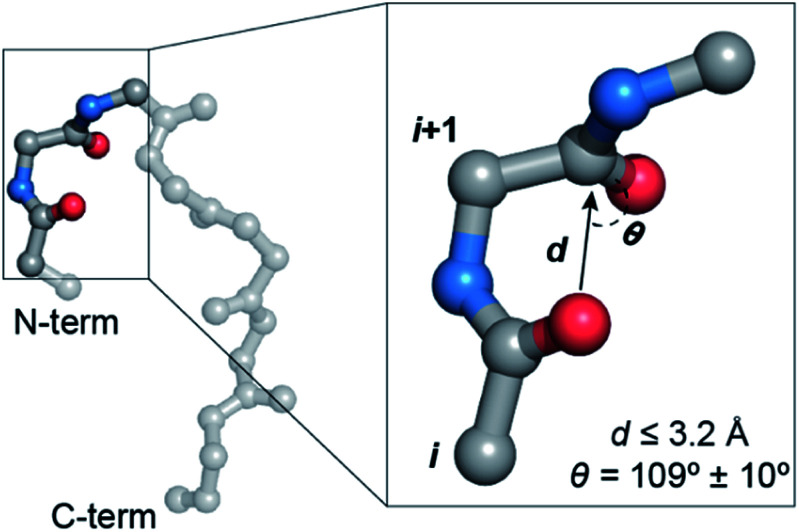
A protein backbone depicting a C=O_*i*_ → C=O_*i*+1_ n → π* interaction with the distance (*d*) and angular (*θ*) criteria used in the crystallographic analyses.

An array of noncovalent interactions including electrostatic forces, hydrogen bonds, van der Waals interactions and hydrophobic effects in a polypeptide chain dictate its three-dimensional structure and govern its folding.^1^ In particular, owing to their high abundance, the noncovalent interactions originating from the backbone (main chain) atoms of a polypeptide chain,^2^ including the classical hydrogen bonds,^3^ C–H⋯O hydrogen bonds,^4^ C5 hydrogen bonds^5^ and n → π* interactions,^6^ play a crucial role in stabilizing protein structures. The n → π* interaction originates from the donation of the lone pair (n) electron density of the carbonyl oxygen (O)_*i*_ into the empty π* orbital of the adjacent carbonyl group (C=O)_*i*+1_.^7–9^ The distance (*d* ≤ 3.2 Å) and angular criteria (*θ* = 109 ± 10°) defining an n → π* interaction are in agreement with the Bürgi–Dunitz trajectory for nucleophilic attack,^10^ which along with the associated directionality *i* → *i* + 1 (N-term → C-term) (Fig. 1) is indicative of its possible role in folding and stabilization of protein secondary structures.^11–16^

Contribution of the n → π* interaction towards the stability of the protein structure was initially reported in collagen mimetics.^17^ The enhanced thermostability of a collagen mimetic with the 4*R*-configured proline derivative compared to that with the 4*S*-configured proline derivative was attributed to the stronger n → π* interaction in the 4*R*-configured proline derivative with the *exo*-pucker of the pyrrolidine ring.^18^ The finding was exquisitely substantiated later by the high-resolution crystal structure of the oligoproline PPII helix, where the n → π* interaction was favored by the C^γ^-*exo* pucker and disfavored by the C^γ^-*endo* pucker of the pyrrolidine ring.^19^ Furthermore, the stability of this PPII helix in the absence of intramolecular hydrogen bonds and hydration emphasizes the role of the n → π* interaction in the structural stability of collagen.

For an idealized geometry, the n → π* interaction between amides contributes ∼0.3 kcal mol^−1^,^20^ which may seem moderate. However, given the ubiquity of carbonyl groups in a polypeptide chain, n → π* interactions could have a significant collective contribution towards the overall energetics of protein stability.^2^ The distribution of the n → π* interaction obtained from analyses of protein crystal structures reveals that >70% of residues in α-helices, as opposed to <5% of residues in β-sheets engage in this interaction.^11,12^ Furthermore, since one third of all amino acids in the random coil have torsion angles in the α-helical region,^21^ the n → π* interaction might have an important role in restricting the conformational ensemble of unfolded proteins.^22^ In this context, it is worth noting that random coils and turn regions of proteins show a high abundance of reciprocal n → π* interactions (back and forth donation between adjacent carbonyl pairs).^9^

The evidence of the n → π* interaction has been shown by microwave and IR spectroscopy in various small molecular systems.^23–27^ However, despite enormous excitement in this area, so far experimental measurements of the energy of an n → π* interaction in proteins and its practical consequence on protein structural stability have been lacking. Therefore, we sought to engineer an n → π* interaction at the β-turn within a protein to understand its influence on the protein structure and its stability.

**Fig. 2:**
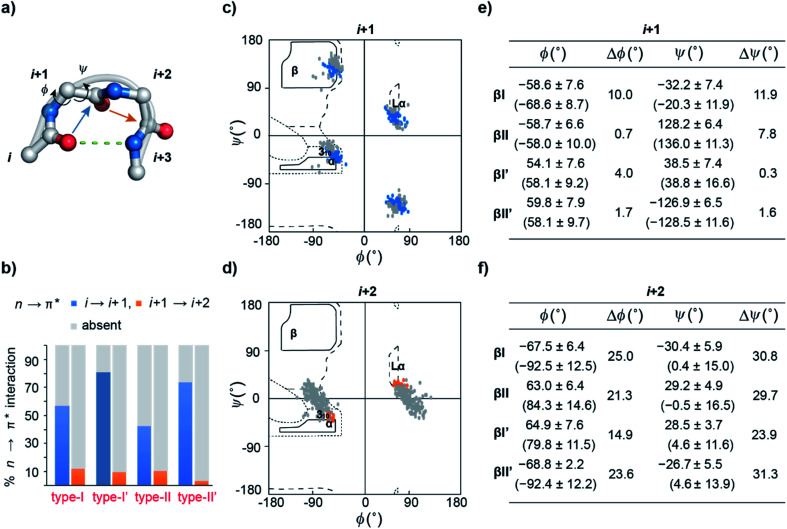
(a) A β-turn (type-II′) depicting two central residues *i* + 1 and *i* + 2 along with the C=O_*i*_⋯HN_*i*+3_ hydrogen bond, C=O_*i*_ → C=O_*i*+1_ (blue arrow) and C=O_*i*+1_ → C=O_*i*+2_ (orange arrow) n → π* interactions. (b) The abundance of C=O_*i*_ → C=O_*i*+1_ and C=O_*i*+1_ → C=O_*i*+2_ n → π* interactions in the different turns derived from PDB analyses. Ramachandran plot of (c) *i* + 1 residues engaged in the C=O_*i*_ → C=O_*i*+1_ n → π* interaction, and (d) *i* + 2 residues engaged in the C=O_*i*+1_ → C=O_*i*+2_ n → π* interaction in the β-turns. The gray dots indicate the respective turn residues, which do not engage in the n → π* interaction. Mean torsion angle ± S.D. of (e) *i* + 1 and (f) *i* + 2 residues in the presence and (absence) of the n → π* interaction. Δ*ϕ* and Δ*ψ* represent the difference between the mean torsion angles in the presence and absence of the n → π* interaction.

β-Turns (Fig. 2a) are the third most important protein secondary structure representing ∼20% of all protein residues^28^ having an important role in protein folding.^29–31^ Furthermore, substituting non-proline residues with proline residues in the β-turn leads to increased stabilization of the turn^32^ and enhanced protein stability.^33,34^ The increased stability results from the decreased backbone conformational entropy of the denatured state due to the restricted rotation of the N–C^α^ bond, also known as the *ϕ* torsion angle. Since an n → π* interaction also restricts the *ϕ* angle of an amino acid residue,^11^ we speculated that engineering an n → π* interaction at the β-turn would have direct consequence on the protein stability.

Here, by using bioinformatic analysis of the β-turn in proteins, we find an interplay between the conformational flexibility of the peptide backbone and the abundance of n → π* interactions at the two central residues, *i* + 1 and *i* + 2 of the β-turn. Through subsequent X-ray crystallography and computational analysis of synthetic **GB1** proteins with amino acid substitutions at the *i* + 2 residue of the β-turn, we show that amino acid side-chain identity and its rotameric conformation have a direct influence on the strength of an n → π* interaction. Gratifyingly, the thermal denaturation of the **GB1** proteins shows a good correlation between their stability and the strength of an n → π* interaction at the β-turn. Finally, we validate this observation in the Pin 1 WW domain, wherein by amplifying the strength of an n → π* interaction at the β-turn by thioamide incorporation, we could increase the thermal stability of the thioamidated Pin 1 WW domain.

## Results and discussion

### n → π* interaction and conformational flexibility at the β-turn

Previous computational analyses predicted that n → π* interactions confer conformational stability to the *i* + 1 residue in common type I and type II β-turns, and thus have a special role to play in the stability of turns.^11^ Therefore, we sought to examine the abundance of n → π* interactions and their possible correlation with the conformational flexibility of the peptide backbone in the β-turns.

By analyzing a non-redundant subset of high-resolution (≤2.0 Å) protein crystal structures in the Protein Data Bank (PDB), we curated 500 β-turns (identified using Promotif) representing the common type-I, type-II, type-I′, and type-II′ turns. Next, using the distance and angular criteria defining an n → π* interaction (Fig. 1), we determined the abundance of n → π* interactions at the *i* + 1 and *i* + 2 residues in the β-turns. We noted that 40–80% of the residues engage in a C=O_*i*_ → C=O_*i*+1_ n → π* interaction, whereas, only 3–12% of the residues are involved in the C=O_*i*+1_ → C=O_*i*+2_ n → π* interaction (Fig. 2b).

To identify the underlying cause of this behavior, we determined the torsion angles *ϕ* and *ψ* of *i* + 1 and *i* + 2 residues in all the β-turns and plotted them on the Ramachandran map. It was interesting to note the broader distribution of *ϕ* and *ψ* angles at the *i* + 2 residue (Fig. 2d) as opposed to the *i* + 1 residue (Fig. 2c). This is suggestive of restricted conformational freedom at the *i* + 1 residue, which is associated with the higher abundance of n → π* interactions at this site. We also calculated the difference in mean *ϕ* and *ψ* angles (Δ*ϕ* and Δ*ψ*) in the presence and absence of the n → π* interaction in the respective β-turns (Fig. 2e and f). The differences were significantly higher at the *i* + 2 residue in comparison to the *i* + 1 residue. This further indicates that the lower abundance of n → π* interactions at the *i* + 2 residue is associated with greater conformational flexibility of the peptide backbone.

### Influence of the amino acid side-chain on the n → π* interaction

**Fig. 3:**
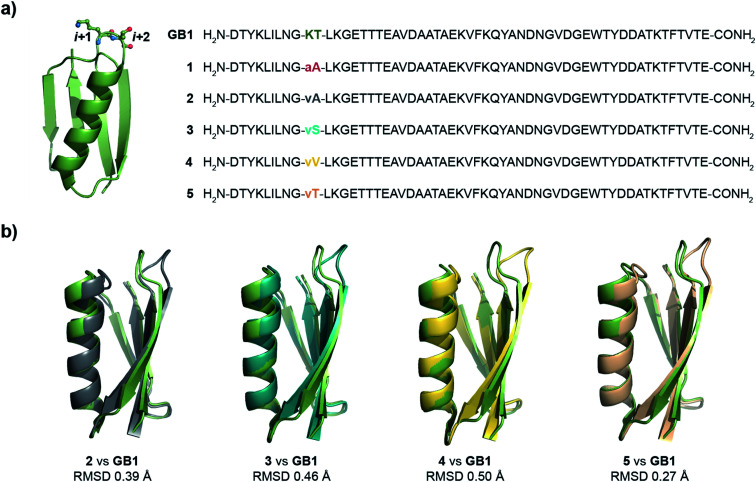
(a) Crystal structure of **GB1** with –Lys–Thr– in loop L1 forming a type-I β-turn, which has been modified to a type-II′ β-turn in **1–5**. The single letter code in lower case indicates d-amino acid. (b) The backbone overlay of **2** (6L9B), **3** (6L9D), **4** (6LJI), and **5** (6L91) with **GB1** (2QMT).

As β-turns are stabilized by the intramolecular hydrogen bond between C=O_*i*_⋯HN_*i*+3_ (Fig. 2a), the higher abundance of n → π* interactions at the *i* + 1 residue is perhaps linked with the conformational restriction of C=O_*i*_*via* hydrogen bonding. Thus, we surmised that the C=O_*i*_ → C=O_*i*+1_ n → π* interaction and the conformational space at the *i* + 1 position might be insensitive to amino acid substitution. Instead, the relatively flexible *i* + 2 residue of the β-turn (Fig. 2a), where neither the donor (n) C=O_*i*+1_ nor the acceptor (π*) C=O_*i*+2_ is constrained by the intramolecular hydrogen bond, is an ideal site to probe the role of the n → π* interaction in the protein structure and its stability. Additionally, the solvent exposure of β-turns allows for amino acid substitution and examining the influence of the amino acid side-chain on the n → π* interaction. Thus, we chose to engineer the loop L1 of the 56-residue immunoglobulin-binding domain B1 of the streptococcal protein G (**GB1**).^35–38^ The solvent exposed loop L1 in wild type **GB1** is a type-I β-turn (Fig. 3a) with lysine at the *i* + 1 position and threonine at the *i* + 2 position that lacks a C=O_*i*+1_ → C=O_*i*+2_ n → π* interaction. However, to have a better control over the turn conformation, we decided to introduce a type-II′ β-turn.^39^ Thus, we synthesized a **GB1** variant where –KT– in loop L1 was substituted with d-Ala–l-Ala (**1**) to induce a type-II′ β-turn (Fig. 3a).

**Fig. 4:**
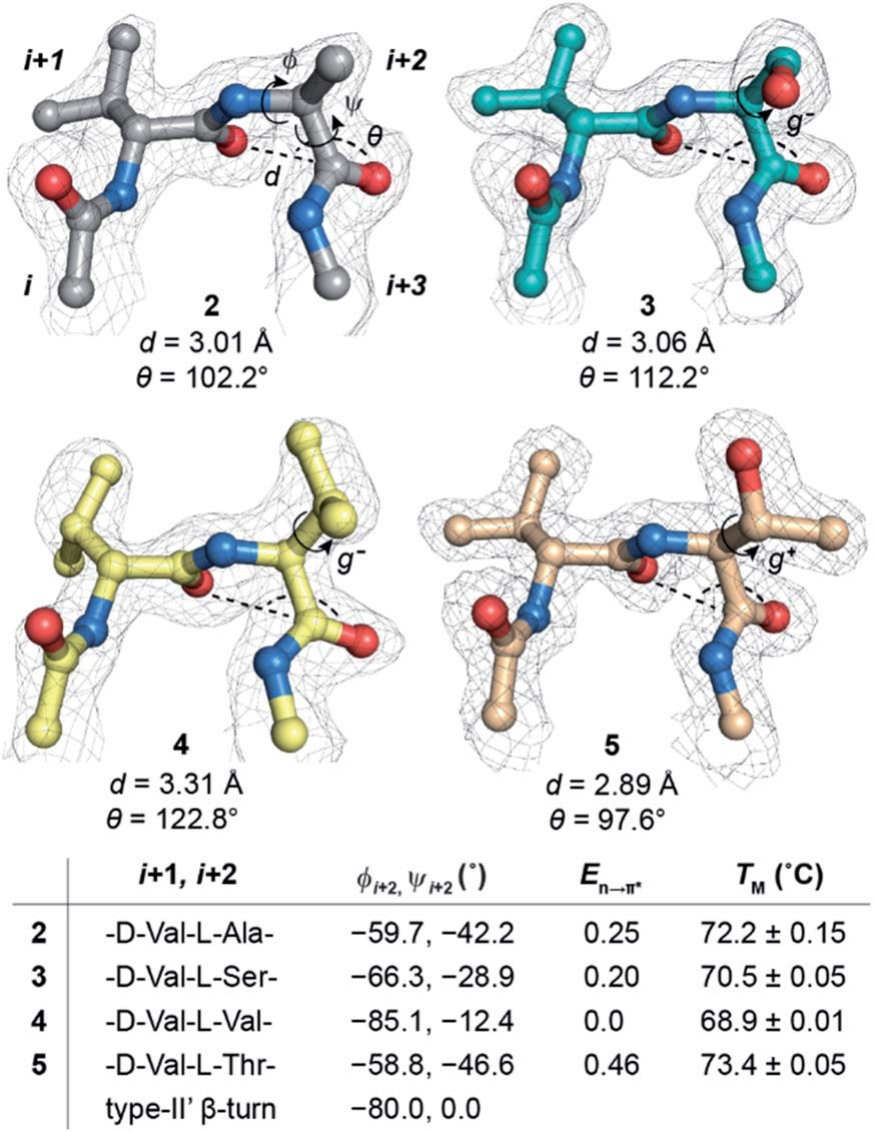
n → π* interaction at the *i* + 2 residue of the type-II′ β-turn in **GB1** variants characterized by the *d* and *θ* between C=O_*i*+1_⋯C=O_*i*+2_. The electron density map is contoured at 1.0*σ*. The *i* + 2 side-chain rotamer conformation is depicted in **3**, **4**, and **5**. Note the alteration in torsion angles of the *i* + 2 residue and its *E*_n→π*_ (kcal mol^−1^) derived from NBO analysis. The torsion angles of an ideal type-II′ β-turn are given for comparison. Also note the absence of the C=O_*i*_⋯HN_*i*+3_ intramolecular hydrogen bond. The midpoint of the thermal transition (*T*_M_ ± S.D.) of the proteins was determined by variable temperature CD.

Alanine was chosen due to its preference for an n → π* interaction^11^ and high helix propensity^40^ (preference of an amino acid to be in α-helices). Although thermal denaturation of **1** using variable temperature circular dichroism (CD) showed unfolding cooperativity similarly to **GB1** (Fig. S8 and S9†), multiple attempts to crystallize **1** remained unsuccessful. This is possibly a consequence of conformational flexibility introduced in the loop L1 by alanine substitution. Our earlier results indicated that the β-branched amino acid d-Val at the *i* + 1 site stabilizes a type-II′ β-turn more than d-Ala.^41^ Thus, we synthesized **2**, with d-Val–l-Ala in loop L1 (Fig. 3a), which readily crystallized and X-ray diffraction data were collected to a maximum resolution of 1.9 Å. The structure of **2** overlaps closely with the tertiary structure of **GB1** (backbone RMSD 0.39 Å) (Fig. 3b), although with a significant displacement of loop L1. Gratifyingly, the d-Val C=O_*i*+1_ and l-Ala C=O_*i*+2_ in the type-II′ β-turn engage in an n → π* interaction, where the torsion angles of l-Ala at the *i* + 2 site (*ϕ*, *ψ* = −59.7°, −42.2°) are remarkably close to the mean torsion angles of a right-handed α-helix (*ϕ*, *ψ* = −62°, −41°)^42^ (Fig. 4).

Encouraged by this result, we next incorporated serine with moderate helix propensity (**3**), valine (**4**) and threonine (**5**) with low helix propensity^40^ at the *i* + 2 site of the type-II′ β-turn (Fig. 3a). With the decreasing helix propensity of amino acids in the order Ala > Ser > Val, we noted an increase in both *d* and *θ* between C=O_*i*+1_ → C=O_*i*+2_, suggesting a gradual weakening of the n → π* interaction at the *i* + 2 residue (Fig. 4). Thus, to obtain a quantitative estimate of the n → π* interaction energy (*E*_n→π*_) at the *i* + 2 residue in **2**, **3**, and **4**, we resorted to NBO analysis,^43^ which clearly indicated a decreasing *E*_n→π*_ in the order **2** > **3** > **4** (Fig. 4).

**Fig. 5:**
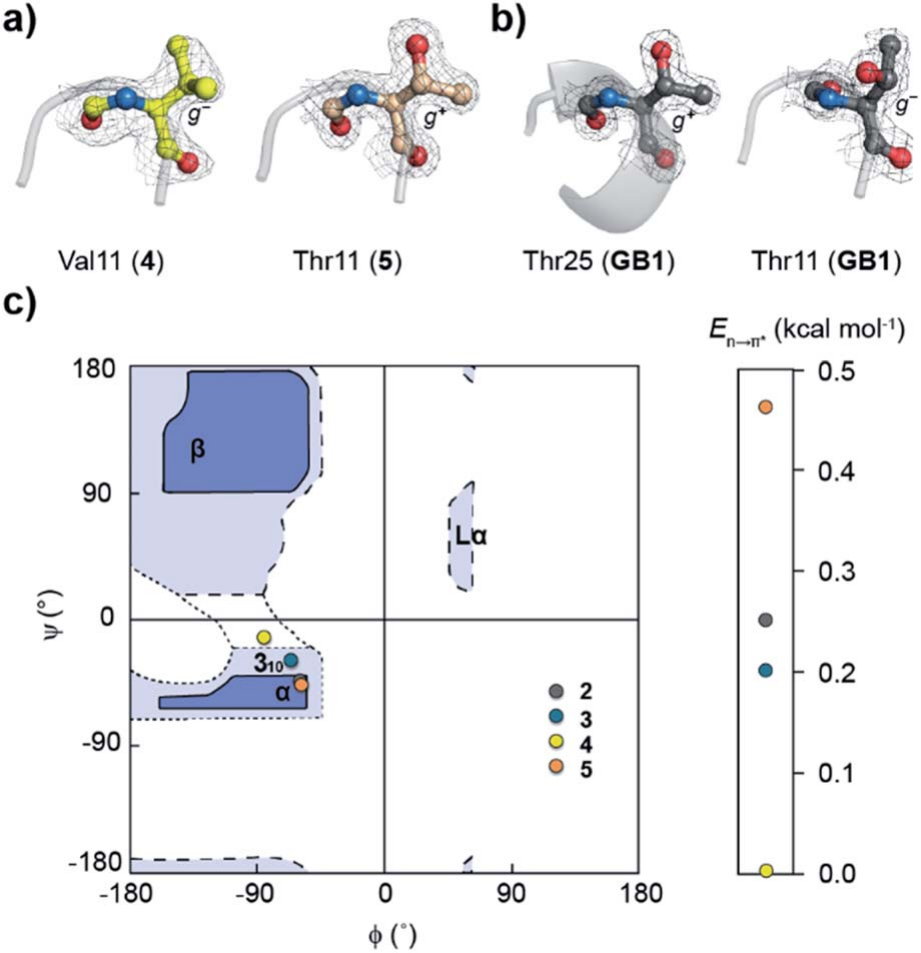
(a) Side-chain rotamer conformation of the *i* + 2 residue in the type-II′ β-turn of **4** and **5**. (b) The side-chain rotamer conformation of Thr25 (α-helix) and Thr11 (loop L1) in **GB1**, showing the presence and absence of the n → π* interaction, respectively. The electron density map is contoured at 1.0*σ*. (c) Ramachandran plot of the *i* + 2 residue at the type-II′ β-turn in **GB1** variants.

Despite the low helix propensity of threonine, we were surprised to note the shortest *d* and *θ* between C=O_*i*+1_⋯C=O_*i*+2_ at the type-II′ β-turn in **5** with an *E*_n→π*_ of 0.46 kcal mol^−1^ (Fig. 4). An overlay of the type-II′ β-turns of both the β-branched amino acids valine (**4**) and threonine (**5**) revealed a clear difference in the side-chain rotamer conformation (Fig. 5a). Valine in **4** crystallized in a *gauche*^−^ (*g*^−^) side-chain rotameric conformation, whereas threonine in **5** crystallized in a *gauche*^+^ (*g*^+^) conformation. From the statistical analyses of protein structures, it is known that β-branched amino acids favor the *g*^+^ side-chain conformation over *g*^−^ in helices.^44–47^ Thus, Thr25 in the α-helix of **GB1** with a *g*^+^ conformation engages in an n → π* interaction, whereas Thr11 at the *i* + 2 residue in loop L1 with a *g*^−^ conformation lacks the n → π* interaction (Fig. 5b). Moreover, our dataset revealed that Thr with a *g*^−^ conformation at the *i* + 2 residue in the β-turns does not engage in an n → π* interaction (Table S4†). Therefore, despite the low helix propensity, the n → π* interaction in threonine at the type-II′ β-turn in **5** is a result of the altered side-chain rotamer conformation.

The Ramachandran plot of the *i* + 2 residue (Fig. 5c) in the type-II′ β-turn of **2**, **3**, **4**, and **5** revealed that, as the strength of the n → π* interaction increases, the torsion angles of an amino acid in a non-helical region in the absence of the stabilizing intramolecular hydrogen bond are gradually altered to occupy the right-handed α-helical region. Thus, proline with a high propensity to engage in an n → π* interaction^11^ is a strong helix initiator.^48,49^ Hence, our result further supports the crucial role of the n → π* interaction in helix nucleation, as hypothesized earlier.^11^

### Implication of the n → π* interaction on protein stability

Since, an n → π* interaction results in a restricted *ϕ* torsion angle (Fig. 2e and f),^11^ we sought to examine the influence of the n → π* interaction on the conformational stability of **2**, **3**, **4**, and **5**. The midpoint of the thermal transition (*T*_M_), which is a measure of structural stability was determined by variable temperature CD (Fig. S10–S13†). **5** with the strongest C=O_*i*+1_ → C=O_*i*+2_ n → π* interaction displayed the maximum stability (*T*_M_) and **4** with no detectable C=O_*i*+1_ → C=O_*i*+2_ n → π* interaction showed the least stability (Fig. 4). We were surprised to note a very good correlation (Fig. S7†) between the *T*_M_ of these proteins and *E*_n→π*_ between C=O_*i*+1_ → C=O_*i*+2_, in the absence of the stabilizing C=O_*i*_⋯HN_*i*+3_ hydrogen bond (Fig. 4). An n → π* interaction rigidifies the β-turn by reducing the conformational entropy at the *i* + 2 residue, which is presumably responsible for the increased stability of the protein in solution. However, as the amino acid side-chains at the *i* + 2 residue of the type II′ β-turn are different in **2–5**, there might be additional factors that contribute towards the stability of these proteins. Therefore, we adopted an orthogonal strategy to validate the role of the n → π* interaction in protein stability.

By employing a prolyl-based torsion balance system, Raines *et al.* have shown that a thioamide (C=S_*i*_) engages in a stronger C=S_*i*_ → C=O_*i*+1_ n → π* interaction than amide C=O_*i*_.^20,50^ However, due to the longer C=S bond length (1.71 Å)^51^ and larger van der Waals radius of sulfur (1.85 Å),^52^ thioamide substitution perturbs the local secondary structure of proteins where the amide oxygen participates in a shorter hydrogen bond.^53–57^ On the other hand, thioamide substitution at a site where the amide oxygen is involved in a longer hydrogen bond or is solvent exposed, leads to minimal perturbation of the secondary structure.^53,55,57–59^ Therefore, we chose to substitute the solvent exposed C=O_*i*+1_ in the type-II′ β-turn of **2** by C=S_*i*+1_. The NBO analysis of the C=O_*i*+1_ to C=S_*i*+1_ substituted type-II′ β-turn in **2**, **3**, **4**, and **5** clearly indicated a significant enhancement in *E*_n→π*_, due to the amplified C=S_*i*+1_ → C=O_*i*+2_ n → π* interaction (Table S3†).

Thus, towards the synthesis of *i* + 1 thionated **GB1** (d-Val^t^–l-Ala; **2a**) (the thionated residue is denoted by superscript “t”), we obtained a clean 46-mer polypeptide up to the l-Ala_*i*+2_. However, on completion of the 56-mer **2a** on a solid support, following the acidolytic removal of protecting groups, the mass spectrum corresponded to a 45-mer fragment without the l-Ala_*i*+2_ (Fig. S16B†). To circumvent the undesirable peptide cleavage, we coupled the tetrapeptide Fmoc-Asn(Trt)-Gly-d-Val^t^-l-Ala-COOH and Fmoc-Asn(Trt)-Gly-d-Val-l-Ala-COOH onto two individual 45-mer polypeptides. After acidolytic cleavage, although we obtained the 49-mer oxo-polypeptide, the thio-tetrapeptide coupling repeatedly resulted in the 45-mer fragment without the l-Ala_*i*+2_ (Fig. S16C and D†). This suggests a spontaneous acid catalyzed cleavage of the peptide bond C-terminal to l-Ala_*i*+2_ in thioamidated **GB1**, **2a**.

**Fig. 6:**
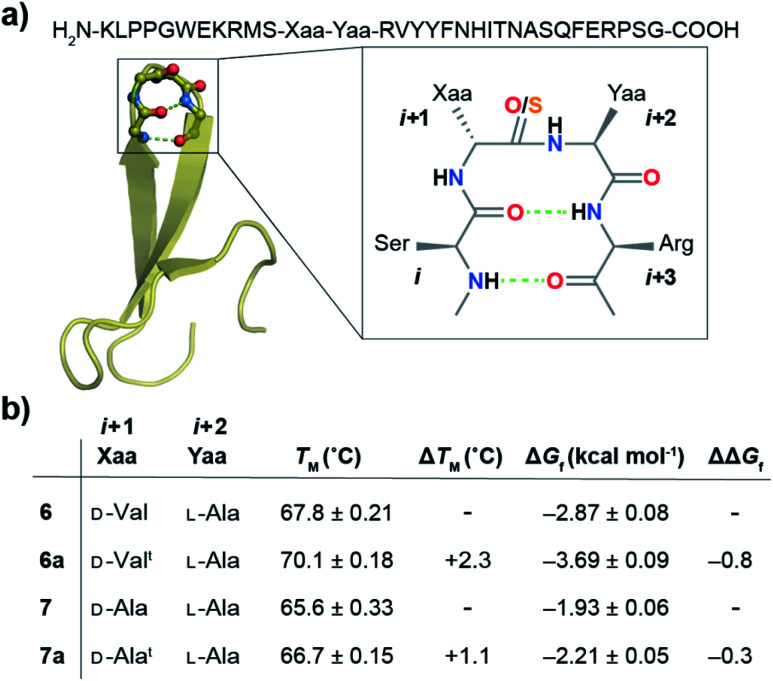
(a) Crystal structure of the Pin 1 WW domain (1ZCN) with –Asn–Gly– in loop 1 forming a type-I′ β-turn that has been modified to form a type-II′ β-turn in **6** and **7**. The n → π* interaction at the *i* + 2 residue is amplified by the C=S_*i*+1_ substitution in **6a** and **7a**. (b) The midpoint of the thermal transition (*T*_M_ ± S.D.) was derived from variable temperature CD. The free energy of folding (Δ*G*_f_) was obtained by fitting the guanidine hydrochloride denaturation (4 °C) curves to a two-state model. ΔΔ*G*_f_ = **6a** − **6** and **7a** − **7**.

**Fig. 7:**
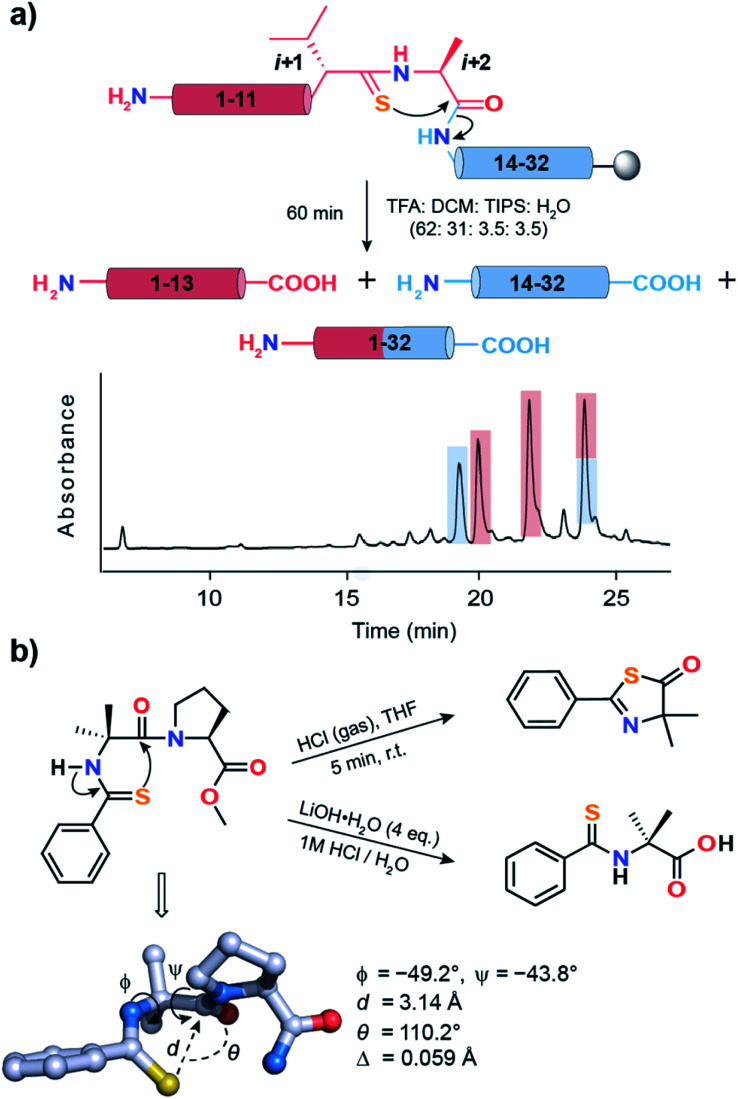
(a) Acid-catalyzed cleavage of **6a** yielding two N-terminal fragments (1–13), one C-terminal fragment (14–32) and the desired product (1–32). The two N-terminal fragments possibly result from the racemization of the l-Ala_*i*+2_ due to keto–enol tautomerization of the thiazolone intermediate.^63^ The polypeptides are color coded and are shown in the HPLC chromatogram. (b) Spontaneous acid-catalyzed cleavage of the thioacylated Aib–Pro dipeptide in aqueous solution.^62^ The thiazolone intermediate was trapped by passing HCl gas and characterized. The crystal structure of Ph-(C=S)-Aib-Pro-Aib-N(Me)Ph clearly depicting the n → π* interaction between C=S_*i*_ and C=O_*i*+1_ of Aib.

With numerous failed attempts to synthesize **2a**, we focused towards the 32-mer Pin 1 WW domain, a three stranded β-sheet protein that shows a cooperative two-state folding.^60,61^ The Pin 1 protein is amenable to loop modification that retains the global fold with alteration in its thermodynamic stability, making it an excellent model protein for structure-folding studies.^60^ We selected a Pin 1 variant with a type-I′ β-turn in loop 1 and substituted the –Asn–Gly– with d-Val–l-Ala– (**6**) and d-Ala–l-Ala– (**7**) (Fig. 6a) to adopt a type-II′ β-turn that was confirmed by characteristic NOEs at the β-turn (Fig. S29†). Subsequently, we synthesized Pin 1 variants with thioamidation at the *i* + 1 site (d-Val^t^–l-Ala; **6a** and d-Ala^t^–l-Ala; **7a**). Remarkably, the acidolytic removal of the protecting groups to obtain **6a** resulted in both the desired product and the N- and C-terminal fragmented peptides resulting from the nucleophilic attack of d-Val C=S_*i*+1_ onto l-Ala C=O_*i*+2_ (Fig. 7a) as observed in **2a**.

An identical fragmentation was reported by Heimgartner *et al.* during the aqueous acidolytic workup of the thioacylated Aib–Pro dipeptide (Fig. 7b), towards the synthesis of Ph-(C=S)-Aib-Pro-Aib-N(Me)Ph.^62^ However, by bubbling HCl gas through the dipeptide in THF, the thiazolone intermediate could be characterized, which results from the nucleophilic attack of Ph C=S_*i*_ onto Aib C=O_*i*+1_. To our excitement, the crystal structure of the final product Ph-(C=S)-Aib-Pro-Aib-N(Me)Ph revealed the C=S_*i*_ → C=O_*i*+1_ n → π* interaction, leading to a high degree of pyramidalization, *Δ* = 0.059 Å at Aib C=O_*i*+1_, a firm indicator of the n → π* interaction.^19^ Thus, the directional (*i* + 1 → *i* + 2) fragmentation observed in **2a** (Fig. S16†), **6a** (Fig. 7a) and **7a** (Fig. S17B†) is a chemical signature of the amplified n → π* interaction between C=S_*i*+1_ and C=O_*i*+2_.

Next, we assessed the folding of Pin 1 variants **6**, **6a**, **7**, and **7a** in sodium phosphate buffer (pH 7.4). All the Pin 1 proteins showed the characteristic 227 nm maximum in the CD spectrum, indicating the presence of a folded protein with β-sheets (minimum centered around 215 nm) (Fig. S22A–S25A†). The virtually identical H^α^ chemical shift perturbation deduced from TOCSY and NOESY experiments indicated that a single atom substitution (O to S) at the solvent exposed C=O_*i*+1_ did not lead to major structural perturbation in **6a** and **7a** (Fig. S28†). We next performed thermal and chemical denaturation (Fig. S22–S25†) to understand the effect of the amplified n → π* interaction. The proteins showed a two-state unfolding and we were delighted to note that the C=S_*i*+1_ → C=O_*i*+2_ n → π* interaction enhanced the stability of **6a** by 0.8 kcal mol^−1^ and **7a** by 0.3 kcal mol^−1^ (Fig. 6b).

The increased stability arises from the reduced conformational flexibility of the amino acid residue engaged in an n → π* interaction, a feature that is analogous to the ring constraint in proline, which restricts its conformational space compared to other amino acids and increases protein stability by reducing the entropy of the unfolded state.^64,65^ An n → π* interaction restricts the conformational space of an amino acid residue with the adoption of torsion angles as depicted in the Ramachandran plot of the β-turn residues (Fig. 2c and d). This would also be expected in an amplified n → π* interaction by thioamide substitution. The adoption of such torsion angles is favorable at the *i* + 1 and *i* + 2 positions of a β-turn (Fig. 2a). Furthermore, since β-branched amino acids restrict the backbone conformation more than the unbranched residues,^65^ the C=S_*i*+1_ → C=O_*i*+2_ n → π* interaction stabilizes **6a** more than **7a**.

Thus, our results in the Pin 1 WW domain re-emphasize the role of the amino acid side-chain in tuning the n → π* interaction energy. Not only the side-chain rotamer of the amino acid involved in an n → π* interaction dictates its strength (Fig. 4), the steric interactions imposed by the amino acid side-chain of the donor carbonyl oxygen_(*i*)_ (C=S_*i*+1_ in this case) (Fig. 6) can also influence an n → π* interaction.

## Conclusions

In summary, our bioinformatic analysis indicates that the reduced conformational freedom of the donor C=O_*i*_ by the intramolecular C=O_*i*_⋯HN_*i*+3_ hydrogen bond in β-turns is associated with the high abundance of n → π* interactions at the *i* + 1 residue, whereas, the absence of the intramolecular hydrogen bond, constraining either the C=O_*i*+1_ or C=O_*i*+2_ results in conformational flexibility of the *i* + 2 residue, which could be restricted by introducing an C=O_*i*+1_ → C=O_*i*+2_ n → π* interaction. The experimental results at the *i* + 2 residue of the type-II′ β-turn in **GB1** variants suggest that amino acid side-chain identity and the rotamer conformation can modulate the strength of an n → π* interaction. Although, it is challenging to estimate the exact contribution of this energetically subtle interaction towards the global stability of the protein, we note that the altered rotamer conformation as a result of local structural changes can amplify/weaken an n → π* interaction affecting the backbone torsion angles (*ϕ*, *ψ*), thereby influencing its stability. With an enhanced n → π* interaction in the absence of the stabilizing intramolecular hydrogen bond, we observe a clear shift of amino acid torsion angles (*ϕ*, *ψ*) from a non-helical to the right-handed α-helical region. It is worth noting that the *i* → *i* + 1 directionality (N-term → C-term) associated with the n → π* interaction coincides with the formation of the productive helix nucleus at the N-terminus of a polypeptide,^66–68^ highlighting an important contribution of the n → π* interaction towards helix nucleation. Furthermore, the recent report of a long-range n → π* interaction in stabilizing the α-helical conformation of a synthetic peptide in water, re-emphasizes the potential of this noncovalent interaction in engineering helical structures.^69^

To conclusively demonstrate the influence of the n → π* interaction on protein stability, we chose to amplify this weak noncovalent interaction by thioamide substitution. Since a strong n → π* interaction induces a “kink” in the polypeptide backbone by optimizing the *ϕ*, *ψ* torsion angles suitable for orbital overlap, and thereby reducing the conformational entropy at the β-turn, the thioamide substitution increased the protein stability. It is worth noting that thio-Gly465 in the natural protein methyl-coenzyme M reductase, which is suggested to stabilize the protein secondary structure near the active site, induces a kinked conformation (*ϕ*, *ψ* = −68.5°, −47.2°) by engaging in an n → π* interaction with C=O of Phe466 (Fig. S30†).^70,71^ With the recent advancement in ribosome mediated incorporation of thioamide into proteins and polypeptides, thioamide substitution could be potentially utilized to stabilize turns and enhance protein stability,^72,73^ aided by exogenous factors like salt concentration^11^ and solvation by water molecules^69^ that have been shown to influence the n → π* interaction in protein secondary structures.

## Conflicts of interest

There are no conflicts to declare.

## Supplementary Material

SC-011-D0SC03060K-s001

SC-011-D0SC03060K-s002

SC-011-D0SC03060K-s003

SC-011-D0SC03060K-s004

SC-011-D0SC03060K-s005
