# Supplementary material for: Increasing protein stability by engineering the n → π* interaction at the β-turn
Source: Chem Sci. 2020 Jul 30;11(35):9480–7. doi: 10.1039/d0sc03060k (PMC8161691; doi:10.1039/d0sc03060k)
Supplement: SC-011-D0SC03060K-s005 [file SC-011-D0SC03060K-s005.pdf]

Electronic Supplementary Material (ESI) for Chemical Science.
This journal is © The Royal Society of Chemistry 2020

Supplementary Information

Increasing protein stability by engineering n → π* interaction at the β-turn

Bhavesh Khatri,† Puja Majumder,† Jayashree Nagesh,‡ Aravind Penmatsa,†* and Jayanta Chatterjee†*

†Molecular Biophysics Unit, Indian Institute of Science, Bangalore, 560012, India.

‡Solid State and Structural Chemistry Unit, Indian Institute of Science, Bangalore 560012, India.

Table of Contents:

1. Materials and Methods…………………………………………………………………...S2-S7
2. Table S1 – Data used to generate figure **3B-3D**………………………………………...S8-S19
3. Analytical HPLC chromatograms and ESI MS analysis of purified proteins **GB1** and **1-5**…..S20-S25
4. Far-UV CD spectra and thermal denaturation of **GB1** and **1-5**………………………………S26-S29
5. Table S2 – X-ray diffraction data and refinement statistics for proteins **2-5**………………………S30
6. The *m*F*o*-*D*F*c* omit maps of proteins **2-5**………………………………………………......S31
7. Intramolecular hydrogen bonding at the loopL1 in **2-5**……………………………………….S32
8. Table S3 – Overlap of n and π* orbitals in proteins **2-5**…………………………………………S33
9. Threonine side-chain rotameric conformation in β-turns…………………………………......S34
10. Synthetic attempts of **2a**…………………………………………………........................S35-S38
11. Analytical HPLC chromatograms and ESI MS of Fmoc-Asn(Trt)-Gly-D-Val^t-Ala-COOH….S39-S41
12. The acid-catalyzed cleavages of **6a** and **7a**…...…………………………………………S42-S43
13. Analytical HPLC chromatograms and ESI MS analysis of purified Pin1-WW variants……..S44-S47
14. Far-UV CD spectra, thermal denaturation, and chemical denaturation of Pin1-WW variants..S48-S51
15. ^{1}H NMR overlay of Pin1-WW variants……………………………………………………S52-S53
16. Chemical shifts of Pin1-WW variants……………………………………………………...S54
17. Chemical shift deviation of Pin1-WW variants………………………………………….S55
18. Characteristic NOEs for the loop1 region in peptide **6**………...……....…………………………S56
19. n→π* interaction between thioglycine and phenylalanine in a natural protein Methyl-coenzyme M reductase……………………………………………………………………………………….S57
20. References..……………………………… ……………………………….........…S58

Materials and Methods:

Fmoc- and orthogonally protected amino acids, 1-hydroxybenzotriazole (HOBt), O-(6-chlorobenzotriazol-1-yl)-*N*,*N*,*N'*,*N'*-tetramethyluronium hexafluorophosphate (HCTU), 2-Chlorotritylchloride polystyrene (2Cl-TCP) were purchased from GL Biochem, Shanghai. 4-Nitro-*o*-phenylenediamine, Lawesson's reagent, sodium nitrite, *N*,*N'*-Diisopropylcarbodiimide (DIC), *N*,*N*-diisopropylethylamine (DIPEA), trifluoroacetic acid (TFA), trifluoroethanol (TFE), triisopropylsilane (TIPS), dimethylformamide (DMF), dichloromethane (DCM), ethyl acetate (EtOAc), hydrochloric acid (HCl), sodium bicarbonate ($NaHCO_3$), sodium chloride (NaCl), sodium sulfate (Na_2SO_4), anhydrous tetrahydrofuran (THF), 1,8-Diazabicyclo[5.4.0]undec-7-ene (DBU), piperazine, glacial acetic acid, calcium hydride, piperidine and Guanidine hydrochloride were purchased from Sigma-Aldrich. All the above reagents were used as commercially supplied. Solvents for RP-HPLC were purchased as HPLC grade and used without further purification. Dichloromethane was dried with calcium hydride. All the other solvents were used as commercially supplied. All the reactions were performed in oven-dried glass apparatus. Reactions on solid-support were carried out in plastic syringes (10 ml) fitted with a frit column plate purchased from Torviq (USA).

Instrumentation:

High-resolution mass spectra were recorded on a Bruker Daltonics ESI Q-TOF (Maxis Impact) mass spectrometer. ESI mass spectra were recorded in positive ion mode on a HCTultra ETD II ion-trap spectrometer (Bruker Daltonics, Germany). Nuclear magnetic resonance (NMR) spectra were recorded in either on a 700 MHz, or a 500 MHz Bruker Avance spectrometer (Bruker, Karlsruhe, Germany). Circular Dichroism (CD) spectra were acquired on a JASCO-715 spectropolarimeter using 0.1cm path-length cuvette. The CD spectra were averaged over 3 scans and the baseline correction was done by subtraction of the spectrum with the appropriate blank solution. Fluorescence spectra were acquired on JASCO FP-6300 Spectrofluorometer using 0.1cm path length cuvette. The spectra were averaged over three scans. Analytical RP-HPLC was performed on a Shimadzu UFLC system equipped with Prominence Diode Array (PDA) detector using an analytical column (Phenomenex C18, 250 mm x 4.6 mm I.D., 5 µm) at a flow rate of 1 mL min^{-1}. Purifications were performed using a semi-preparative column (Phenomenex C18, 250 mm x 10 mm I.D., 5 µm) at a flow rate of 4 mL min^{-1}.

Synthesis

Proteins (**GB1** and **1-5**) were synthesized on Rink Amide Am resin (0.8 mmolg^{-1}) on 150 mg scale using standard Fmoc-based strategy. Coupling reactions were performed by using standard coupling reagents (2.5 equiv HOBt, 2.5 equiv DIC) and Fmoc-amino acid (2.5 equiv) in DMF. Following a two-minute preactivation, the activated amino acid was added to the resin and vortexed for 2 h. Deprotection reactions were carried out twice with with 20% piperidine in DMF (5 min + 15 min). The resin was washed two times with 3 ml of DMF for 60 sec between each cycle. After the final deprotection step, the resin was washed with 3 ml of dichloromethane (DCM) followed by 3 ml of methanol. The global deprotection of protected proteins were carried out with 62% TFA, 31% DCM, 3.5% triisopropylsilane and 3.5% water for 2 h. Deprotected crude protein was precipitated in chilled ether. The white solid was pelleted by centrifugation and dissolved in 7 M Guanidine HCl for purification. Purifications were performed on a Shimadzu UFLC system equipped with Prominence Diode Array (PDA) detector using a reversed-phase Phenomenex Jupiter C18 300 Å analytical column (250 mm x 4.6 mm I.D., 5 µm) at a flow rate of 1 mL min^{-1}.

Pin1 WW domain variants were synthesized on 2Cl-TCP resin (1.3 mmolg^{-1}), using standard Fmoc-based chemistry. The C-terminal amino acid residue, Fmoc-Gly-OH (1.25 equiv) was loaded on to the resin with 2.5 equiv DIPEA in anhydrous DCM at room temperature. After loading the first amino acid, the remaining unreacted trityl chloride groups bound to the solid-support were capped using methanol (200 µl/100 mg resin) for 15 min. Next, the resin was thoroughly washed with DCM (3 times), 1:1 DCM-methanol (3 times) and methanol (3 times) and finally dried under vacuum. The loading capacity was estimated from the dry weight of the resin, which ranged from 0.4-0.6 mmolg^{-1}. The elongation of the rest of the peptide was performed on 150 mg (0.09-0.12 mmol) scale with DIC/HOBt as the coupling agents (2.5 equiv). Fmoc-deprotections for **6** and **7** were carried out with 20% piperidine in DMF (5 min + 15 min) and 2% DBU + 5% piperazine in DMF (60 sec x 2) was used for thiopeptides **6a** and **7a** after the incorporation of thionated amino acid. The resin was washed two times with 3 ml of DMF for 60 sec between each cycle. The global deprotection of protected proteins were carried out with 62% TFA, 31% DCM, 3.5% triisopropylsilane and 3.5% water for 60 min. Deprotected crude protein was precipitated in chilled ether. The white solid was pelleted by centrifugation and dissolved in water for purification.

General method for the synthesis of the aminoanilide precursors:

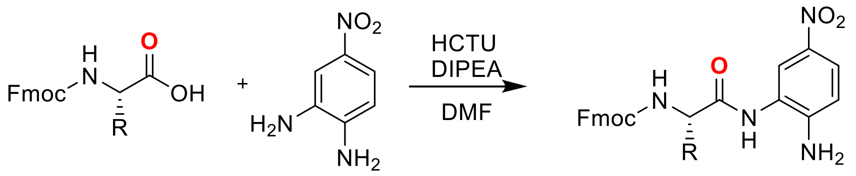

To a 0.1-0.3 M solution of Fmoc-Xaa-OH (1 equiv) in DMF, HCTU (1.2 equiv) and DIPEA (1.5 equiv) were added followed by 4-Nitro-*o*-phenylenediamine (1.1 equiv). The reaction was stirred vigorously for 6 h

until TLC (Thin Layer Chromatography) indicated the complete consumption of Fmoc-Xaa-OH. The reaction mixture was poured into cold water and filtered using sintered disc filter-funnel. The yellow solid obtained after filtration was dissolved in ethyl acetate and washed with 1N HCl (2 times) and saturated $NaHCO_3$ (2 times). The organic fractions were combined, dried over Na_2SO_4 and evaporated to dryness, yielding respective aminoanilides as a yellow solid. It was used for the next step without further purification.

General method for the synthesis of the thioaminoanilide precursors:

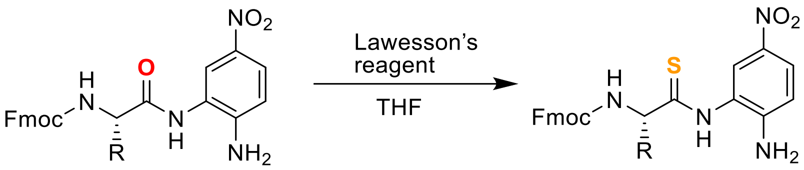

To a 0.1-0.3 M solution of aminoanilide in dry THF, 1.5 equiv of Lawesson's reagent was added. The reaction mixture was stirred at 30 °C for 6-8 h. The progress of the reaction was monitored by TLC. After completion, the reaction mixture was filtered through sintered disc filter-funnel, evaporated to dryness and dissolved in EtOAc. The solution was washed with saturated $NaHCO_3$ (2 times), dried over Na_2SO_4 and evaporated to dryness, yielding the respective thioaminoanilides as a yellow solid. It was used for the next step without further purification.

General method for the synthesis of the thiobenzotriazolide precursors:

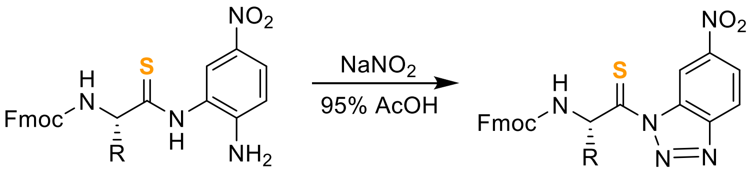

To a 0.1-0.3 M solution of thioaminoanilide in glacial acetic acid containing 5 % water, 1.5 equiv of sodium nitrite was added in portions at 4 °C. The reaction mixture was stirred at 4 °C for 30 min. The progress of the reaction mixture was monitored by TLC. Upon completion, the reaction mixture was poured into cold water and centrifuged. The supernatant was discarded, and the light orange precipitate was dissolved in DCM. The DCM solution was washed with saturated $NaHCO_3$ (2 times), saturated NaCl (once) and dried over Na_2SO_4. This solution was directly utilized for incorporating the thiobenzotriazolides onto the solid support.

Incorporation of the thiobenzotriazolide on solid-support

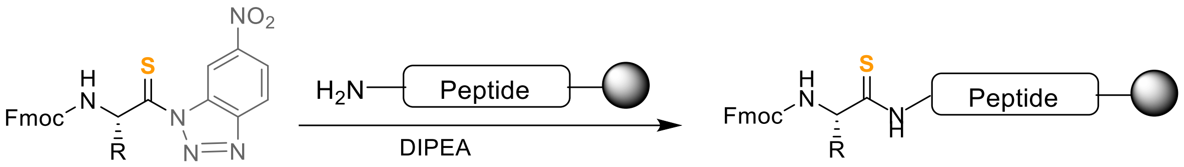

The Fmoc deprotected peptide was swollen in dichloromethane in a fritted syringe prior to the coupling. The solution of thiobenzotriazolide precursor (2 equiv with respect to the resin loading) in dichloromethane was taken into the syringe along with (0.5 equiv) of DIPEA. The syringe was rotated in a mechanical rotor for 45 min under protection from light. The step was repeated once more. Finally, the resin was washed 5× with dichloromethane.

Circular Dichroism Spectroscopy

Far-UV CD spectra of **GB1** and **1-5** were recorded at 40 μM concentration in 20 mM sodium phosphate buffer (pH 7.4). Scans were carried out at 25 °C over the range of 190-260 nm with 0.5 nm increments and a 2 nm bandwidth. Thermal unfolding experiments were monitored at 220 nm over a temperature range of 4-98 °C with 0.5 °C interval in 20 mM sodium phosphate buffer (pH 7.4). The samples were equilibrated for 2 minutes at each temperature. Temperature-dependent CD data were fit to a two-state unfolding model to obtain melting temperature (T_M). Thermal unfolding experiments were repeated three times for each sample.

Far-UV CD spectra of Pin1 WW variants were recorded at 10 μM concentration in 20 mM sodium phosphate buffer (pH 7.4). Scans were carried out at 25 °C over the range of 190-400 nm with 0.5 nm increments and a 2 nm bandwidth. Thermal unfolding experiments were monitored at 227 nm over a temperature range of 4-98 °C with 0.5 °C interval in 20 mM sodium phosphate buffer (pH 7.4). The samples were equilibrated for 2 minutes at each temperature. Temperature-dependent CD data were fit to a two-state unfolding model to obtain melting temperature (T_M).[1] Thermal unfolding experiments were repeated three times for each sample.

Fluorescence

Stability of Pin1 WW variants were assessed by chaotrope denaturation, where different concentrations of guanidinium hydrochloride (0-7 M) were titrated in 20 mM sodium phosphate buffer (pH 7.4) solution having equal protein concentration of 2.5 μM. The samples were equilibrated for 2 hours at 4°C. The samples were excited at 284 nm and the emission spectra were collected from 310-380 nm at each guanidinium hydrochloride concentration. The emission signal intensity at 338 nm was used to generate the denaturation

curves. The data obtained were fit to a two-state model as previously described.[2] GdnHCl-induced denaturation experiments were repeated three times for each sample.

NMR Acquisition

Pin1 WW analogs (**6-7**, **6a**, and **7a**) were dissolved in 20 mM sodium phosphate buffer (pH=7.4) having 10% D_2O. In all the compounds, 0.1% TMSP was used as an internal standard (δ = 0 ppm). NMR were obtained using a concentration of 100 μM at 15°C. Standard Bruker pulse sequences zgesgp for ^{1}H, mlevesgpph/dipsi2rcesgpph (60 ms mixing time) for TOCSY, and noesyesgpph (200 ms mixing time) for NOESY were used to acquire the NMR data. Two-dimensional data were obtained using 2048 data points in the direct dimension and 512 data points in the indirect dimension. All NMR data were processed using iNMR (www.inmr.net), and the 2D NMR data were analyzed with SPARKY.

PDB analyses

High-resolution (≤ 2.0 Å) and non-redundant protein X-ray crystal structures were collected from the PDB using ccPDB with data downloaded on 31 March 2018.[3] Total 500 β-turns representing type I, type II and their mirror images type I' and type II' were analyzed manually. The presence of n→π* interaction was considered in which (1) the distance between $O_i \cdots C{=}O_{i+1}$ was less than the sum of their van der Waals radii (d ≤ 3.2 Å), and (2) the angle $O_i \cdots C{=}O_{i+1}$ was 99° ≤ θ ≤ 119°.

Computational analyses

All crystal structures were subjected to geometry optimization with respect to the H atoms, since the position of these are the least certain from crystallographic assignments. The model chemistry wB97XD[4] /6-31+G(d,p) was used to do the geometry optimization as well as to carry out the NBO analysis. The interaction energies are calculated using the DELETION (NBO-DEL) approach, where the overall change in electronic energy due to the removal of a particular donor-acceptor interaction is obtained by rediagonalization of the Fock matrix.[5] All ab initio computations have been carried out using Gaussian[6] and NBO 6.0[7] suite of programs. All the overlap figures have been generated using the Jmol program with an orbital surface isovalue of 0.02e/A^3. Since crystallographic structures are not available for the thio analogs, we have carried out the analysis by simply replacing the *i*+1 C=O with C=S without further optimization of the structure; we assume that the structural perturbations are negligible, and we only wish to explore trends in the energies upon the single atom substitution.

Protein Crystallization

Crystallization was carried out by hanging-drop vapour diffusion method in 24 well hanging drop plates at ~21°C. The concentration of the sock solutions of lyophilized GB1 variants dissolved in water was around 18 mg/ml. The crystals of chemically synthesized GB1 variants were grown in the following conditions, 0.2 M $CaCl_2$, 0.1 M sodium acetate (pH 4.6), 30% Isopropanol for protein **2**, protein **5** crystals were grown in 0.1 M sodium cacodylate (pH 5.5), 20% PEG 4000, In case of protein **3** the crystals were obtained in 0.1 M sodium cacodylate (pH 6), 20% PEG 4000 and protein **4** crystals were grown in 0.1 M MES (pH 6), 20% PEG 4000. We observed large rod-shaped crystals of each variant within 2 days at 1:1 (protein: condition) drop ratio. A single crystal of each protein was flash frozen in liquid nitrogen after cryoprotection in well buffer supplemented with 30% v/v glycerol.

X-ray diffraction data collection and structure determination

The X-ray diffraction data of synthetic GB1 proteins **2**, **3**, **4**, and **5** were collected at home source using CuKα radiation (1.5417 Å), generated by a Rigaku FR-E+ Superbright X-ray generator. The diffraction data was collected at 100 K using a Rigaku R-axis IV detector, with 1° oscillation range. Proteins **2**, **3**, **4**, and **5** crystal-to-detector distance was kept at 135 mm, 120 mm, 140 mm and 135 mm respectively. The crystals were indexed, integrated and scaled using XDS[8] and CCP4 software suite.[9] The data collection and processing statistics are given in Table S2. The structures were determined by molecular replacement using the program PHASER of CCP4 suite. The crystal structure of GB1 (PDB ID 2QMT) was used as a search model. The solution of proteins **2**, **3**, and **5** displayed a single molecule in the asymmetric unit in the space group $P3_221$ with data collected to a maximal resolution of 1.95 Å, 1.84 Å and 1.73 Å, respectively. The structure solution of protein **4** displayed two molecules in the asymmetric unit in the space group C2 with 1.89 Å resolution. The models obtained from molecular replacement were built using COOT[10] and refined by PHENIX refine[11] iteratively. The refinement statistics are given in Table S2. The final R_{work}/R_{free} values of 2, 3, 4 and 5 were 20.6/24.9, 21.9/24.2, 27.8/32.8 and 16.6/18.6 respectively. The comparatively higher R_{work}/R_{free} values were obtained in case of **4** because the lattice had translational pseudo-symmetry with a peak in Patterson map at 0, 0, 0.5. The Coordinates and structure factors for synthetic GB1 variants have been deposited in the Protein Data Bank under accession codes 6L9B (**2**), 6L9D (**3**), 6LJI (**4**), and 6L91 (**5**).

Table S1 – Data used to generate Figure 3B-3E.

type I β-turn

PDB_ID	Chain	Residue No.	Sequence	i+1		d (Å)	θ (°)	i+2		d (Å)	θ (°)	C=O$_i\cdots$N$_{i+3}$ (Å)
				φ (°)	ψ (°)			φ (°)	ψ (°)			
1T8A	A	20-23	DTEG	-56.3	-29.8	2.9	116	-80.9	3.5	3.2	137	2.76
		92-95	NAKL	-62	-24.9	3.1	119	-91.1	-30.9	3.4	109	3.31
3B1B	A	28-31	SPDS	-63	-21.3	3	120	-84.4	5.9	3.4	138	2.95
		42-45	HGLN	-50.8	-40.9	2.8	107	-113.8	29	3.8	149	2.81
		46-49	GENW	-58.3	-30.6	2.9	114	-101	21.8	3.7	149	3.16
		53-56	DGAG	-63.2	-24.8	3	117	-93.4	3.8	3.5	137	2.96
		60-63	VCKT	-50.7	-46.3	2.8	102	-94.8	-5.2	3.5	129	3.19
		135-138	NQTN	-62.7	-15.3	3	124	-75.9	-18.9	3.2	120	2.9
		139-142	RIVD	-64.6	-20.1	3	117	-89.4	3	3.4	134	2.94
		147-150	RPND	-49.6	-28.8	2.8	117	-103.6	8.3	3.6	136	2.93
		214-217	NELL	-59.3	-24.3	2.9	117	-60.6	-29	3	113	3.03
1Y20	A	23-26	MSDG	-55.4	-33.6	2.9	111	-86.2	4.2	3.4	138	2.95
		33-36	TVNG	-67.9	-18.8	3.1	121	-91.5	-8.5	3.5	128	3.06
		87-90	VADG	-57.5	-42.3	3	104	-88.2	3.3	3.4	134	3.11
		158-161	GIND	-67.1	-18.8	3.1	121	-97.4	8.6	3.6	139	3.17
		235-238	KCDL	-62.4	-15.9	3	126	-91	-5.5	3.5	130	3.01
1K2A	A	62-65	CPSN	-58	-29.7	2.8	112	-88.7	-12.7	3.3	125	3.14
		115-118	DQRR	-57.9	-47.4	2.9	100	-79.4	-37.1	3.2	106	3.51
2BC3	A	34-37	GADG	-73.8	-21.9	3	114	-85.4	3.1	3.7	144	3.26
		115-118	TEAN	-61.1	-37.5	2.8	106	-67.1	-28.7	3	114	3.12
1BZD	A	18-21	DAVR	-80.3	-13.5	3.4	127	-86.3	-37.3	3.5	106	3.92
		36-39	AADD	-45.1	-37.2	2.8	112	-82.4	22.2	3.4	158	2.64
		50-53	SESG	-61.5	-31.9	3	114	-88.5	6.8	3.4	141	2.92
		60-63	TEEE	-64.5	-36.3	3	108	-72.4	-29.5	3.1	106	3.32
		112-115	SPYS	-48.5	-46.9	2.8	101	-109.2	16.5	3.7	142	3.17
1DUG	A	33-36	ERDE	-45.3	-40.9	2.8	110	-101.8	14.1	3.7	141	2.92
		43-46	KKFE	-36.1	-50.8	2.7	105	-98.9	20.4	3.6	146	2.71
		59-62	DGDV	-68.3	-14.4	3.1	124	-113.1	-25.9	3.8	114	3.75
		111-114	SKDF	-70.6	-22.8	3.1	116	-95.6	21.7	3.5	149	3.16
		141-144	YLNG	-53.2	-35.4	2.9	111	-110.7	20.6	3.7	144	2.98
		165-168	DPMC	-52.1	-19.3	2.9	125	-99	-6.4	3.6	127	2.89
2DC3	A	95-98	NLHD	-61.2	-23.1	2.9	121	-71.3	-9.2	3.2	132	2.81
		116-119	LKHK	-86.5	-52.8	3.4	96	-90.3	-15.3	3.4	121	4.71
		177-180	NATT	-63.9	-25.3	3	115	-83.5	-13.5	3.3	125	3.18
3SA0	A	22-25	GPRY	-80.6	-19.3	3.3	120	-66.5	-36.3	3	105	3.99
		45-48	NVNK	-98.9	-57.5	3.6	94	-81.4	-3.4	3.3	132	4.64
		80-83	HENI	-59.3	-26.3	3	121	-108.2	18.5	3.7	143	3.19
		94-97	TIEQ	-60.5	-37.4	2.9	105	-60.4	-35.6	2.9	110	3.22
		168-171	FGLA	-66.2	-21.7	3	120	-109.2	9.4	3.8	136	3.13
		279-282	FPNA	-63.4	-18.5	3	124	-100	0.9	3.6	134	3.06
		297-300	NPHK	-68.6	-25.6	3	116	-67.7	-30.4	3.2	109	3.09

		313-316	LEQY	-59.6	-27	2.9	116	-70.5	-23.9	3	118	2.99
		335-338	LDDL	-79.9	-28.1	3.3	113	-96.9	31	3.5	156	3.93
1B11	A	30-33	DTGA	-65.9	-11.3	3.1	128	-93.4	0	3.4	134	3.04
		77-80	DENY	-49.8	-41.4	3	110	-81.4	-4.7	3.3	133	3.02
		92-95	LEDN	-59.8	-23.7	3.1	121	-94	9.2	3.4	138	3.22
1LZ6	A	39-42	NTRA	-69	-22	3.1	119	-96.9	6.7	3.4	135	3.34
		47-50	AGDR	-55.8	-41.7	3.1	109	-128.5	5.5	4	130	3.86
		55-58	GIFQ	-59	-34.7	3	112	-103.5	3.6	3.7	132	3.08
		94-97	ACAK	-69.9	-43.8	3.1	100	-59.8	-39.2	3	104	3.45
1D7O	A	66-69	GKFD	-63.4	-27.6	2.9	111	-100.9	10.6	3.5	140	3.18
		142-145	EVSK	-51.2	-27.2	2.9	117	-85.4	0.9	3.2	135	2.81
		281-284	ASAI	-51.8	-20.1	2.9	126	-104.9	7.6	3.6	139	2.9
		304-307	SPVF	-60.2	-18.8	3	123	-66.9	-22.9	2.9	116	2.95
4GF6	A	48-51	CTTG	-84.1	-11.7	3.3	125	-92.7	-3.5	3.5	129	3.6
		88-91	MPEG	-83.3	-11.1	4.5	154	-61.3	-37.5	3	108	5.75
		131-134	KEAG	-48.7	-39	2.9	112	-113.8	20.9	3.8	144	2.99
		135-138	NILG	-84.8	-66.3	3.4	85	-68.8	-22.7	3	116	4.44
		171-174	IADG	-57.2	-30.4	2.9	115	-82.2	2.8	3.3	136	2.91
		210-213	DPNE	-59.2	-18.4	3	122	-92.3	-1.5	3.5	133	2.94
1LQA	A	20-23	TMTF	-67.2	-11.1	3	127	-92.6	-1.1	3.4	129	2.86
		50-53	AEMY	-65	-27.2	2.9	112	-83.7	-2.4	3.3	130	3.28
		58-61	RPET	-52.6	-36	2.9	111	-60.4	-33	2.9	111	3.14
		209-212	SLLN	-51.6	-31.2	2.8	113	-88.7	-13.5	3.5	127	2.98
		234-237	SCLG	-76.3	-12.3	3.1	124	-105.2	13.7	3.7	142	2.98
		243-246	GKYL	-67.7	-13.6	3	125	-108.4	8.3	3.7	138	3.12
		299-302	QPFV	-70.8	-17.4	3.1	119	-91.2	-3.2	3.5	128	3.16
		263-266	TRYS	-55.4	-30.8	2.8	112	-115.3	29.3	3.9	147	3.05
1U1D	A	99-102	QPHI	-62.7	-29.5	3.1	114	-80.6	-13.8	3.3	125	3.31
		125-128	PLEF	-54.2	-33.1	2.9	114	-63.7	-22.2	3.1	121	2.97
		171-174	TYSG	-60	-48.7	3	98	-80.4	-33.5	3.3	109	3.58
		222-225	NRTQ	-67.3	-15.5	3.1	126	-90	1.9	3.5	135	3.08
4E3X	A	83-86	SPFN	-59.7	-32.2	2.9	111	-93	-2.9	3.5	132	3
		137-140	GPRR	-67.6	-21.1	3	120	-107	2.8	3.7	133	3.28
		209-212	PFNF	-71.7	-25.3	3.1	119	-88	-14.7	3.3	123	4.3
		276-279	SEHL	-62.8	-14.5	3	124	-96.1	2.6	3.5	135	3.2
		302-305	LDRF	-58.2	-38.6	2.9	107	-79.6	-25.7	3.2	116	3.56
		324-327	HSSA	-60.5	-21.5	3	121	-83.2	-0.1	3.4	136	2.94
		347-350	KCSA	-60.8	-19.1	3	124	-102	-3.6	3.7	131	3.14
		379-382	DPAE	-79.4	-19.9	3.2	120	-79	-35	3.3	107	3.47
		410-413	SPSL	-69.1	-17.6	3	120	-93.7	-7.5	3.5	130	3.22
		438-441	DPQE	-69.2	-11.4	3	125	-117.8	21.7	3.8	143	3.22
		497-500	LRNA	-47.9	-38.6	2.9	112	-112	7.8	3.8	134	2.93
		534-537	GPHY	-76.7	-10.9	3.2	127	-99.1	-15.8	3.5	121	3.71
		559-562	YSYM	-65.8	-27.5	3	115	-73.9	-14.4	3.1	124	3.12
1PZS	A	09-12	APDG	-51.3	-30.7	2.9	114	-85.2	2	3.4	136	2.84
		60-63	APTG	-49.2	-37.7	2.9	110	-100.7	7.8	3.6	138	3.02
		86-89	ASGD	-53.1	-33.2	2.9	111	-89.4	2.2	3.4	135	3.04

		96-99	RGDG	-54.9	-30.5	2.9	114	-88.8	4.2	3.5	137	2.97
		136-139	PPER	-65.7	-18.7	3.1	120	-75.2	-18.8	3.2	121	3.15
		142-145	QVNG	-64	-18.6	3	121	-79.5	-7.1	3.4	130	2.83
2H26	A	20-23	NSTW	-64	-19.7	2.9	121	-111.5	1.5	3.7	129	3.11
		42-45	SDSG	-69.9	-47.3	3	96	-79.2	-25.2	3.3	115	3.57
		51-54	KPWS	-54.5	-28.7	2.8	113	-100.1	11.4	3.6	139	3.02
		86-89	GDFQ	-59.2	-32.2	3	116	-93.4	6.6	3.5	138	2.99
		227-230	QQGT	-62.9	-26.4	2.9	117	-75.9	1	3.3	137	2.98
		238-241	NANW	-69	-19.7	3.1	120	-107.2	37.5	3.8	158	4.2
2WM5	A	25-28	ELGS	-63.4	-22.3	3	120	-89.1	5.7	3.6	143	2.96
		84-87	HPDI	-67	-18.2	3	118	-67.5	-23.5	3	115	2.99
		156-159	NPDR	-52.7	-31.1	2.9	112	-74.1	-6.2	3.3	130	2.84
		202-205	DPDL	-72.5	-12.3	3.1	125	-89	-9.8	3.4	127	3.09
		355-358	DPWT	-67.6	-17.6	3	121	-95.9	8.2	3.5	138	2.97
		369-372	LGFG	-67.8	-23.3	3	109	-101.6	22.3	3.6	150	2.86
		379-382	CLGA	-69	-24	3	109	-94.4	11.5	3.5	143	3.26
1CHP	A	42-45	FKNG	-51.4	-34	2.8	108	-70	-4	3.2	135	2.92
3GKB	B	48-51	DSSV	-70.1	-13.5	3.1	124	-78.6	-7.7	3.2	130	3.11
		60-63	DPEF	-66.4	-17.3	3	124	-117.2	-30.4	3.7	113	3.85
		69-72	DMRI	-70.4	-15.6	3.1	126	-98.2	12	3.6	142	3.05
3ALU	A	40-43	SCSG	-66.4	-26	3	113	-78	1	3.3	136	2.92
		92-95	TNSD	-72.5	-19.9	3.1	120	-75.8	-10.9	3.2	125	2.96
		98-101	WTDG	-60.8	-24	3	116	-83.5	-0.1	3.4	134	2.88
		138-141	YSNN	-69.6	-14.9	3.1	128	-83.5	-7	3.3	128	3.1
1ET7	A	88-91	PETN	-66.1	-14.4	3.1	125	-86.4	-7.7	3.4	129	3.04
		99-102	FHAA	-67.1	-17.7	3	120	-74.9	-18.1	3.2	120	3.07
		158-161	PREG	-63.1	-30.6	2.9	110	-91.7	16.6	3.5	145	3.07
		188-191	DENG	-60.3	-8.6	3	132	-107.2	5.3	3.7	135	2.91
		328-331	DDLM	-69.5	-42.7	3	99	-70.4	-37.4	3.1	107	3.62
2AAX	A	755-758	DSSK	-70.6	-8.4	3.1	129	-78.2	-17.6	3.2	121	3.29
		830-833	APDL	-59.1	-30	2.8	111	-97.7	6.6	3.5	138	2.93
		882-885	PKDG	-51.9	-37.8	2.9	108	-79.8	-20.2	3.3	122	2.76
3E7M	B	105-108	KSKS	-66.5	-29.7	3.2	115	-115.2	13.4	3.8	137	3.84
		110-113	LGSI	-53	-37.7	3	114	-90.5	19	3.4	146	3.27
		208-211	ARNC	-69.3	-9.5	3.1	128	-99.3	3.7	3.6	135	3.11
		266-269	MPDG	-56.4	-39.9	3	106	-77.6	10.4	3.2	144	3
		379-382	DTQR	-63.8	-29.3	3	113	-92.7	12.8	3.5	143	3.09

type I’ β-turn

PDB_ID	Chain	Residue No.	Sequence	i+1		*d* (Å)	*θ* (°)	i+2		*d* (Å)	*θ* (°)	C=O$_i$···N$_{i+3}$ (Å)
				φ (°)	*ψ* (°)			*φ* (°)	*ψ* (°)			
1A1X	A	94-97	VRGV	55.2	33.8	2.9	110	87.4	-1.6	3.4	136	3
1A68	A	72-75	VSGL	50.3	40.4	2.8	107	99.5	-19	3.6	144	2.99
		127-130	SGGR	78.8	27.3	3.2	111	122.8	5.4	3.9	124	4.47
1AE9	A	225-228	VDGY	47.2	53.9	2.8	98	71.6	19.2	3.2	120	3.02
1AJK	A	41-44	TNGV	54.9	40.1	2.8	105	81.5	-3.3	3.3	133	2.9
		77-80	VDGV	51.2	45.2	2.8	102	79.6	2.8	3.3	135	3.02
1AOK	B	78-81	KKGN	51.7	40.2	2.8	108	83.7	-3.9	3.4	137	2.79
1AYO	A	75-78	SNNH	45.8	41.3	2.8	107	62.1	30.4	3.2	119	3.02
1B5F	A	09-12	DRDT	53	32.1	2.9	114	59.6	7.5	3.1	134	3.02
1B5F	B	262-265	IGGK	58.5	30.3	2.8	110	77.2	-3.1	3.2	138	2.95
1B9K	A	807-810	YGGT	55.7	35.9	2.8	108	83	-0.8	3.4	137	2.84
1B9W	A	37-40	DAGK	47.5	52.1	2.8	107	87.6	-2.2	3.3	134	3
1BAM	A	101-104	ENSE	54.7	52	2.9	92	59.2	25.1	3	121	3.18
1BBP	A	63-66	IHGK	37.7	54.6	2.8	104	91.4	0.3	3.4	124	2.99
		94-97	YGGV	66.9	26	3.1	113	73.9	15.1	3.1	135	2.97
1BF2	A	53-56	GSGV	63	27.3	3	114	80.4	2.2	3.3	131	3.15
		545-548	DQGM	65.1	24.8	3.2	122	91.3	2.3	3.5	132	3.26
		588-591	LQCN	54.5	38.6	2.9	110	78.4	10.9	3.4	126	3.14
1BM8	A	10-13	YSGV	52.9	37	2.8	107	76.3	6.2	3.2	132	2.82
1BQB	A	106-109	YGGQ	59.8	23	3	119	94.5	-8.1	3.5	140	3.04
1BSL	A	97-100	SEGR	64.1	53.4	3.1	96	81.9	18	3.4	121	3.74
1BTE	A	48-51	ISGS	52.2	37.7	2.8	104	92.8	-16.4	3.4	144	2.94
1BTN	A	31-34	NNQE	51.5	50.9	2.9	102	56.2	26.3	3.1	118	3.16
1BYR	A	100-103	VDNV	52.5	34.1	2.8	113	56.5	12.9	3	130	3.18
1C2A	A	42-45	SMGD	65.3	18	3.1	124	80.8	1.3	3.3	135	3.16
1C30	A	174-177	MGGS	58.8	18.6	2.8	121	72.6	8.5	3.2	129	2.85
		340-343	TGGR	76.3	21.2			73.3	2.5	3.1	132	3.29
		532-535	CAAE	50.2	42.3	2.7	105	71.7	6.9	3.3	133	2.96
		833-836	KNNE	54	47.4	2.8	101	64.7	23.8	3	120	3
1C7T	A	827-830	AGGK	56.2	24.3	2.9	116	73	28.6	3.1	112	2.94
		847-850	DGGK	74.4	34.5	3.1	105	72.3	15.1	3.2	126	3.81
1C8U	A	96-99	QNGK	65.6	40.6	3	103	62.9	15.4	3	126	3.35
		255-258	SSAR	47.4	41.9	2.8	108	66	24.5	3.1	117	2.79
1C8Z	A	442-445	FHGR	49.1	46.7	2.8	101	83.3	-5.8	3.4	139	2.9
1CB8	A	522-525	HDAI	49.9	43.8	2.8	106	67.2	12.8	3.1	126	2.79
		632-635	VAGI	43	47.7	2.8	104	80.8	2.1	3.2	131	2.83
1CCW	B	387-390	GKGD	47	45	2.9	102	100.3	-15.1	3.6	144	3
1CCZ	A	39-42	ENSE	55.1	41.3	2.8	101	66.8	15.3	3.2	125	2.96
1CEM	A	76-79	AGGY	60.8	27	3.1	119	105.7	-6.1	3.7	137	3.12
		251-254	NNGT	57.3	37.1	2.8	108	67.8	14	3.1	128	3.09
1CFB	A	639-642	DNRS	47.7	43.4	2.9	101	75.1	11.9	3.4	128	3.21
1CHM	A	61-64	SFGR	50.9	34.1	2.8	114	74	17.5	3.3	127	2.82
		254-257	IAGY	50.2	38.6	2.9	116	85.9	-7.6	3.5	133	2.99

1CI9	A	44-47	RHGE	47	44.4	2.8	106	90.5	-4	3.3	137	2.83
1CJW	A	86-89	VEGR	54.2	46.6	2.8	98	74.1	10.9	3.3	130	3.09
1CP2	A	48-51	LGGL	81	59.8	3.4	134	81.4	-12	3.2	141	4.25
1CPN	A	66-69	TNGV	61.4	42.2	2.7	103	78.1	-3	3.1	138	2.84
		102-105	VDGV	49.5	39.8	2.7	104	80.8	-1.1	3.2	140	2.77
1CQY	A	447-450	GSWD	56.3	50.1	2.9	97	63.5	18.5	3.1	125	3.21
1CV8	A	113-116	RNGM	46.4	48.7	2.7	103	70.6	3.3	3.2	135	2.68
1CVR	A	265-268	KDGK	46.3	51.8	2.7	98	73.9	5	3.3	132	2.91
		384-387	DDGD	41.5	57.4	2.7	95	80.3	3.7	3.3	131	2.78
		395-398	KDGK	49.1	39	2.8	108	83	1.9	3.3	133	2.89
1CXY	A	28-31	IHGK	52.3	41.3	2.8	105	72.2	9.1	3.2	127	2.99
		63-66	TKSY	60.4	17.7	2.9	121	66.6	9.8	3.2	137	3.13
1CY9	A	241-244	QNDK	61.4	45.3	2.9	99	58.2	13.3	3	129	3.19
1CYQ	A	32-35	GGGL	62.3	30.7	2.9	111	65.4	11	3.1	128	3.13
		68-71	INQV	54.7	50.3	2.8	96	73.1	-6.1	3.2	142	3.13
		89-92	INGK	55.5	35	2.9	110	69.1	17.2	3.1	122	2.8
1D2I	A	08-11	YNHA	48.5	40.5	2.8	104	61.1	34.4	3	111	3.22
1D2O	A	565-568	ANGE	57.8	41.9	2.9	100	79.6	1.3	3.3	134	3.15
		670-673	NNWT	52	30.7	2.9	115	58.7	27.8	3	117	2.88
		683-686	AKGQ	54.1	54.3	2.9	94	78.9	-15.6	3.4	148	3.26
1D5T	A	112-115	KGGK	58.3	39.4	2.8	112	99.7	-6.4	3.5	132	3.32
		158-161	FEGY	48.7	43.5	2.8	106	82	-2	3.3	137	3.02
		261-264	ENGK	48.6	40.7	2.8	106	78	10.2	3.3	129	2.97
		270-273	SEGE	38.8	52.1	2.9	104	83.2	10.9	3.3	126	3.05
		311-314	TNDA	50.2	43.3	2.8	103	60.4	32.8	3	115	3.22
1D7W	A	53-56	RNGF	49.3	47.7	2.8	97	93.9	-9.9	3.5	139	2.79
1D8C	A	194-197	VDKQ	54.9	47.5	2.8	100	68.3	16.9	3.4	124	2.98
		233-236	NNGL	48.6	31	2.8	115	83.8	-5.5	3.4	140	2.72
1DC1	A	191-194	INGK	50.4	42.5	2.9	109	87.7	-23.9	3.4	152	2.96
1DEX	A	17-20	GSGT	38.3	54.5	2.8	101	83.7	-1.5	3.4	132	3.08
		100-103	YDGV	56.5	42	2.9	102	74.6	13.2	3.2	126	3.26
1DFX	A	28-31	CCGE	53.9	41.1	2.9	105	68.2	3.5	3.3	139	3.05
1DG6	A	170-173	RNGE	53.6	41.5	2.8	106	81.8	-0.8	3.3	136	3.06
1DGF	A	396-399	DNQG	55.1	35.7	2.9	110	65.2	23.4	3.2	122	2.86
1DQP	A	183-186	GFGL	51.4	37.7	2.8	109	93.3	-19.6	3.6	147	2.84
		188-191	DNGL	53	47.2	2.8	97	91.4	-24.7	3.5	152	3
1DUS	A	21-24	LRGK	51.4	34.2	2.8	110	74.6	9.1	3.3	129	2.65
		186-189	KGGY	47.8	44.4	2.8	105	83.2	1.9	3.3	132	2.92
1DUW	A	71-74	GNFV	66.3	27	3	111	65.8	15.9	3.1	126	2.99
1DXJ	A	26-29	GKGF	54.5	49	2.8	98	94.1	2.3	3.5	129	3.19
1E6Y	B	2103-2106	VKGG	56	51.7	2.9	98	60.7	29.4	2.9	113	3.14
		2201-2204	SNRN	59.5	39.1	3	106	60.2	32.1	3	113	3.19
1E6Z	A	303-306	NGGQ	57.6	32	2.9	112	91.8	-13.5	3.4	142	3.07
		470-473	YQGY	55.5	30.2	3	117	88.2	14.8	3.4	122	3.23
1EI5	A	31-34	KDGE	55.3	33.2	2.9	108	81.1	-0.6	3.3	137	3.02
		271-274	GFGL	39.6	52.5	2.7	106	78.4	14.3	3.2	126	2.91
		279-282	TGGK	54.3	31.9	2.8	110	82.2	2.6	3.3	133	2.88

		444-447	EGGA	75.3	22.7	3.1	113	98.3	-12.5	3.5	141	3.4
1EIF	A	23-26	IDGV	53.8	41.7	2.8	103	79.3	-4.3	3.3	140	2.96
		122-125	AVGQ	46	49.7	2.8	101	80.4	3.4	3.3	131	2.98
1EQC	A	227-230	DAFQ	58.6	29.6	3	119	63	26.8	3.1	118	3.03
1ERZ	A	101-104	EGGV	54.3	37.1	2.8	107	83.2	5.3	3.3	130	2.85
		161-164	VDDA	49.8	51.2	2.8	98	71.9	17.9	3.2	121	3.05
		239-242	EENC	49.9	47.4	2.8	100	69.8	13.4	3.2	127	2.94
1ETO	A	70-73	TLGN	56.8	38.3	2.9	108	71.7	14.9	3.2	125	3.02
2BWR	A	146-149	GGGQ	53.5	34.2	2.9	112	74.2	11.4	3.2	126	2.97
2I4B	A	134-137	TNGN	54.6	39.5	2.9	107	90.2	-2	3.5	134	3.13
		338-341	GDGQ	54	39.6	2.9	107	69	6.7	3.1	132	2.98
3V5C	A	52-55	IDGQ	45.9	47.5	2.7	100	71.3	11.9	3.2	129	3.03
		208-211	ANNA	43.1	44.5	2.7	104	65.2	16.8	3.2	128	2.97
		353-356	ENGE	50.5	42.5	2.8	106	78.1	21.1	3.2	118	3.06
2P02	A	213-216	DRGA	56	44.1	2.9	102	89.2	-24.6	3.5	150	2.94
		293-296	YGGW	68.9	25.6	3	116	102.7	-23.3	3.6	148	3.17
		356-359	HYGT	60.1	18.8	3	127	78.1	16.8	3.3	126	2.85
3U7E	B	273-276	NEGI	50.8	33.5	2.8	116	74.1	9.6	3.2	128	3.02
1UW8	A	296-299	SDGH	53.7	40.3	2.8	105	69.3	16.1	3.1	123	2.86
1YU2	A	85-88	VNGK	53.7	39.1	2.9	108	85.5	-0.6	3.4	136	2.96
		185-188	VAGA	53.5	25.7	2.9	120	72.4	7	3.2	133	2.89
		326-329	TGGR	73.9	32.3	3.2	110	84.7	23.5	3.4	117	3.92
2ZWV	A	193-196	ILGA	53.4	41.4	2.8	105	76.8	13.5	3.2	124	2.99
3IKW	A	32-35	IDGC	59.5	26.9	2.9	118	68.2	6.7	3.1	132	3.08
		55-58	FDGK	52.6	42.3	2.8	101	74.2	0.9	3.3	138	2.97
		234-237	SGGL	56	31.4	2.9	117	83.6	14.2	3.3	123	2.94
1CA1	A	347-350	ANGK	50.4	41.9	2.9	109	78.7	18.4	3.3	122	2.95
2W5N	A	78-81	DGGK	74.7	20.8	3.1	119	103	1.9	3.7	130	3.55
		142-145	DKGY	61.3	29.7	3	112	112.7	11	3.7	128	3.78
		319-322	ALGD	45.2	34.7	2.9	117	92.2	16.1	3.6	123	3.09
		362-365	AGGK	65	17.3	3	122	78.8	27.3	3.2	115	3.41
1J1B	B	532-535	KDGS	70.3	15.4	3.1	124	86.1	-3.3	3.3	137	3.04
3RHG	A	154-157	IDGT	57.3	37.9	2.9	110	83.3	0.1	3.4	136	2.94
1K9Y	A	164-167	VDGV	53.1	38	2.8	108	79.2	4.6	3.3	135	3.12
2G8S	A	146-149	ENNQ	61.2	16.2	3	126	71.5	24.2	3.3	119	2.91

type II β-turn

PDB_ID	Chain	Residue No.	Sequence	i+1		d (Å)	θ (°)	i+2		d (Å)	θ (°)	C=O$_i$⋯N$_{i+3}$ (Å)
				φ (°)	ψ (°)			φ (°)	ψ (°)			
1C0I	A	1124-1127	PPGA	-70.1	132.7	2.9	110	80.3	-10	3.3	143	3.3
		1169-1172	FDGA	-82.3	89.4	3.6	141	61.3	21.6	2.9	119	4.21
		1186-1189	IAGI	-61.2	129.9	2.9	107	105.8	-14.1	3.2	141	2.91
		1227-1230	RPGG	-50.1	135.8	2.8	94	114	9.8	3.8	125	3.74
		1239-1242	GVGD	-68.6	130.7	3	107	75.3	9.2	3.1	128	3.06
		1288-1291	RRGG	-61.4	128.2	2.8	110	96.3	2.7	3.7	132	2.77
1C39	A	11-14	EKGK	-48.5	121.1	2.7	102	108.5	-24.1	3.7	147	2.74
		50-53	RVCR	-92.6	111.2	3.5	135	58.2	24.8	3.1	123	4.49
1A12	A	104-107	VEGS	-50.6	128	2.8	102	83	-7.6	3.4	142	3.02
		157-160	EPMK	-47.9	130	2.8	97	65.5	10.8	3.1	133	3.24
		329-332	GEGA	-56.8	145.1	2.9	92	70.9	11.8	3.2	125	3.37
		396-399	LENR	-58.5	127.4	2.9	108	63.1	26.2	3	115	3.17
1A1X	A	18-21	QEGI	-49	119.6	2.7	103	84.4	-8.3	3.4	139	2.8
		73-76	YPEE	-54.9	149.7	2.9	87	76.9	37.6	3.3	107	3.8
1A27	A	55-58	PPGS	-49.3	137.7	2.6	90	87.9	-15.8	3.4	146	2.85
		149-152	LPFN	-63.4	138.9	3	98	69.4	15.7	3.2	133	3.14
1C8U	A	18-21	EEGL	-60.1	122.3	2.9	112	65.6	24.7	3	116	2.89
		221-224	EPGI	-63.6	132.1	3	106	83.1	-4.4	3.3	137	3.27
1C8X	A	195-198	YYGT	-50.8	135.8	2.9	97	70.2	20.3	3.2	122	3.22
		217-220	EIGR	-60	117.5	2.9	109	102	-24.5	3.6	147	3.13
1A9X	B	1548-1551	YSRQ	-50.4	130.9	2.8	96	61.4	25.7	3.1	121	2.98
		1653-1656	LNGM	-66.6	122.6	2.8	115	77.1	11.2	3.4	130	2.99
1AA2	A	16-19	TAGY	-60.7	135.5	2.8	94	81.1	-8.4	3.4	142	2.99
		19-22	YPNV	-62	145.5	2.9	94	59.8	23.3	2.9	123	3.34
1ABA	A	39-42	EKGV	-51.1	119.6	2.8	97	72.8	8.7	3.2	132	2.91
		60-63	QIGL	-56.9	122.9	2.9	105	77.5	0.3	3.3	134	3.03
1AE3	A	50-53	DEGQ	-52.2	132.6	2.9	94	77.7	-1.5	3.3	139	3.24
1AJK	A	132-135	QTGG	-52.7	133.2	2.8	98	81.7	5.6	3.4	130	2.92
1AMX	A	247-250	FPGS	-46.4	125.6	2.8	98	108.3	-27.5	3.7	150	2.84
		270-273	GSYN	-64	130.7	3	109	68.3	20.1	3.2	125	3.56
		301-304	EHGK	-57.8	121.2	3	109	77.1	4.9	3.3	132	3.18
1AOC	A	08-Nov	CLCD	-58.3	137.1	2.8	96	67.3	3.2	3.2	137	2.99
		42-45	GVSG	-61.3	145	3	94	71.8	37.3	3.2	108	3.51
1AOK	B	15-18	GAFS	-48.7	148.3	2.8	85	72.8	-4.4	3.3	145	3.12
1AT0	A	279-282	SIGD	-59.5	133.6	2.8	99	109.3	-30.9	3.7	151	3.04
		350-353	EEKN	-55.7	150.7	2.8	86	84.8	-26.8	3.4	156	3.16
1ATG	A	71-74	LPGS	-52.9	135.8	2.8	92	82	-2.5	3.3	137	3.13
		89-92	KPGL	-60.5	144.5	2.9	92	79.2	-12.3	3.3	144	3.37
1AYO	A	51-54	VSGF	-54.3	131.3	2.8	100	91.5	-12.3	3.5	143	3.03
1B12	A	96-99	LIGD	-57.9	130.9	2.8	103	102.6	-16.9	3.4	142	2.88
		126-129	KRGD	-57.8	133.9	2.8	101	90.8	-11.9	3.5	142	2.99
		150-153	LPGD	-43.9	126.3	2.7	96	95.8	-19.3	3.5	145	2.88
		167-170	QPGC	-56	146.8	3	88	52.1	28.2	2.9	118	3.49

		253-256	QPGQ	-51.4	132.1	2.7	97	85.7	-6.3	3.4	139	2.91
		257-260	QLAT	-60.7	144.6	2.9	92	74.7	13.1	3.3	126	3.45
		264-267	PPGQ	-59.7	143.5	2.8	94	62.7	19.6	2.9	120	3.05
1B1C	A	68-71	IDNA	-66.2	139.1	3	103	53.6	34.7	2.9	110	3.41
		103-106	LSGV	-60.5	138.8	2.9	95	95.7	-17.8	3.5	144	3.15
		120-123	HFNA	-67.9	115.8	3.1	122	55.2	32.7	2.9	114	3.56
1B5F	A	166-169	VFGG	-93.6	127.3	3.5	121	85.2	25.4	3.3	113	4.39
1B66	A	122-125	PVGA	-58.8	138	2.9	98	76.8	-2.3	3.3	136	3.19
1B97	A	98-101	KENE	-57	128.5	2.9	106	75.5	19.9	3.3	124	3.3
1B9K	A	778-781	DGGA	-63	138.5	2.9	98	76.3	9	3.2	136	3.25
1B9W	A	13-16	PENA	-60.9	142.3	2.9	87	64.2	30.3	3.1	116	3.28
		31-34	LLYF	-55.7	149.2	2.9	95	68.9	22.4	3.1	127	3.28
		76-79	KEGS	-63.7	139.1	2.9	101	71	9.2	3.1	122	3.02
1BAM	A	51-54	EKNC	-66.2	145.3	3	91	58.1	25.6	3.1	124	3.55
		169-172	TEGQ	-52.9	140.4	2.8	91	100.7	-13.3	3.5	140	3
1BBP	A	38-41	EKYG	-42.4	125.6	2.9	99	74.9	-5	3.3	137	3.02
1BF2	A	93-96	GKGS	-50.6	121.6	2.8	106	86.8	14.9	3.4	123	2.86
		97-100	QAGF	-52.3	118.9	2.9	109	101.8	-13.7	3.7	142	3.09
		200-203	YRGT	-57.5	131.4	2.9	101	72.6	25.2	3.2	118	3.29
		267-270	AAGG	-58.8	124.3	2.8	108	89.4	-9.6	3.5	142	2.76
		329-332	TSGN	-57.1	130.8	2.8	99	96.1	-14.6	3.5	141	2.77
		445-448	QLGG	-63	114.9	2.9	115	95.1	3.5	3.5	135	2.78
		450-453	PQGW	-62.3	141.1	2.9	100	107	-13.4	3.7	141	3.18
		470-473	ELGS	-60.4	127.4	2.8	103	93.9	-31.1	3.5	156	2.93
		579-582	QGGD	-55.3	130.2	2.9	103	110.2	-24	3.7	147	3.01
1BG6	A	100-103	SEGQ	-57.3	135.8	2.8	96	105.5	-15.4	3.8	140	3.26
		111-114	ATGG	-97.9	113.2	3.4	134	84.7	22.9	3.5	117	4.28
		146-149	RPGQ	-55.6	126.2	2.9	109	101.9	-1.4	3.2	132	2.87
		280-283	YRGI	-67.4	112	3.1	123	101.4	0.5	3.6	129	3.32
		346-349	LSGL	-54.7	128.3	3	102	73.4	15.5	3.3	125	3.18
1BHD	A	167-170	YSQV	-78	137.1	3.1	108	76.7	17.1	3.2	123	3.49
1BHE	A	37-40	DQGK	-53.9	133.3	2.9	96	83.8	2.7	3.4	134	3.12
		60-63	PSGV	-62.4	133.5	3	104	79.5	-3.3	3.3	136	3.22
		68-71	DKGV	-52.5	137.1	2.8	93	76.5	-2.3	3.2	134	2.96
		85-88	APSS	-46.8	113.4	2.8	110	62.1	25.9	3	120	2.96
		231-234	YKGR	-62.4	132.9	2.9	102	83.9	5	3.3	129	3.15
		345-348	GENA	-56.5	143.3	2.8	89	79.5	-6.4	3.3	142	3.16
1BHT	A	77-80	NKGL	-79	118	3.2	122	68.7	29.6	3.2	117	3.83
		105-108	SSGV	-85.6	147.8	3.3	104	85.5	22.6	3.3	121	3.94
		167-170	YRGK	-64.1	141.2	3	98	87.3	-10.2	3.4	144	3
		183-186	EEGG	-62.4	156	2.9	86	97.4	-11.4	3.4	138	3.46
1BOl	A	34-37	APGY	-53.9	131	2.9	103	98.3	-6.9	3.6	137	3.1
		125-128	EEGE	-42.4	128	2.9	92	91.9	-0.3	3.4	132	3.06
1BQB	A	22-25	IDGG	-60.2	106.5	3	121	67.7	21	3.1	120	3.19
		191-194	TPGK	-54.8	135.8	2.9	95	93.9	-25.2	3.4	151	2.99
1BS9	A	12-15	ETTA	-58.9	131.8	2.9	101	71.4	-2.9	3.5	146	3.16
		33-36	YPGS	-54.4	139	2.8	93	96.3	-19.9	3.6	146	2.98

		137-140	RAGL	-56.2	142	2.8	91	108.1	7.1	3.7	125	3.82
		156-159	PAGF	-56.2	136.6	2.9	98	96	-7.3	3.4	136	3.19
1BSL	A	164-167	TEGG	-64.5	117.7	2.8	119	100.5	1.3	3.7	132	2.92
1BTE	A	88-91	GNMC	-53.7	122	2.9	99	69.8	18	3	125	2.95
1BTN	A	58-61	LKEA	-60.2	118.2	3	112	60.9	36.4	3	110	3.17
1BXU	A	22-25	QAGD	-51.5	132	2.7	98	99	-11.6	3.5	143	2.96
		41-44	VEGQ	-47.5	126	2.8	100	74.7	-2.4	3.3	135	2.96
		65-68	SPGE	-61.2	135.8	2.9	99	93.6	-12.1	3.5	140	3.25
1BYF	A	83-86	SPNE	-56	132.5	2.9	100	65.9	26.8	3.1	118	3.16
1C8Z	A	259-262	PQGI	-56.8	138.2	2.7	95	93.9	-13.5	3.5	142	2.94
1YIX	A	86-89	EEGV	-60.7	146.7	2.8	92	100	-32.4	3.5	154	3.12
		214-217	HRGK	-59	142.6	2.8	94	93.6	-8.1	3.5	138	3.22
2C1D	A	116-119	MAGL	-53.8	138.5	2.8	93	83.8	-23.7	3.4	154	3.17
		171-174	SRGM	-57.2	133.3	2.8	100	87.4	-16.8	3.5	145	2.94
		213-216	NAGN	-58.1	130.9	2.9	100	100.2	-18.8	3.6	147	3.01
		261-264	KAGS	-54.5	127.8	2.9	106	82.5	1.6	3.4	136	2.96
		278-281	GNGL	-53.1	125.4	2.9	104	108.7	-34	3.8	159	3
2JF2	A	15-18	EEGA	-55.3	131	2.8	98	111.8	-21.3	3.8	145	3.1
		21-24	GANA	-59.3	137.7	3	97	56.3	19.2	3	125	3.29
		27-30	GPFC	-56.8	141.4	2.9	92	63	22.1	3.1	122	3.43
		39-42	GEGT	-51.8	134	2.9	95	77.8	-6	3.2	142	3.19
		63-66	YQFA	-51	142	2.9	88	72.3	8.3	3.2	133	3.43
		93-96	RESV	-51.6	137	2.9	91	65.5	10.9	3.1	131	3.24
		118-121	MINA	-48.4	130.6	2.8	93	61.2	19.1	3.1	125	3.01
		124-127	AHDC	-43.3	132.6	2.8	93	73.6	13	3.3	129	3.42
		136-139	ANNA	-50.5	133.2	2.8	95	61.1	12.9	3.1	129	3.09
		142-145	AGHV	-57.9	142.3	2.9	95	69.4	18	3.1	122	3.58
		148-151	DDFA	-50	136.7	2.9	92	73.4	-3.3	3.3	144	3.24
		154-157	GGMT	-65.6	142.9	3	96	64.4	25.5	3.1	118	3.37
		160-163	HQFC	-56	141	2.9	92	77.6	-8.9	3.4	146	3.29
		166-169	GAHV	-58.3	139.9	3	94	64.7	14.5	3.1	129	3.38
		182-185	PPYV	-63	152.4	2.9	89	76.9	6.9	3.3	134	3.62
3B1V	A	31-34	WPGV	-74.7	134.9	3.2	112	102.5	33.3	3.7	111	4.16
1SKF	A	78-82	IVGD	-62.2	134.6	2.9	102	107.5	-29	3.7	153	3.04
		206-209	YSGA	-64.5	132.4	3	107	95.1	-11.4	3.5	142	3.07

type II’ β-turn

PDB_ID	Chain	Residue No.	Sequence	i+1		d (Å)	θ (°)	i+2		d (Å)	θ (°)	C=O$_i$···N$_{i+3}$ (Å)
				φ (°)	ψ (°)			φ (°)	ψ (°)			
1AJK	A	158-161	GGVF	66.3	-134.3	2.9	103	-63.2	-22.7	2.8	122	3.09
1AYE	A	277-280	YGFL	63.3	-126.7	3	107	-71.4	-21.3	3.1	119	3.08
1B12	A	230-233	LGDV	65.6	-109.5	2.9	126	-115.7	17.3	3.8	141	2.94
1B5F	A	91-94	IGDL	57.6	-121	2.8	113	-108.6	6	3.7	137	2.82
		199-202	IGDK	58.7	-120.6	2.8	115	-105.7	6.8	3.6	135	2.8
1B9K	A	731-734	RQNL	57.6	-126.9	2.9	96	-101.9	20.1	3.5	150	2.97
1BHT	A	130-133	IGKG	71.1	-117.5	3.1	123	-93.2	-11.4	3.6	125	3.49
1C7K	A	104-107	SGGG	61.1	-131.1	2.8	103	-81.6	-1.9	3.3	132	2.96
1C7T	A	109-112	TGDL	56.7	-132.5	2.9	99	-90.4	6.9	3.4	142	3.17
		178-181	TGDQ	46	-125	2.8	100	-109.4	29.5	3.6	151	3.05
1CB8	A	277-280	RGSY	73.5	-113.9	3.1	125	-113	5.4	3.8	134	3.08
1CCW	B	218-221	PLTG	53.8	-120.1	2.9	110	-78.1	-0.7	3.2	134	2.92
		433-436	FGNV	52.8	-134	2.8	99	-99.8	15.6	3.6	144	2.98
1CCZ	A	30-33	KQKD	74.1	-116.9	3	122	-112.6	3	3.7	131	3.21
		126-129	QGKE	71.9	-137.9	3	105	-99.2	26.2	3.5	151	3.21
1CHM	A	83-86	DGGQ	39.4	-121.3	2.9	102	-84.5	-9.7	3.5	132	3.29
1CJW	A	183-186	VGSL	60.8	-129.8	2.9	105	-100.5	8.1	3.6	138	2.97
1CPN	A	181-184	GGVF	70.8	-133.3	2.8	113	-86.8	4.1	3.2	136	3.01
1CT9	A	109-112	KGPE	57.8	-139	2.8	93	-78	2.6	3.3	139	2.94
1CY9	A	417-420	AGDF	62.5	-134.6	2.9	103	-86.2	20.4	3.3	150	2.85
1D02	A	170-173	YDHY	49.4	-121.7	2.7	102	-79.7	10.7	3.2	142	2.83
1D0Q	A	02-05	GHRI	42.3	-147.7	2.7	80	-50.8	-7.9	3.3	147	3.31
		32-35	QGRN	63.9	-115.4	2.9	114	-98.9	8.1	3.6	134	3.17
1D3Y	A	170-173	LGTG	65.1	-130.6	3	106	-66.1	-32.8	3	111	3.27
1D5T	A	40-43	GGES	54.4	-131.5	2.8	104	-68.8	-29.8	3	112	2.82
1D7B	A	76-79	NGNQ	72.5	-131.2	3.1	115	-100.3	23.8	3.6	151	3.4
1D8C	A	07-10	QSRL	45	-130.2	2.5	92	-98.1	21.4	3.5	146	2.53
1DEX	A	14-17	NGGG	43.3	-122.7	2.8	100	-105.2	19.9	3.7	142	2.94
1DGF	A	398-401	QGGA	51.5	-132.8	3	100	-98.8	16.5	3.6	143	3.31
1DO6	A	25-28	EGEL	77.1	-122.7	3.1	121	-96.4	1.4	3.5	134	3.15
1DTD	A	633-636	YGFL	62.4	-132.7	3	107	-68.7	-22.7	3.1	118	3.16
1DUS	A	64-67	CGYG	57.2	-138.9	2.7	98	-81.2	9.6	3.2	140	2.92
		113-116	DLYE	54.3	-122.9	2.9	108	-93.9	-2.3	3.4	129	3.07
1DVO	A	89-92	DGDT	70.3	-128	3	113	-94.1	-0.5	3.5	131	2.95
1DXJ	A	161-164	QGNK	54.1	-122.3	2.9	111	-78.8	-5.3	3.3	130	2.89
1E1H	B	387-390	DGFN	60.7	-140.6	2.9	101	-75.3	-17.7	3.2	122	3.08
1E6Y	B	2158-2161	WGSY	67	-130.6	3	108	-93.2	1.5	3.5	135	3.47
1E8C	A	415-418	PRTE	49.8	-127.6	2.7	100	-90.7	3.9	3.5	135	3.05
		465-468	KGHE	59.4	-113.5	2.8	118	-83.5	-18.4	3.4	123	2.97
		473-476	VGNQ	74.3	-124.7	3	117	-114.6	14.2	3.8	140	2.98
1EAR	A	106-109	EDDE	62.9	-109.5	3	123	-115.1	20.2	3.8	143	3.09
1EF1	A	251-254	NDKK	58.9	-116.5	3	116	-83.4	-16.2	3.3	122	3
1EG3	A	58-61	QGPW	74.2	-126.6	3.1	109	-84.2	-3.1	3.4	136	3.25

1EI5	A	398-401	DGET	64.9	-122.6	2.9	117	-96	20	3.5	147	3.21
1EIZ	A	113-116	VGDS	59.6	-138.7	2.9	98	-91	6.7	3.4	139	3.16
1EQC	A	40-43	NGND	62.1	-118.3	2.9	115	-100.1	0.5	3.6	134	2.87
		157-160	NGDN	56.1	-135	2.9	96	-98.7	11.5	3.6	142	3.17
1ES6	A	83-86	SGPK	62.4	-127.2	2.9	108	-80.1	-6	3.3	131	2.99
1EU1	A	505-508	LYEA	50.5	-133.4	2.8	95	-101.5	16.4	3.7	146	3.01
		648-651	LHSQ	59.5	-120.1	2.9	111	-86.8	-14.2	3.5	126	2.8
1F1E	A	18-21	IGER	52.5	-133.7	2.8	100	-90	4.6	3.4	135	3.16
1F3U	A	35-38	SGRG	56.3	-123.1	2.8	108	-68.5	-14.6	3.1	126	2.97
1FAO	A	189-192	HRNE	55.7	-126.8	2.8	105	-109.1	18.2	3.7	142	2.89
1FBW	A	206-209	YGFL	67.1	-132.3	3	106	-110.1	-1.2	3.7	128	3.45
1FF3	A	126-129	QGND	63.8	-124.9	2.9	111	-99.8	11.9	3.6	141	2.96
1FHU	A	36-39	EGER	65.3	-132	2.7	104	-86.1	-14.7	3.4	125	2.93
1FN9	A	30-33	EGWD	69	-142.2	3	100	-89.5	5.5	3.5	139	3.39
		45-48	CGGA	65.9	-128.4	3	111	-90.6	-2.7	3.4	130	3.06
1FNF	A	1220-1223	KDDK	58.2	-121.3	2.9	107	-86.9	4	3.4	136	2.94
		1311-1314	YEQH	63.8	-99	3	131	-108.7	-29.2	3.7	115	2.82
		1401-1404	NGRE	68.7	-137.6	2.9	101	-79.1	0.3	3.2	137	2.91
1FP2	A	197-200	GGTG	64.6	-132.9	3	106	-95.8	11	3.5	142	3.07
		342-345	TGFL	52.8	-123.1	2.9	108	-78.5	-7.1	3.3	127	2.9
1FS5	A	144-147	IAFN	58.6	-140.9	2.8	95	-107.1	36.1	3.6	154	3.04
1FZU	A	06-09	CGSN	68.4	-116.1	3	120	-96.8	-6.5	3.5	129	2.92
		96-99	SGNN	45.5	-125.2	2.8	98	-99.5	19.6	3.6	146	2.89
1G38	A	48-51	CAHG	61.1	-115.1	2.8	117	-86.6	0.7	3.3	133	2.78
		137-140	KGKY	60.4	-132.2	2.9	104	-80.6	-27.6	3.2	112	2.98
1G8K	A	557-560	PGTA	42.4	-123.8	2.7	98	-104.2	22.9	3.7	150	2.94
		707-710	WQTA	48.2	-126.7	2.9	97	-79.3	12.6	3.3	145	2.87
1GA8	A	120-123	LGDN	56.8	-133.6	2.8	100	-99.7	18.6	3.5	144	2.9
1GPQ	A	46-49	LGDE	70.5	-127.7	3	114	-112.1	27.6	3.7	150	3.05
1GR0	A	54-57	FGPY	66.8	-126	2.9	113	-87.7	-14.6	3.3	120	3
		85-88	ASEN	54.1	-126.8	2.9	102	-88.6	0.3	3.4	133	3.05
		108-111	TLDG	49.1	-126	2.7	101	-104.6	29.7	3.6	150	2.88
		280-283	LDDR	58.5	-132.8	2.9	101	-75.8	-4.6	3.2	133	3.06
1GT9	A	341-344	TGLG	60.1	-132.5	2.9	101	-73.8	-9.2	3.2	127	2.96
1GV3	A	183-186	PNQD	45.7	-116.8	2.8	106	-110.8	19.3	3.7	144	2.93
		210-213	YQNR	55	-123.6	2.9	105	-79.1	-8.5	3.3	129	2.95
1GX3	A	159-162	HGDD	79.6	-140.9	3.1	108	-89.7	3.4	3.4	136	3.12
		222-225	RGRN	61	-129.2	2.9	111	-80	-8.6	3.4	129	2.96
1GXN	A	327-330	TGRM	63.5	-124.5	3	114	-111.4	18.7	3.8	143	3.1
1H0B	A	52-55	NGNN	65.6	-129.2	3	110	-88.8	-0.8	3.5	134	3.28
1H0H	A	665-668	VGDV	62	-125.8	2.9	108	-98.7	10	3.6	140	2.91
		861-864	WQTG	58.2	-130.3	2.9	98	-69.7	-1.8	3	135	2.72
1H17	A	631-634	PGAN	49.4	-123.4	2.8	104	-95.8	12.1	3.6	142	2.88
1H6W	A	379-382	IGTR	53.3	-140	2.7	92	-98.1	24.8	3.6	147	3.1
1H74	A	18-21	LGVG	74.1	-111	3.1	129	-70.8	-1.4	3.1	136	3.77
		259-262	SGSG	57.5	-137.6	2.9	99	-88.6	9.8	3.3	139	2.86
1H7W	A	850-853	DGQS	67.3	-125.8	2.9	113	-90.8	4.1	3.4	136	3.03

1HE3	A	77-80	TRND	47.9	-116.8	2.9	107	-97.1	8.8	3.4	139	2.8
1HG8	A	116-119	KGSN	63.8	-125.6	2.9	112	-86.6	3.5	3.3	139	3.15
1HKK	A	327-330	RDNQ	55.7	-128.5	2.8	102	-94.4	18.8	3.5	146	2.97
1HN0	A	486-489	PGGK	57.5	-134.6	2.9	95	-102.5	18.8	3.6	146	3.16
		558-561	AGRH	55.9	-139.1	2.8	97	-81.7	6.7	3.3	140	3.13
		635-638	WQDK	53.3	-124	2.8	106	-96.2	7.7	3.4	138	2.91
		761-764	ADNH	52.3	-127.6	2.8	101	-83.1	8.5	3.3	143	3.07
		997-1000	SGDN	72.5	-125.4	3.2	113	-81.2	-14.4	3.3	122	3.24
1WA5	C	584-587	SEDS	52.1	-110.9	2.8	116	-104	18.4	3.6	142	2.86
2IVF	A	854-857	IHST	50.2	-130.8	2.8	97	-97.4	4	3.5	137	3.02
1CZA	A	172-175	TKRF	59.5	-132.2	2.9	107	-92.3	-10.5	3.5	128	3.44
		620-623	TKGF	58	-136.9	2.9	93	-101.3	10.6	3.5	134	3.53
4A7K	A	280-283	VGDD	62.4	-123.6	3	108	-69.7	-14.6	3.3	133	3.1
4ARC	A	330-333	YGTG	51.2	-134.1	2.9	97	-88.4	0.8	3.5	134	3.11
2WAN	A	137-140	DAAS	42.6	-112.6	2.8	103	-69.6	-21.8	3.2	121	2.83
		526-529	EGTA	60.4	-137.2	3	95	-101.3	7.4	3.6	138	3.75
		893-896	LGDQ	56.9	-126.6	2.9	104	-84.7	5.3	3.3	138	3.06
3UCP	A	559-562	NGSK	66.9	-118.5	3.1	115	-94	0.5	3.6	133	3.01
3K59	A	623-626	EGDK	63.4	-125.3	2.8	111	-94.3	-7.9	3.4	125	2.95
3VTH	A	417-420	KGHY	57	-131	2.9	101	-102.1	29.7	3.6	150	2.8
2E7Z	A	488-491	AGEA	54.6	-132	2.8	94	-108.4	28.8	3.7	150	3.01
		611-614	FQSC	53.6	-126.2	2.8	102	-91.5	13.1	3.5	143	2.76
4FNQ	A	16-19	AGKA	53.7	-122.1	2.8	108	-104.9	25.6	3.6	148	2.79
		339-342	TYFD	51.8	-119.5	2.7	110	-87.5	-26.6	3.5	114	2.72
		370-373	FGKR	46.7	-127.8	2.9	96	-110.1	24	3.7	149	3.13
2YA0	A	636-639	LGTA	50.7	-147.1	2.9	85	-82	-28.2	3.2	109	3.65
1Q8I	A	623-626	EGDK	63.8	-132.9	3	103	-90.8	3.9	3.4	137	3.03
1R1H	A	151-154	RGGE	52.6	-128.7	2.8	104	-104.7	6.5	3.7	136	3
1Z45	A	452-455	NGVN	58.3	-125.9	2.9	107	-98.4	6.4	3.5	140	2.76
1KMO	A	371-374	QGKR	65.3	-123.1	2.9	115	-94.9	14.4	3.5	144	2.94
		397-400	IGPS	75.3	-135.1	3.1	109	-80.2	-16.9	3.4	122	3.51
		457-460	IGNW	61	-131.3	2.8	105	-88.4	5.6	3.4	139	2.85
		644-647	PGNW	49.8	-118	2.8	110	-98.2	2.3	3.6	135	2.92
1KWG	A	561-564	EGRY	70.4	-130.1	3	110	-92.6	4.3	3.4	134	3.23
		603-606	RGTW	66.7	-134.9	2.9	105	-87.7	4.3	3.4	138	2.99

Figure S1.

A.

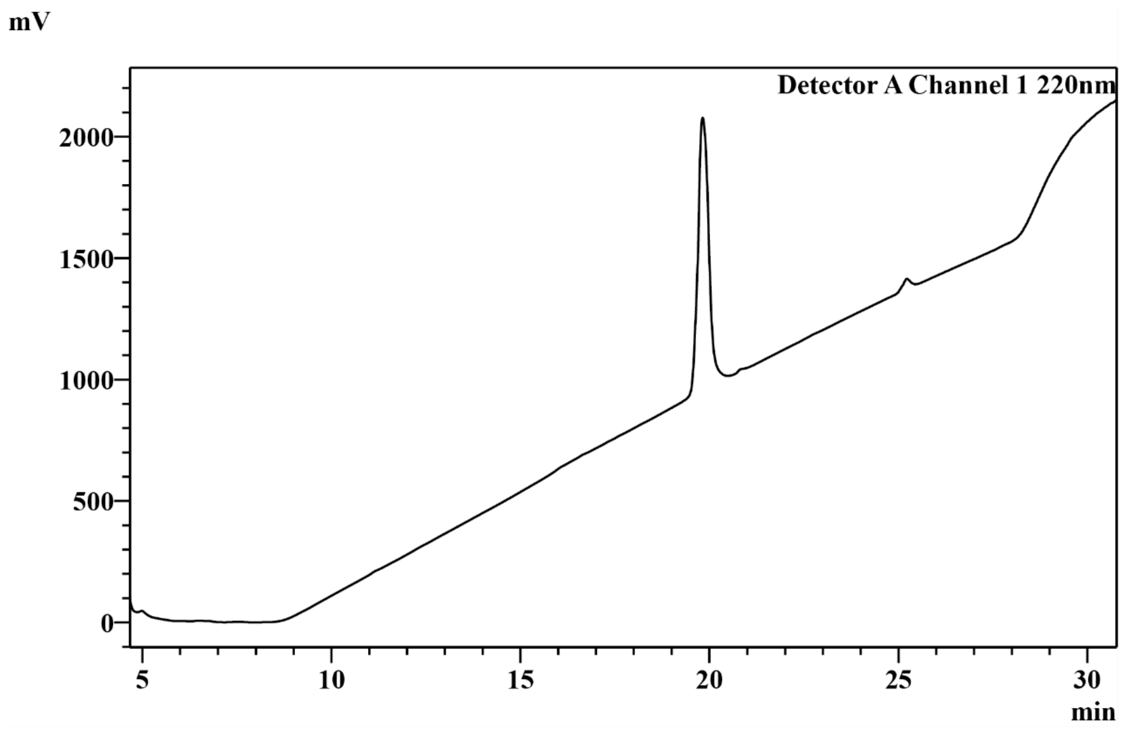

Analytical HPLC chromatogram of purified protein **GB1.**

B.

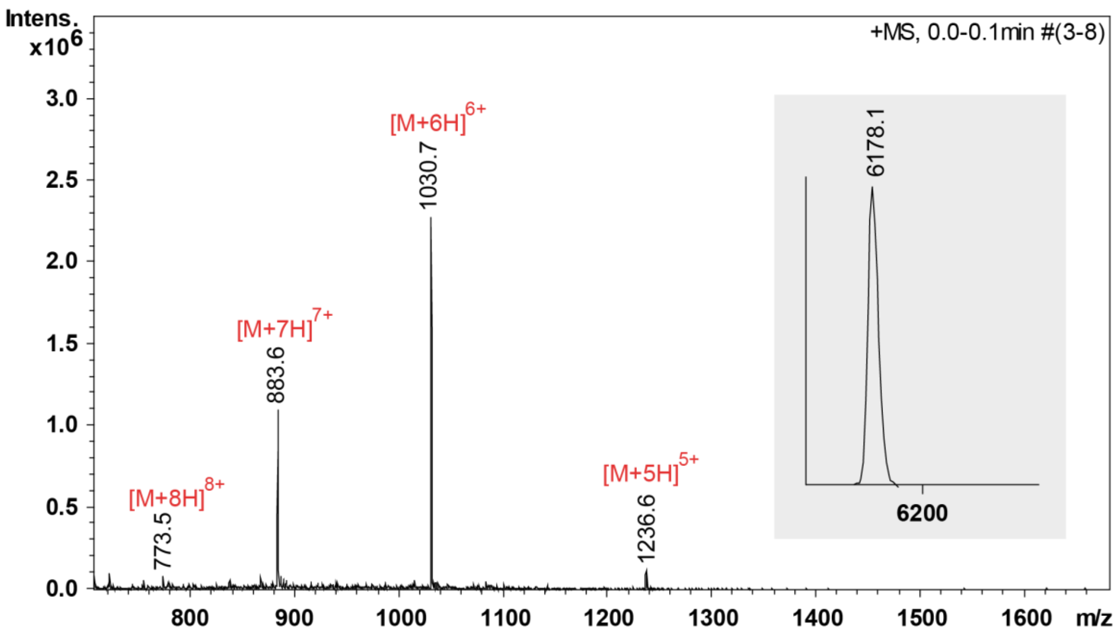

ESI MS analysis of purified protein **GB1** with the deconvoluted mass spectrum.

Figure S2.

A.

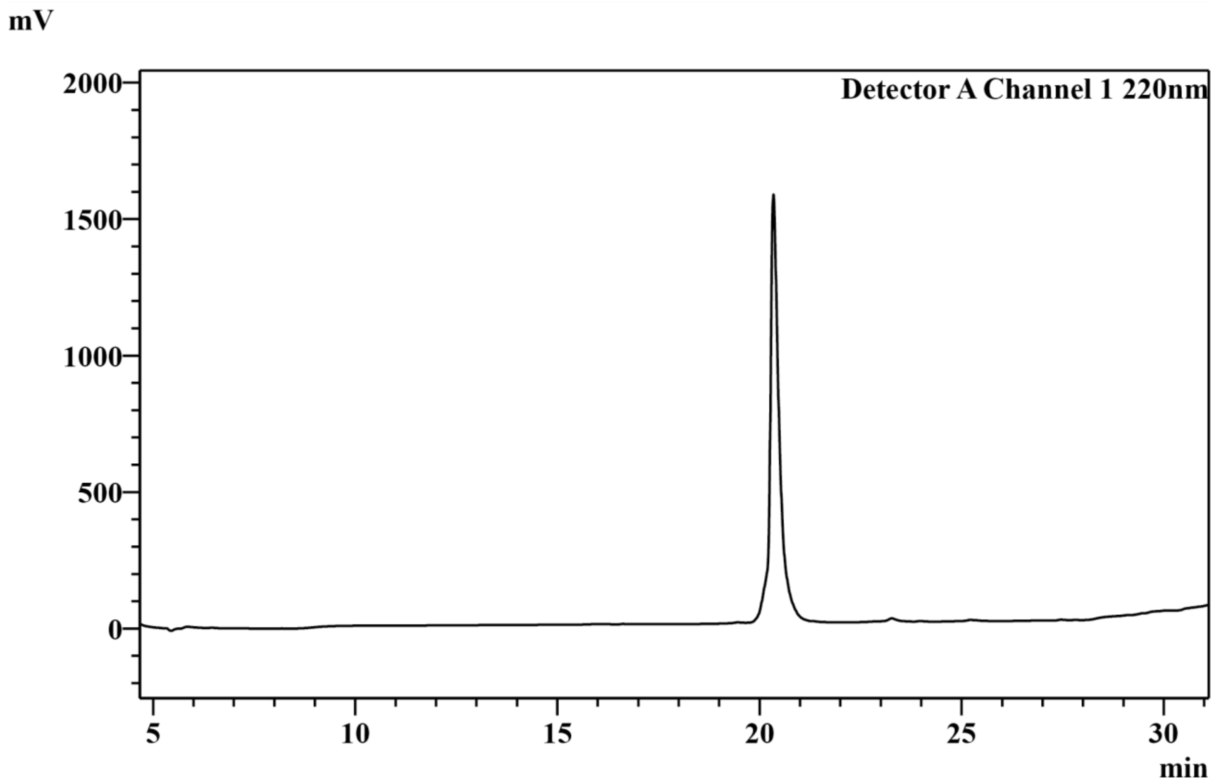

Analytical HPLC chromatogram of purified protein **1.**

B.

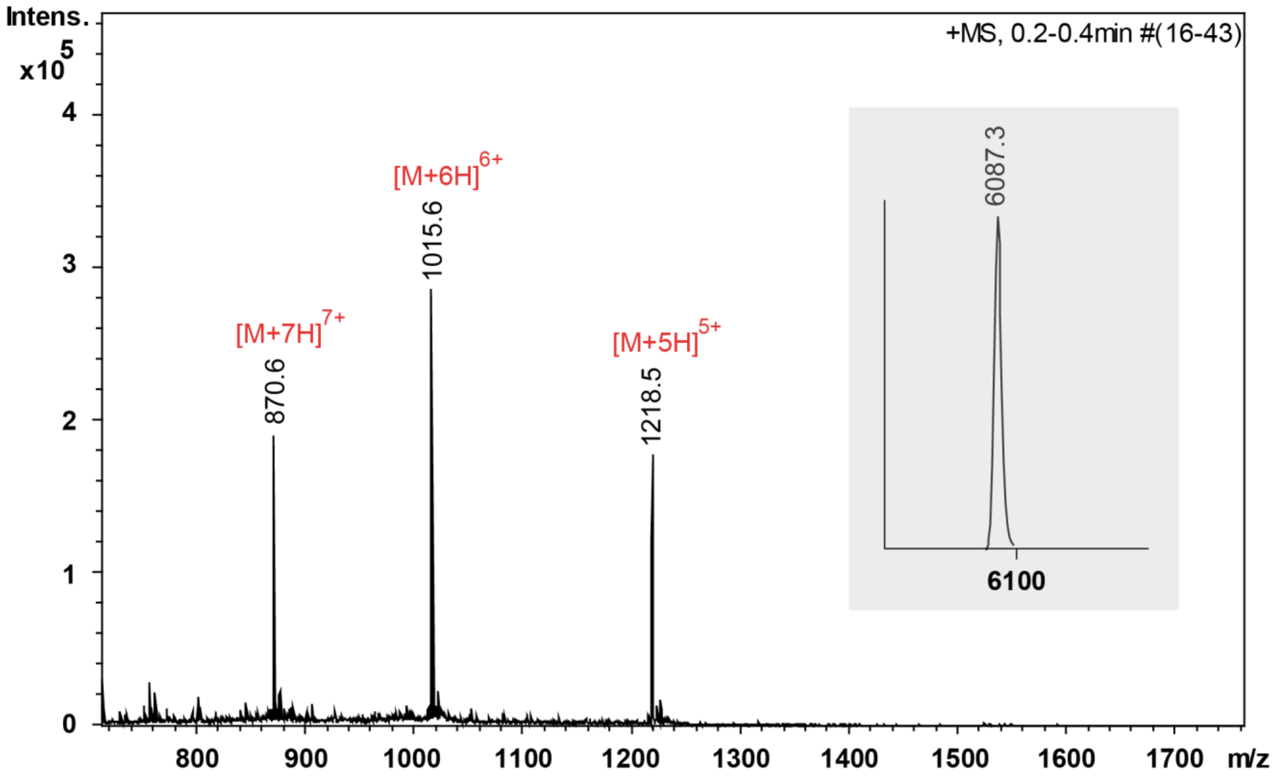

ESI MS analysis of purified protein **1** with the deconvoluted mass spectrum.

Figure S3.

A.

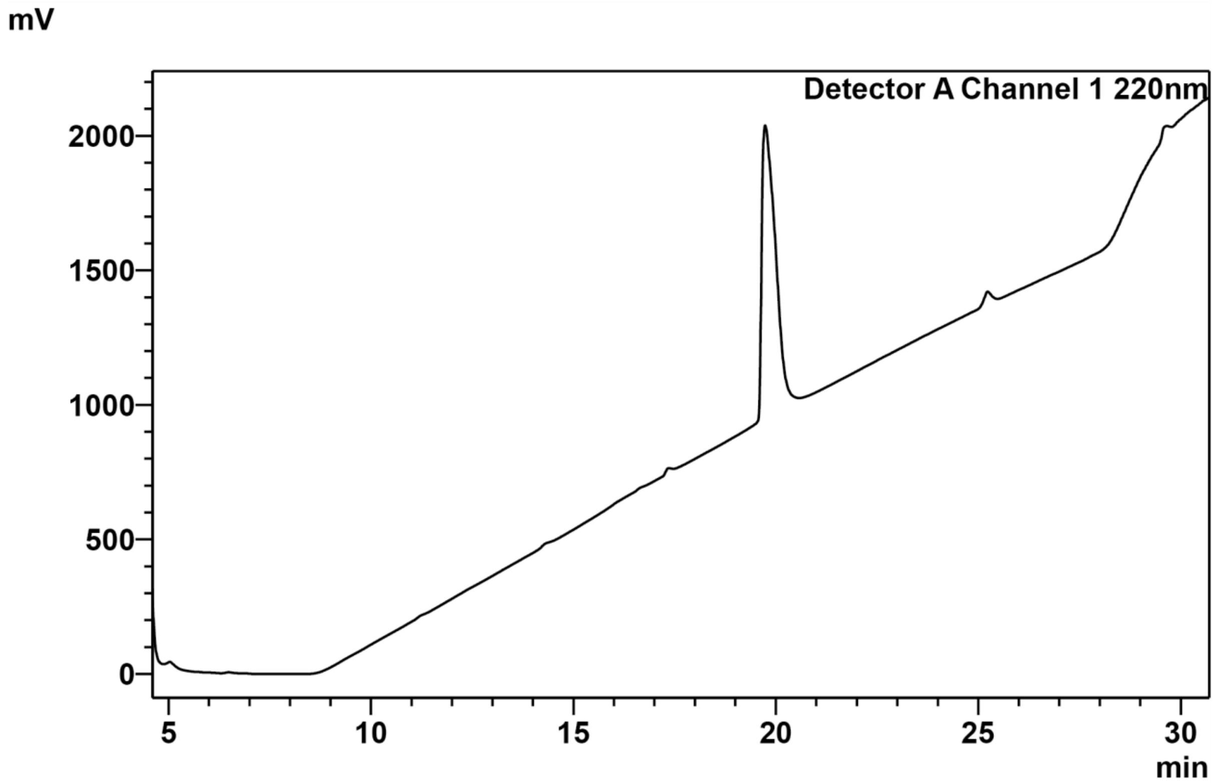

Analytical HPLC chromatogram of purified protein **2.**

B.

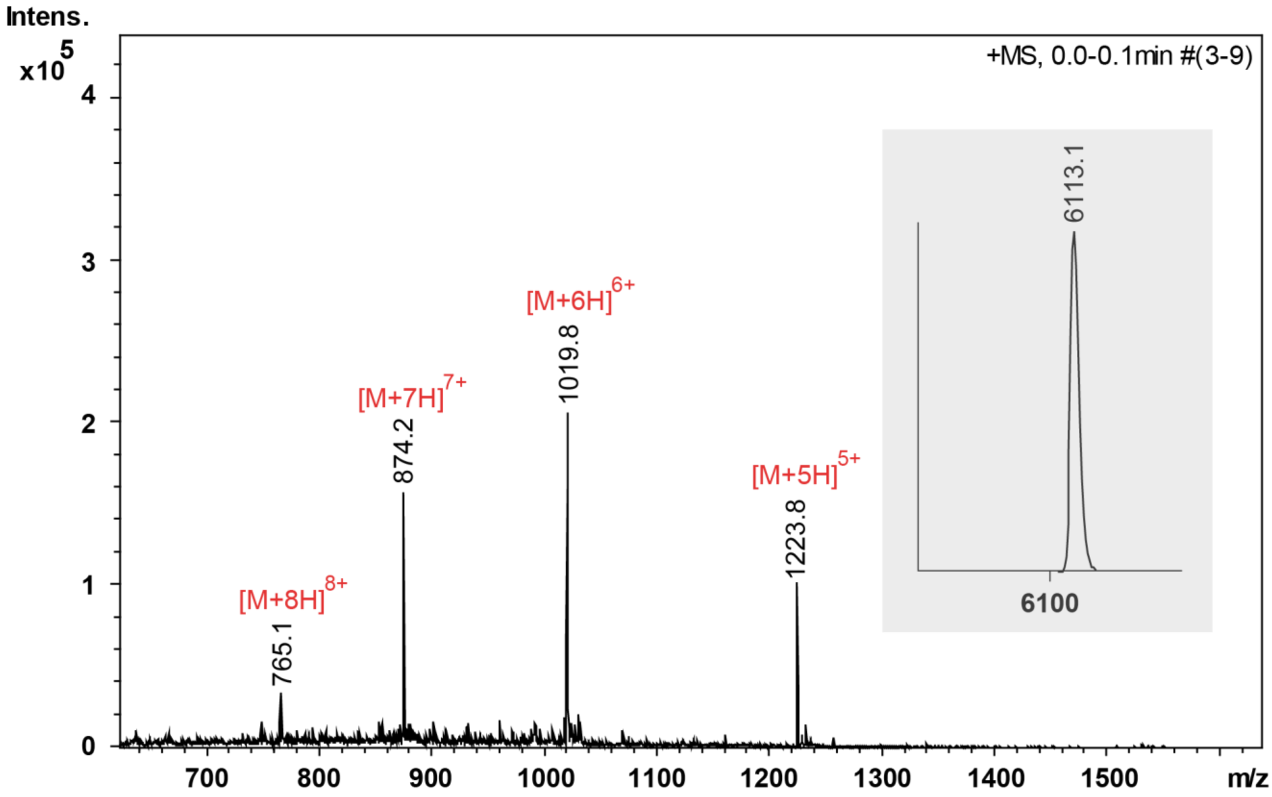

ESI MS analysis of purified protein **2** with the deconvoluted mass spectrum.

Figure S4.

A.

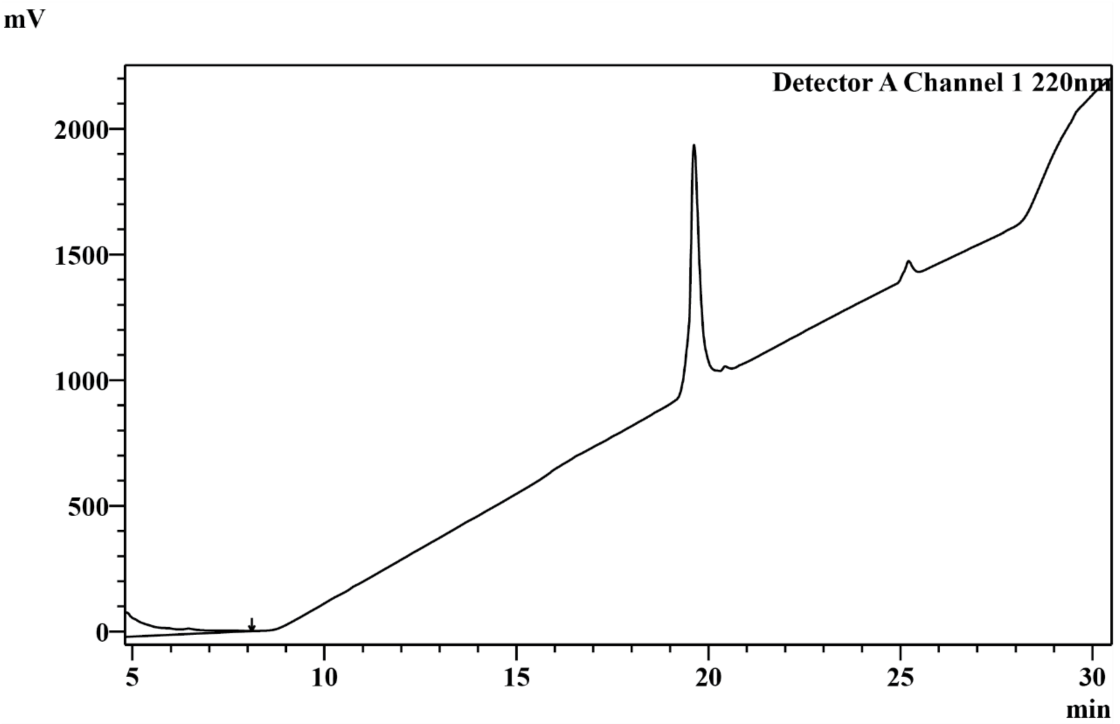

Analytical HPLC chromatogram of purified protein **3.**

B.

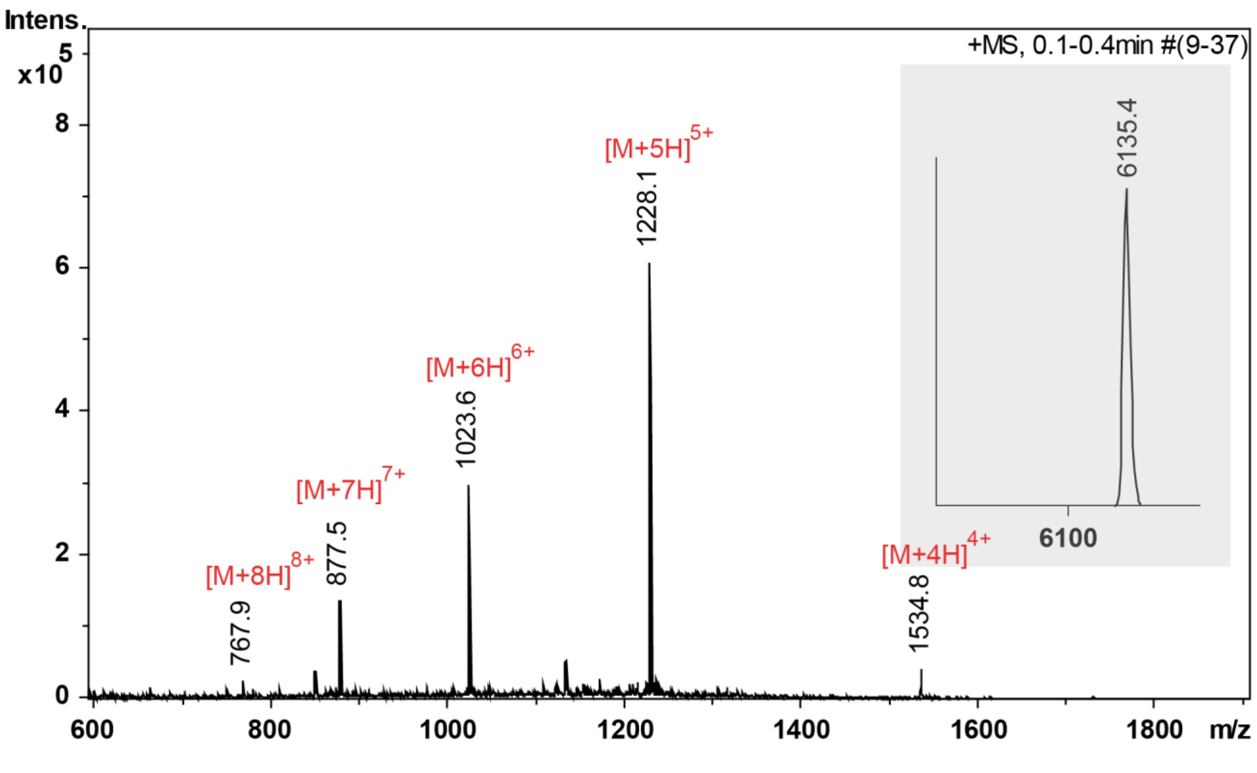

ESI MS analysis of purified protein **3** with the deconvoluted mass spectrum.

Figure S5.

A.

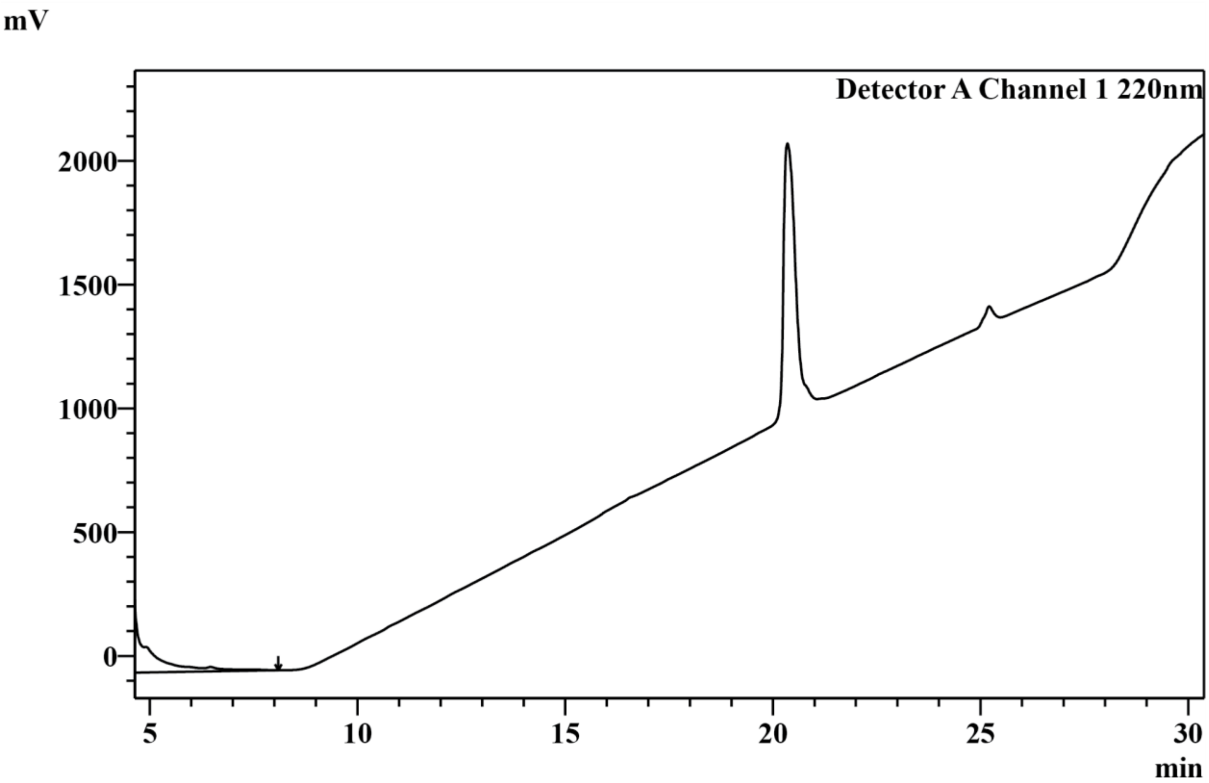

Analytical HPLC chromatogram of purified protein **4.**

B.

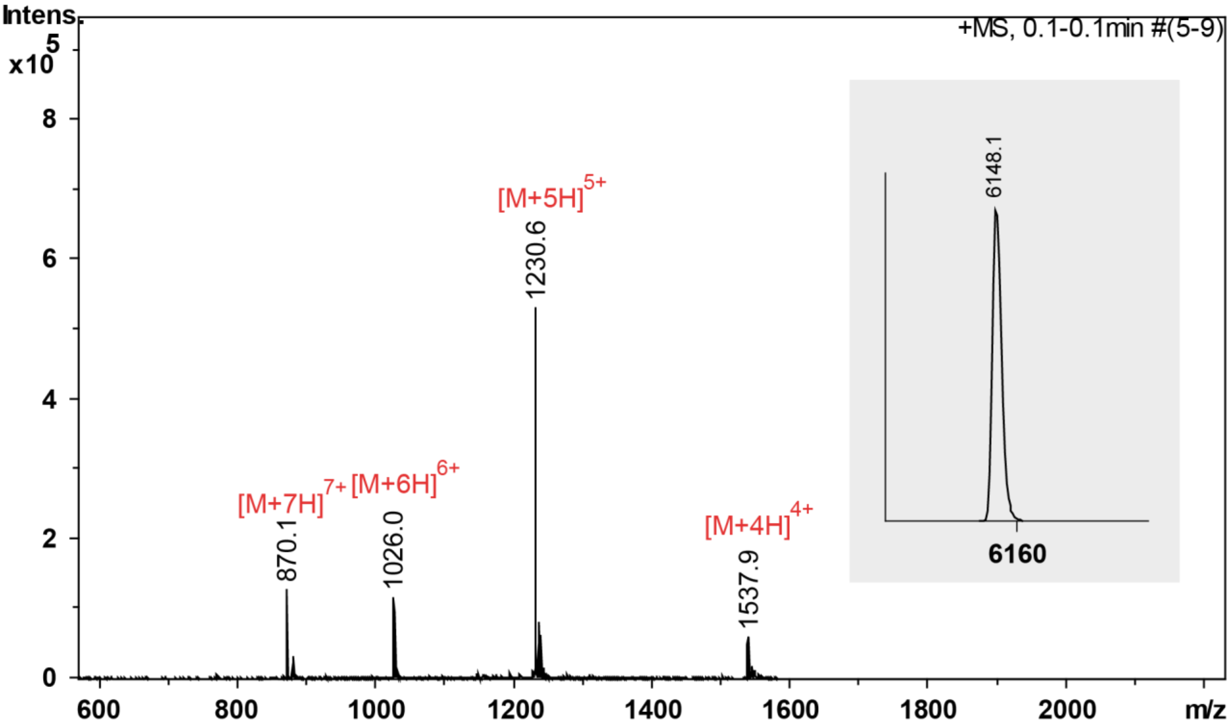

ESI MS analysis of purified protein **4** with the deconvoluted mass spectrum.

Figure S6.

A.

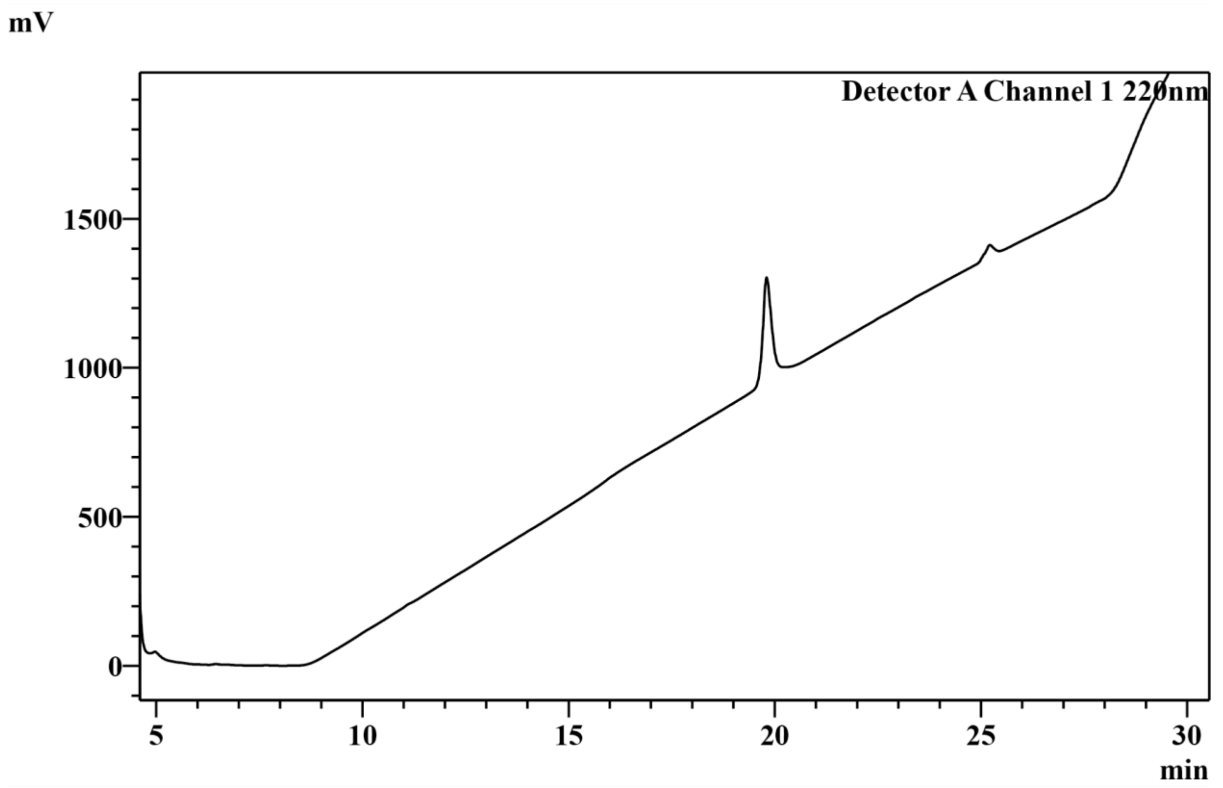

Analytical HPLC chromatogram of purified protein **5.**

B.

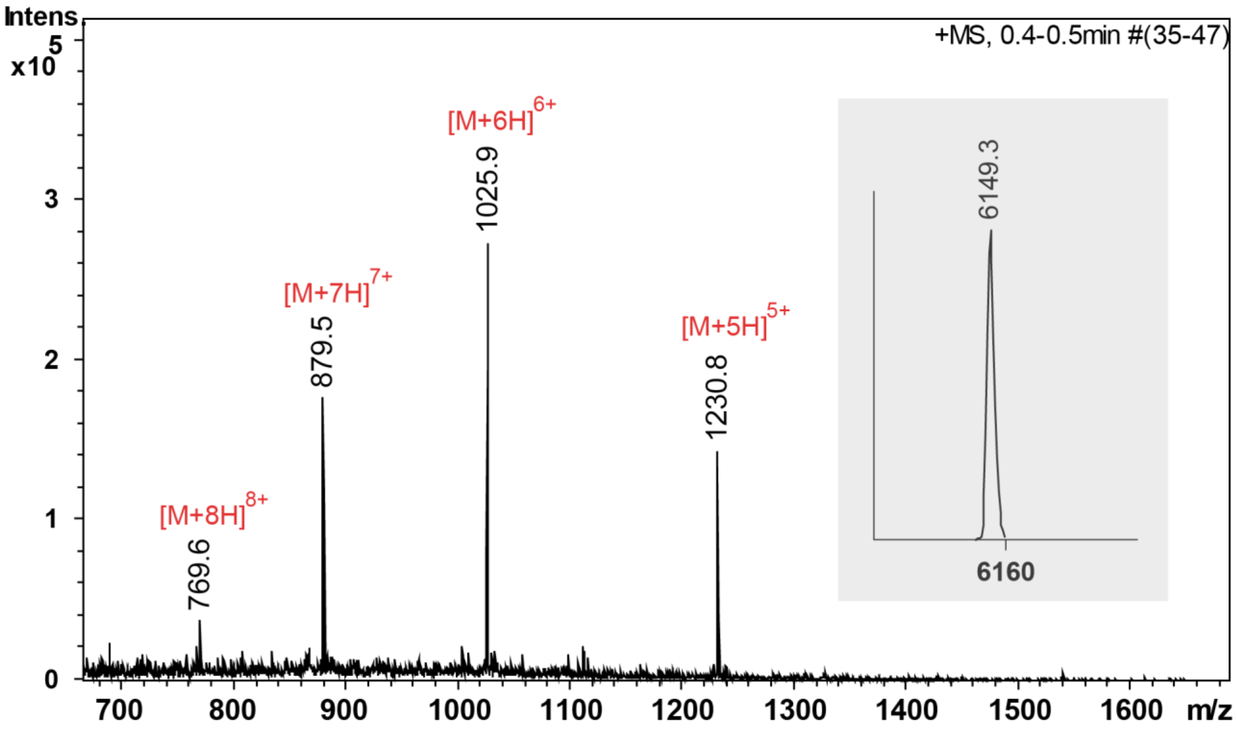

ESI MS analysis of purified protein **5** with the deconvoluted mass spectrum.

Figure S7.

A

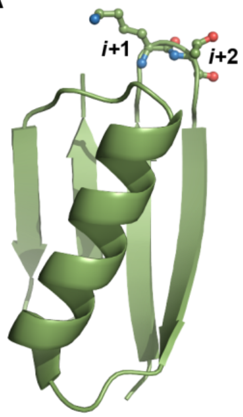

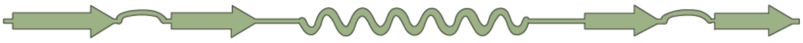

**GB1**	H_2N-DTYKLILNG-KT-LKGETTTEAVDAATAEKVFKQYANDNGVDGEWTYDDATKTFTVTE-$CONH_2$
**1**	H_2N-DTYKLILNG-aA-LKGETTTEAVDAATAEKVFKQYANDNGVDGEWTYDDATKTFTVTE-$CONH_2$
**2**	H_2N-DTYKLILNG-vA-LKGETTTEAVDAATAEKVFKQYANDNGVDGEWTYDDATKTFTVTE-$CONH_2$
**3**	H_2N-DTYKLILNG-vS-LKGETTTEAVDAATAEKVFKQYANDNGVDGEWTYDDATKTFTVTE-$CONH_2$
**4**	H_2N-DTYKLILNG-vV-LKGETTTEAVDAATAEKVFKQYANDNGVDGEWTYDDATKTFTVTE-$CONH_2$
**5**	H_2N-DTYKLILNG-vT-LKGETTTEAVDAATAEKVFKQYANDNGVDGEWTYDDATKTFTVTE-$CONH_2$

**Protein**	***i*+1**	***i*+2**	**T_M (°C)**
**GB1**	L-Lys	L-Thr	77.5 ± 0.05
**1**	D-Ala	L-Ala	71.2 ± 0.05
**2**	D-Val	L-Ala	72.2 ± 0.15
**3**	D-Val	L-Ser	70.5 ± 0.05
**4**	D-Val	L-Val	68.9 ± 0.01
**5**	D-Val	L-Thr	73.4 ± 0.05

B

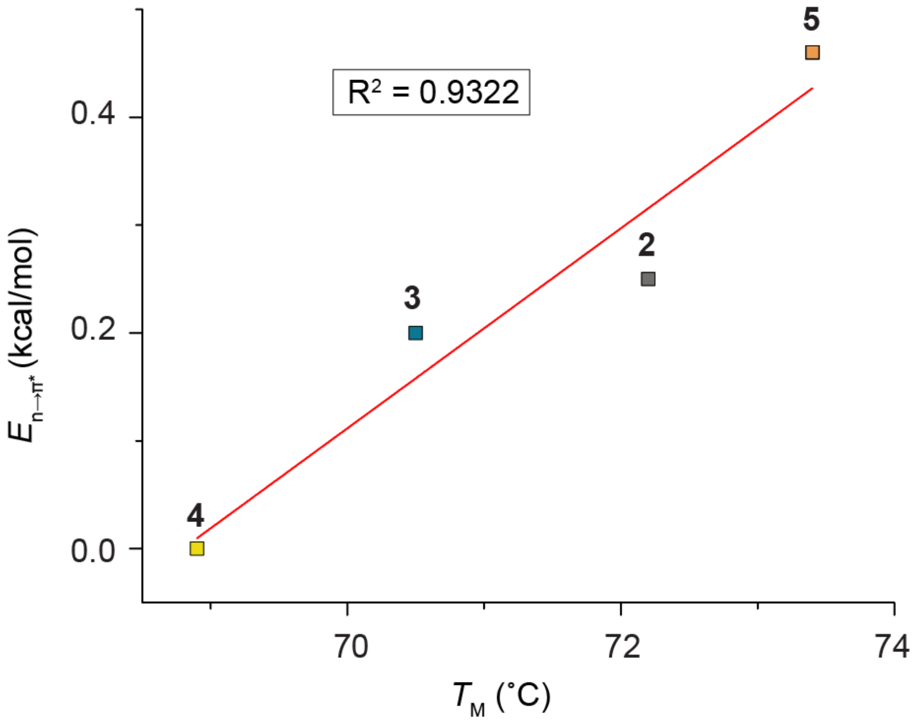

(A) Thermal stability of GB1 variants, and (B) correlation of T_M with $E_{n\rightarrow\pi^*}$ in **2-5.**

Figure S8.

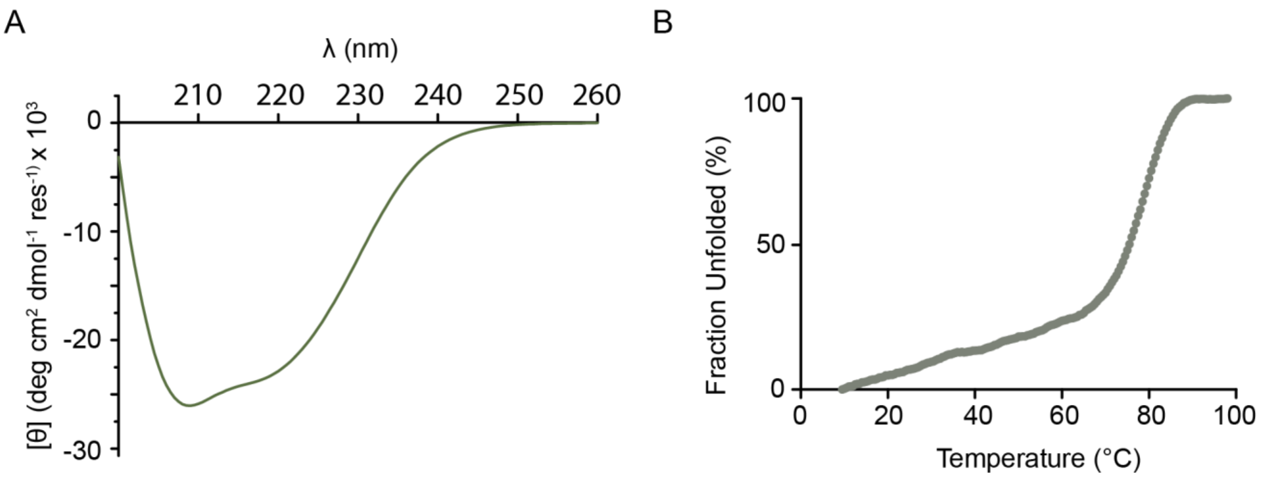

(A) CD scan, and (B) temperature-dependent denaturation of **GB1.**

Figure S9.

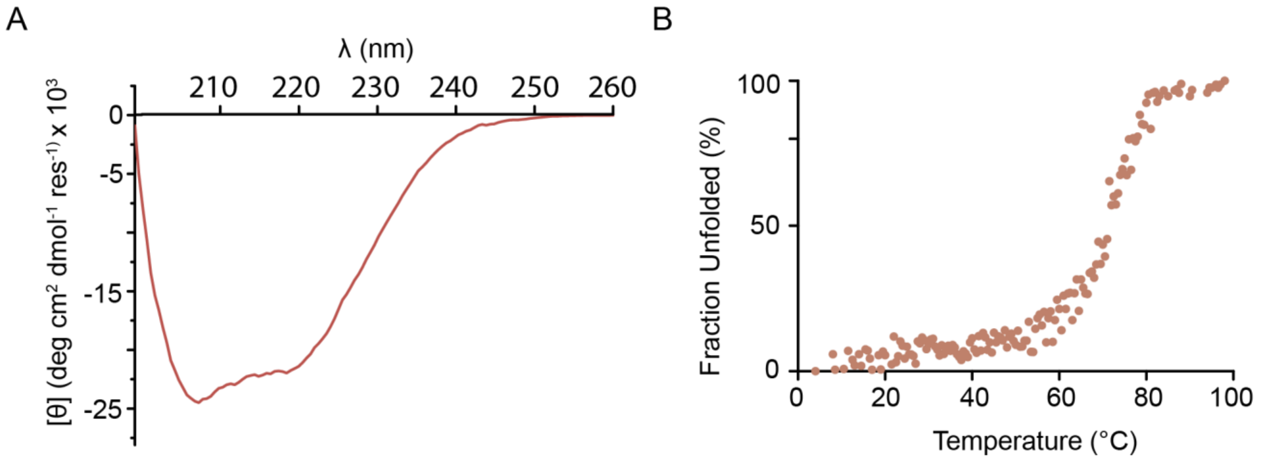

(A) CD scan, and (B) temperature-dependent denaturation of **1.**

Figure S10.

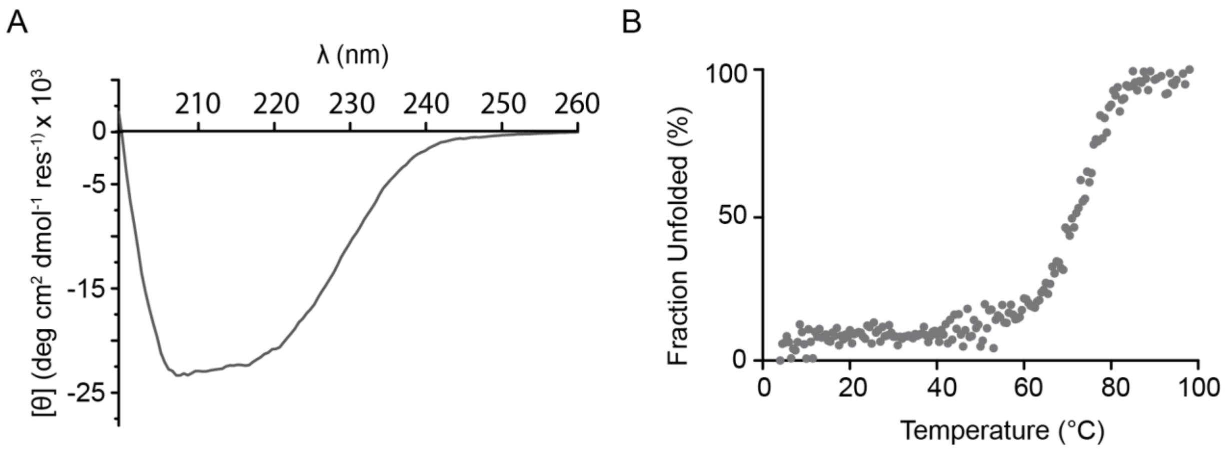

(A) CD scan, and (B) temperature-dependent denaturation of **2.**

Figure S11.

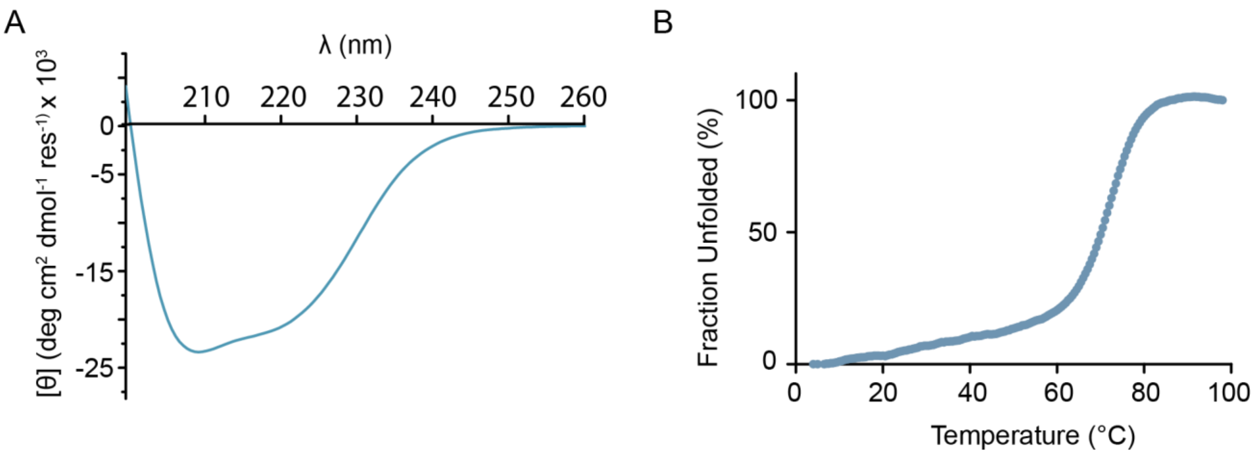

(B) CD scan, and (B) temperature-dependent denaturation of **3.**

Figure S12.

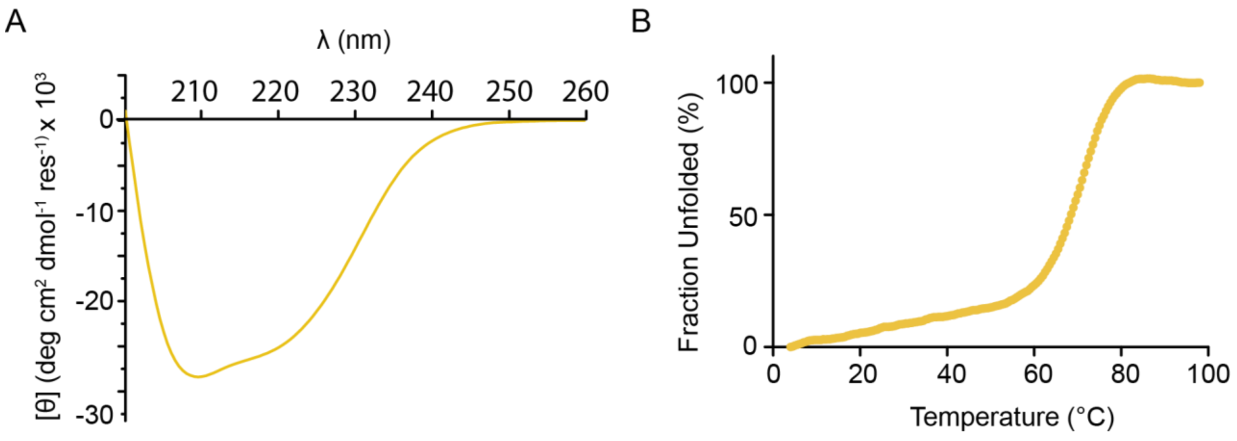

(A) CD scan, and (B) temperature-dependent denaturation of **4.**

Figure S13.

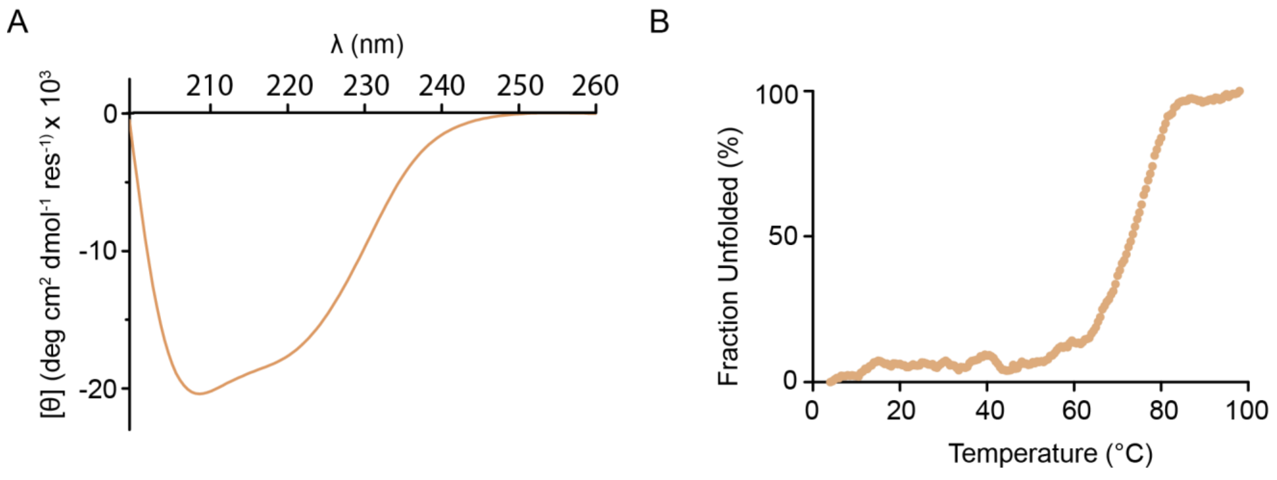

(A) CD scan, and (B) temperature-dependent denaturation of **5.**

Table S2. X-ray diffraction data collection and refinement statistics for proteins 2-5.

	Protein **2**	Protein **3**	Protein **4***	Protein **5**
**PDB ID**	6L9B	6L9D	6LJI	6L91
**Data Collection**				
Unit cell dimentions (Å, °)	a = 43.8, b = 43.8, c = 48.1	a = 43.6, b = 43.6, c = 48.2	a = 90.7, b = 23.1, c = 48.4	a = 45.7, b = 45.7, c = 47.7
	α = 90, β = 90, γ = 120	α = 90, β = 90, γ = 120	α = 90, β = 109, γ = 90	α = 90, β = 90, γ = 120
Space group	$P3_2$ 2 1	$P3_2$ 2 1	C2	$P3_2$ 2 1
Wavelength (Å)	1.54	1.54	1.54	1.54
Resolution (Å)	37.91-1.9 (2.02-1.95)	37.73-1.73 (1.79-1.73)	38.09-1.84 (1.88-1.84)	47.61-1.84 (1.90-1.84)
Rsym	0.09 (0.361)	0.04 (0.333)	0.02 (0.057)	0.02 (0.04)
Mean I/σI	14.6 (3.8)	39.5 (5.5)	36.3 (13.8)	58.3 (25.9)
Completeness (%)	94.9 (65.4)	99.7 (95.1)	96.0 (78.9)	99.7 (95.8)
Redundancy	9.9 (7.1)	16.1 (10.9)	4 (3.2)	6.9 (4.8)
Mosaicity (°)	0.47	0.23	0.24	0.24
CC 1/2	0.99 (0.96)	1 (0.97)	1 (0.99)	1 (0.99)
**Refinement statistics**				
Resolution (Å)	1.9	1.73	1.84	1.84
No. reflection (total/unique)	38866 (1480/ 3909 (208)	93326 (3221)/ 5814 (296)	32240 (1302)/ 8079 (402)	36249 (1496)/ 5245 (313)
R_{work} (%)	20.6	21.9	27.8	16.6
R_{free} (%)	24.9	24.2	32.8	18.6
Solvent content (%)	36	28	36	37
Wilson B-factor (Å^2)	26.53	19.23	17.64	10.74
Average B-factor (Å^2)	30.19	22.43	22.55	11.25
RMSD<br>Bond lengths (Å)<br>Bond angles (°)	 0.007<br>0.831	 0.007<br>0.856	 0.006<br>0.754	 0.007<br>0.792
Ramachandran statistics<br>Favoured (%)<br>Allowed (%)<br>Outliers (%)**	 96.3<br>1.85<br>1.85	 98.15<br>0<br>1.85	 97.22<br>0.93<br>1.85	 98.15<br>0<br>1.85

*Protein **4** lattice has translational pseudo symmetry with a peak in the Patterson map at 0, 0, 0.5

Protein **2-5 variants have D-Valine incorporation at 11th position in protein sequence.

Figure S14.

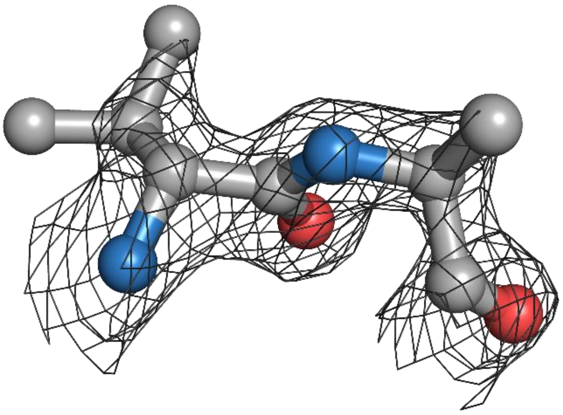

2

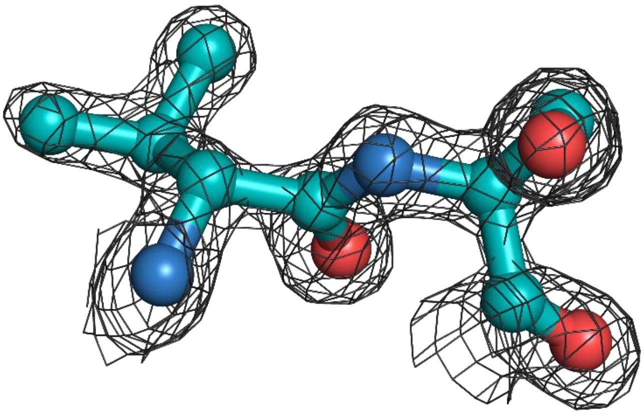

3

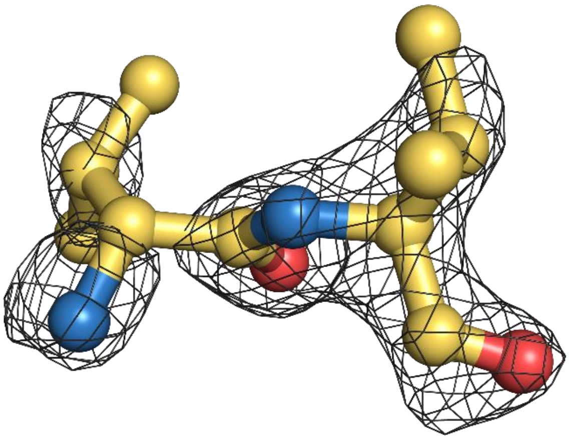

4

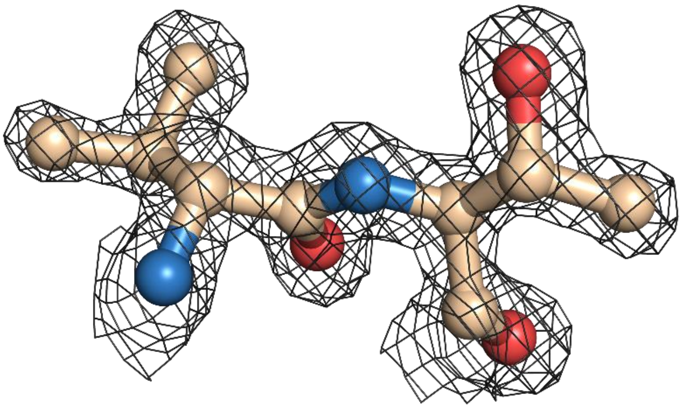

5

The mF_{O}-DF_{C} omit maps (contoured at 3.0σ) from the refined structures of proteins **2-5.**

Figure S15.

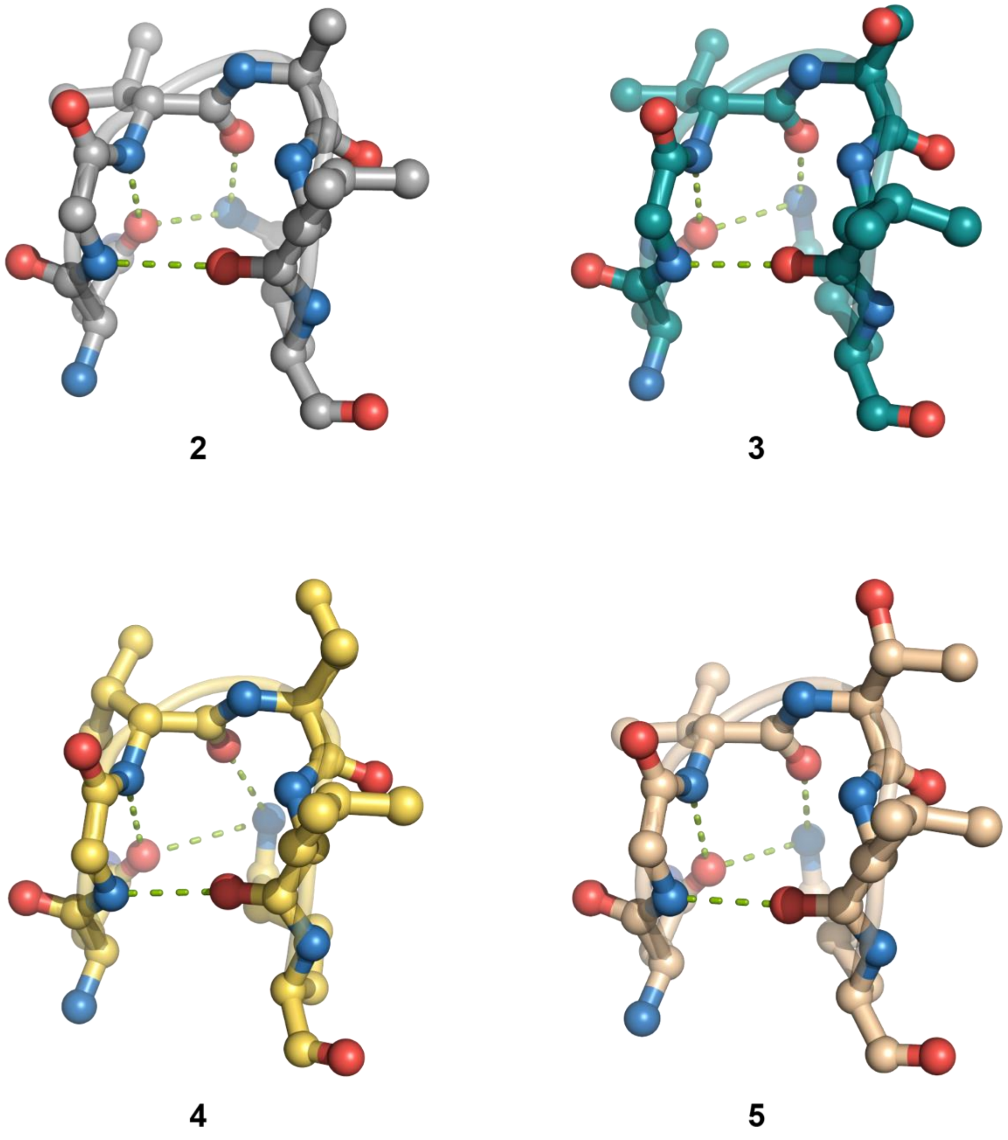

Intramolecular hydrogen bonding at the loopL1 in **2**-**5**. Asn8 to Lys13 are shown.

Table S3. Overlap of n and π^* orbitals in proteins 2-5.

Protein	ChemDraw Presentation	Overlap of n and π^* orbitals $C{=}O_{i+1} \rightarrow C{=}O_{i+2}$	ChemDraw Presentation	Overlap of n and π^* orbitals $C{=}S_{i+1} \rightarrow C{=}O_{i+2}$
**2**	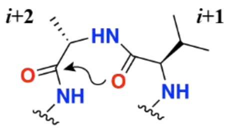	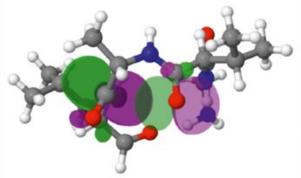 $E_{n\rightarrow\pi*}$ = 0.25 kcal/mol	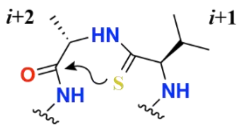	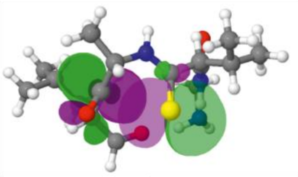 $E_{n\rightarrow\pi*}$ = 0.95 kcal/mol
**3**	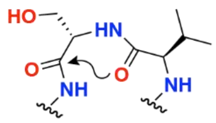	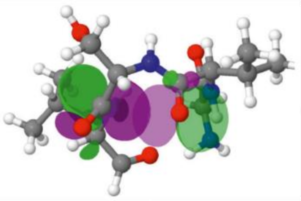 $E_{n\rightarrow\pi*}$ = 0.20 kcal/mol	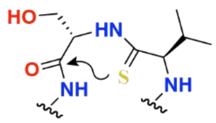	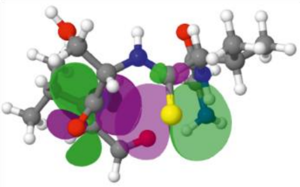 $E_{n\rightarrow\pi*}$ = 1.01 kcal/mol
**4**	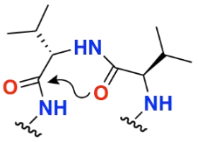	no overlap	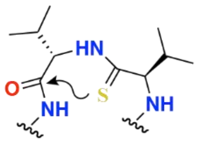	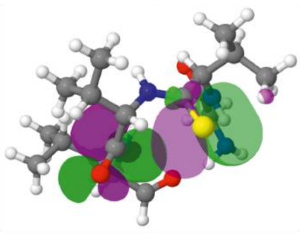 $E_{n\rightarrow\pi*}$ = 0.06 kcal/mol
**5**	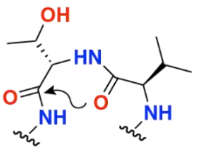	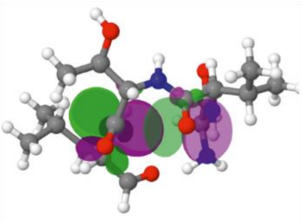 $E_{n\rightarrow\pi*}$ = 0.46 kcal/mol	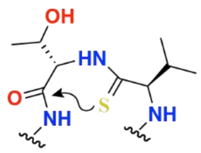	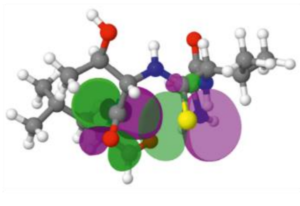 $E_{n\rightarrow\pi*}$ = 1.37 kcal/mol

Table S4. Threonine side-chain rotameric conformation in *β*-turns.

Turn	PDB_ID	Chain	Residue No.	Sequence	*i*+1		*i*+2		*d* (Å)	*θ* (°)	Side-chain rotamer (χ_1, °)
					φ (°)	*ψ* (°)	*φ* (°)	*ψ* (°)			
type I	3B1B	A	135-138	NQTN	-62.7	-15.3	-75.9	-18.9	3.2	120	*g*- (75.2)
	2DC3	A	177-180	NATT	-63.9	-25.3	-83.5	-13.5	3.35	125.3	*g*- (73.5)
	4GF6	A	48-51	CTTG	-84.1	-11.7	-92.7	-3.5	3.53	128.6	*g*- (46.4)
	1LQA	A	20-23	TMTF	-67.2	-11.1	-92.6	-1.1	3.41	128.5	*g*- (62.0)
	1U1D	A	222-225	NRTQ	-67.3	-15.5	-90	1.9	3.49	134.6	*g*- (68.7)
	1PZS	A	60-63	APTG	-49.2	-37.7	-100.7	7.8	3.62	138.1	*g*- (66.5)
	2H26	A	20-23	NSTW	-64	-19.7	-111.5	1.5	3.67	129.3	*g*- (56.7)
	1ET7	A	88-91	PETN	-66.1	-14.4	-86.4	-7.7	3.43	129	*g*- (53.7)
type II	1BS9	A	12-15	ETTA	-58.9	131.8	71.4	-2.9	3.48	146.2	*g*- (73.6)
type II'	1CCW	A	218-221	PLTG	53.8	-120.1	-78.1	-0.7	3.21	134.3	*g*- (51.4)
	1D3Y	A	170-173	LGTG	65.1	-130.6	-66.1	-32.8	3.04	111.1	*g*+ (-59.8)
	1E8C	A	415-418	PRTE	49.8	-127.6	-90.7	3.9	3.45	135.1	*g*- (73.3)
	1FP2	A	197-200	GGTG	64.6	-132.9	-95.8	11	3.46	142.1	*g*- (56.4)
	1G8K	A	557-560	PGTA	42.4	-123.8	-104.2	22.9	3.67	149.9	*g*- (61.4)
		A	707-710	WQTA	48.2	-126.7	-79.3	12.6	3.32	145.3	*g*- (55.6)
	1H0H	A	861-864	WQTG	58.2	-130.3	-69.7	-1.8	3	134.7	*g*- (67.2)
	1H6W	A	379-382	IGTR	53.3	-140	-98.1	24.8	3.56	147.2	*g*- (52.9)
	4ARC	A	330-333	YGTG	51.2	-134.1	-88.4	0.8	3.5	134.4	*g*- (64.0)
	2WAN	A	526-529	EGTA	60.4	-137.2	-101.3	7.4	3.59	138.1	*g*- (61.4)
	2YA0	A	636-639	LGTA	50.7	-147.1	-82	-28.2	3.25	108.8	*g*+ (-95.2)
	1KWG	A	603-606	RGTW	66.7	-134.9	-87.7	4.3	3.38	137.5	*g*- (62.7)

Figure S16.

A.

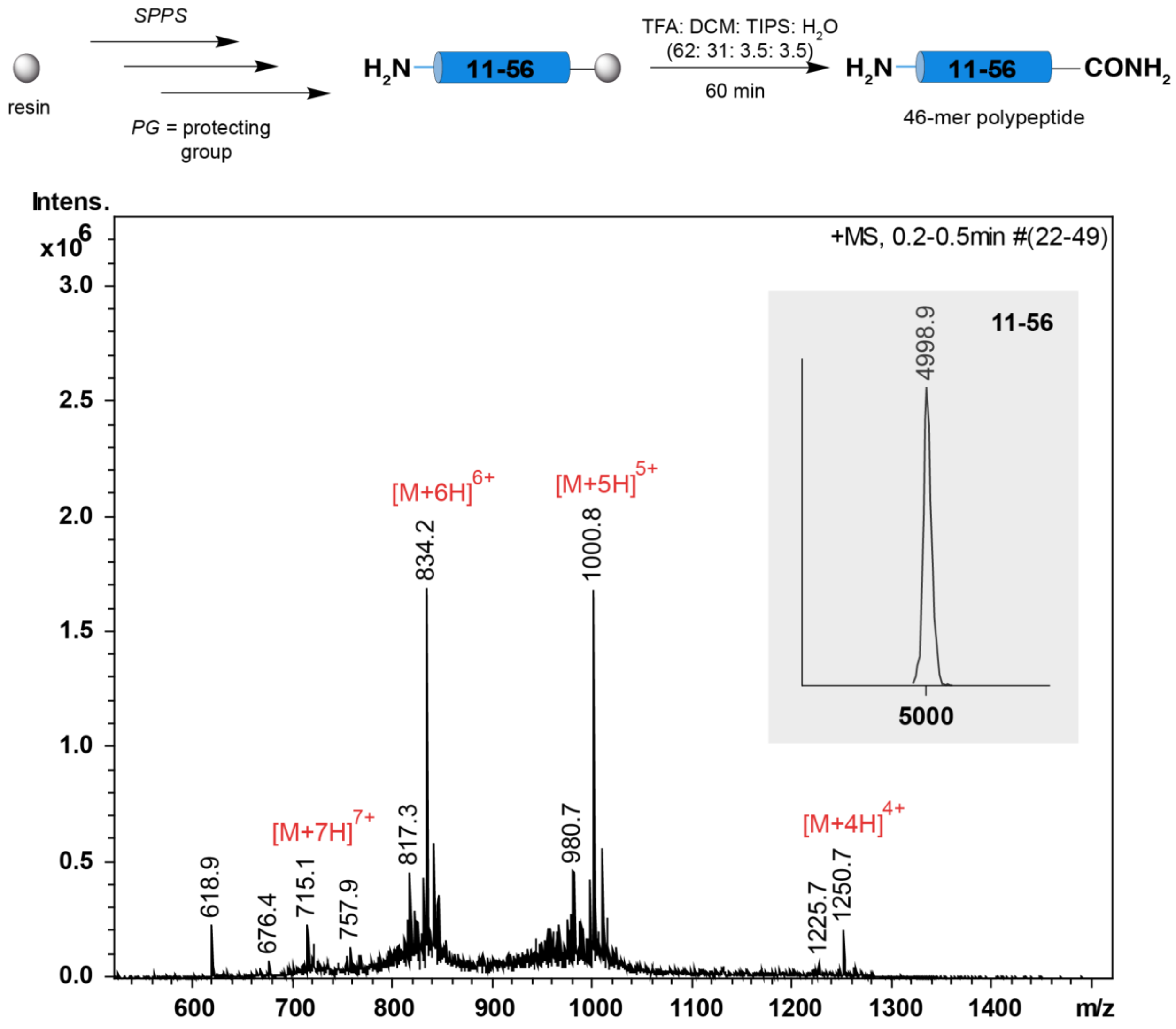

ESI MS analysis of crude 46-mer oxo-polypeptide with the deconvoluted mass spectrum.

B.

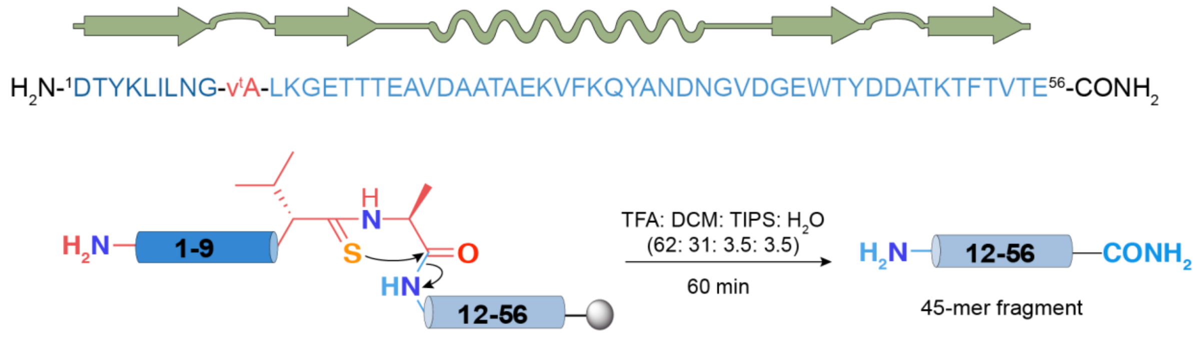

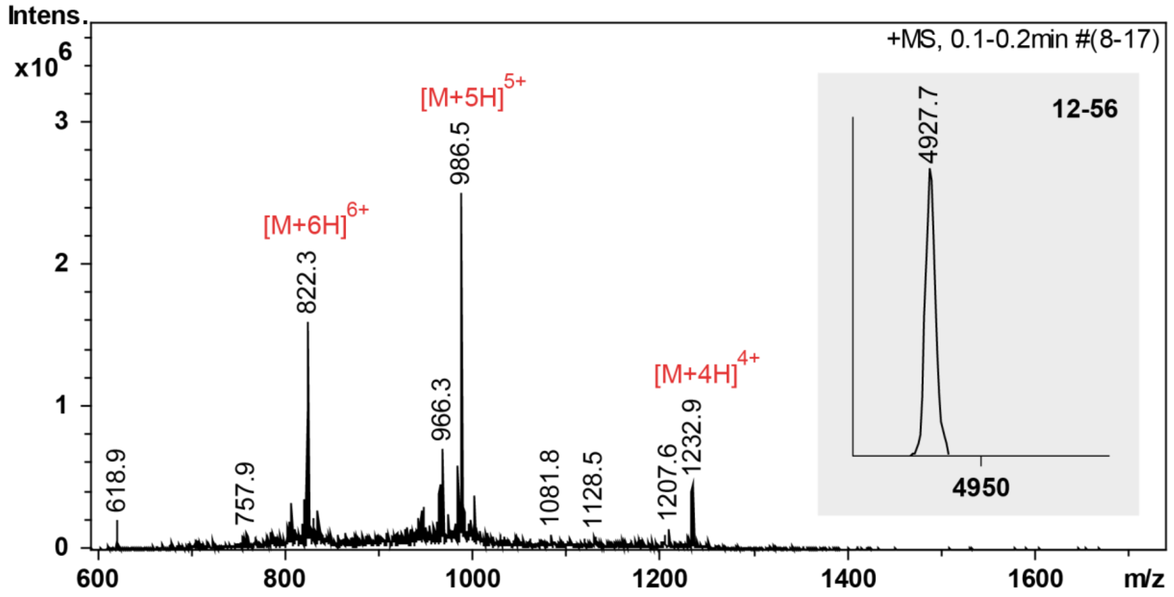

The acid-catalyzed cleavage of **2a** yielding C-terminal fragment (12-56).

C.

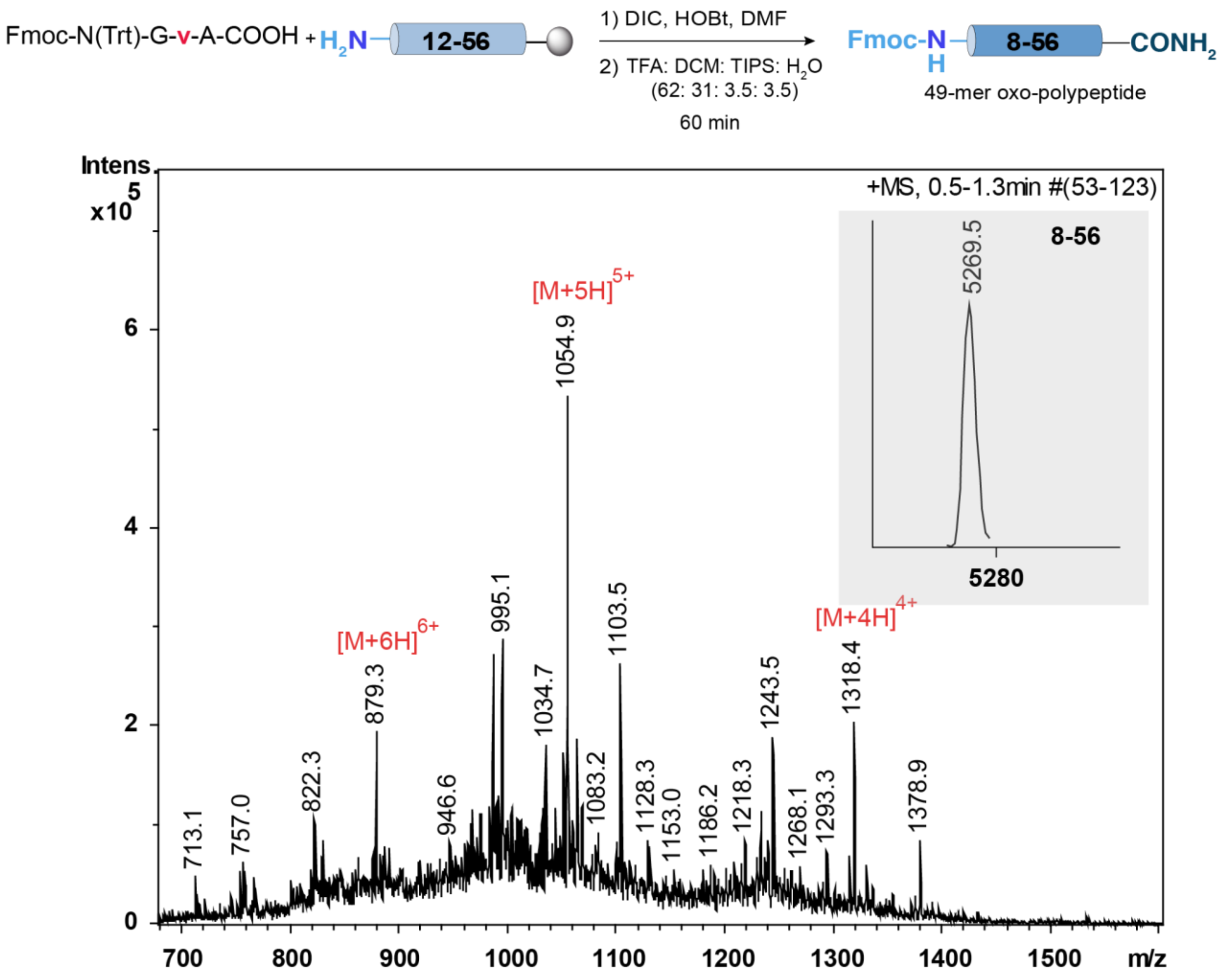

ESI MS analysis of crude 49-mer oxo-polypeptide with the deconvoluted mass spectrum.

D.

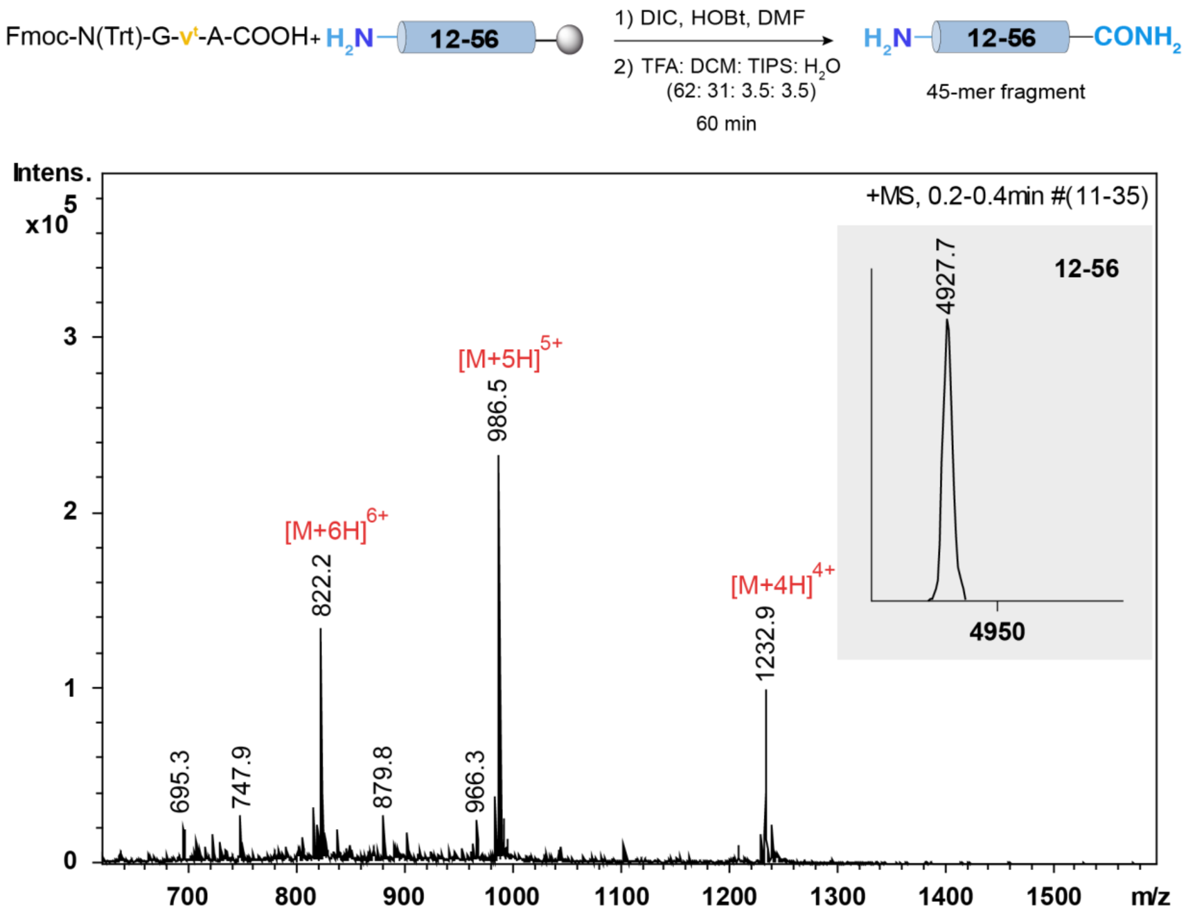

The acid-catalyzed cleavage of 49-mer thio-polypeptide yielding C-terminal fragment (12-56).

E.

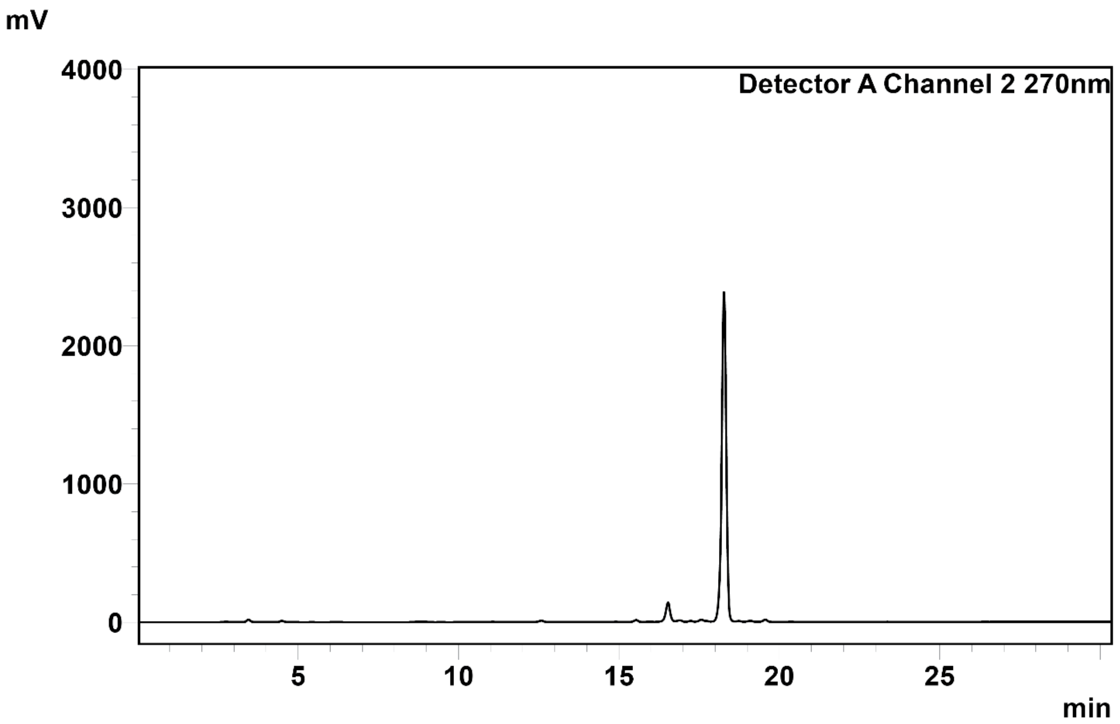

Analytical HPLC chromatogram of crude peptide Fmoc-Asn(Trt)-Gly-D-Val[t]-**L-Ala**-COOH.

F.

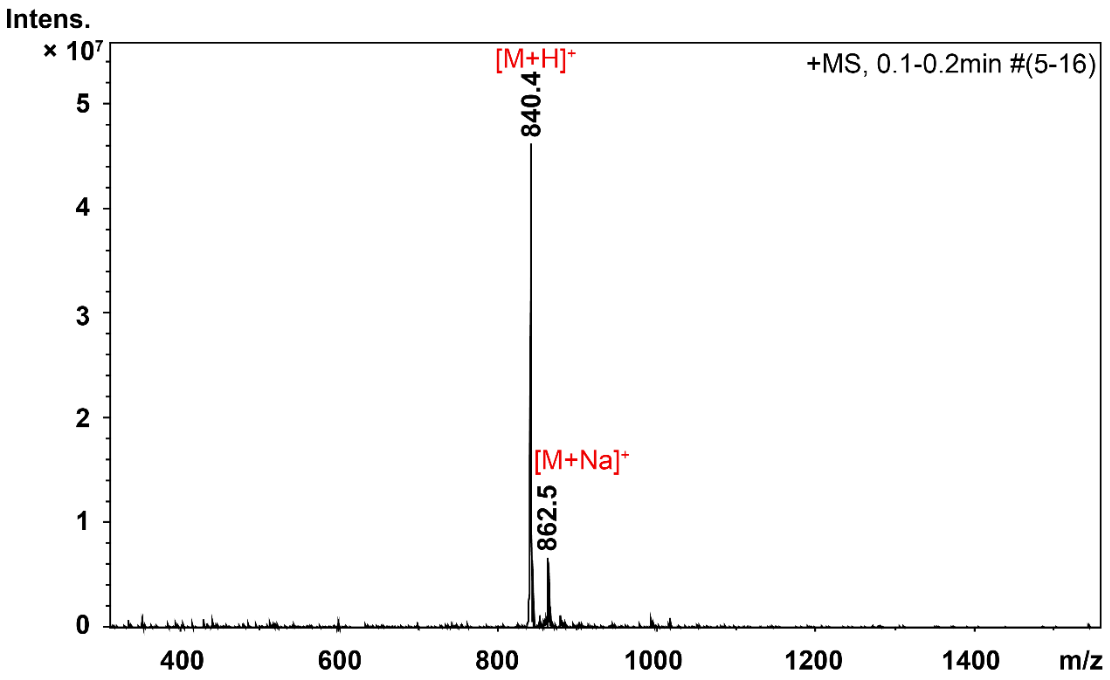

ESI MS analysis of peptide Fmoc-Asn(Trt)-Gly-D-Val[t]-**L-Ala**-COOH.

G.

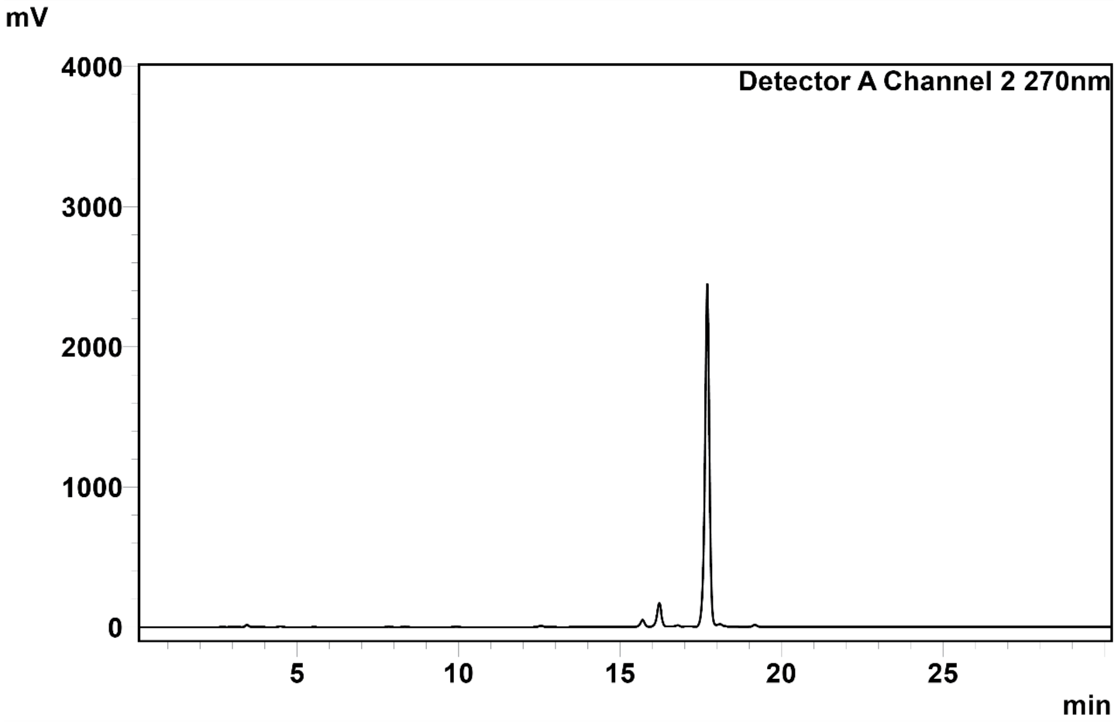

Analytical HPLC chromatogram of crude peptide Fmoc-Asn(Trt)-Gly-D-Val^{t}-**D-Ala**-COOH.

H.

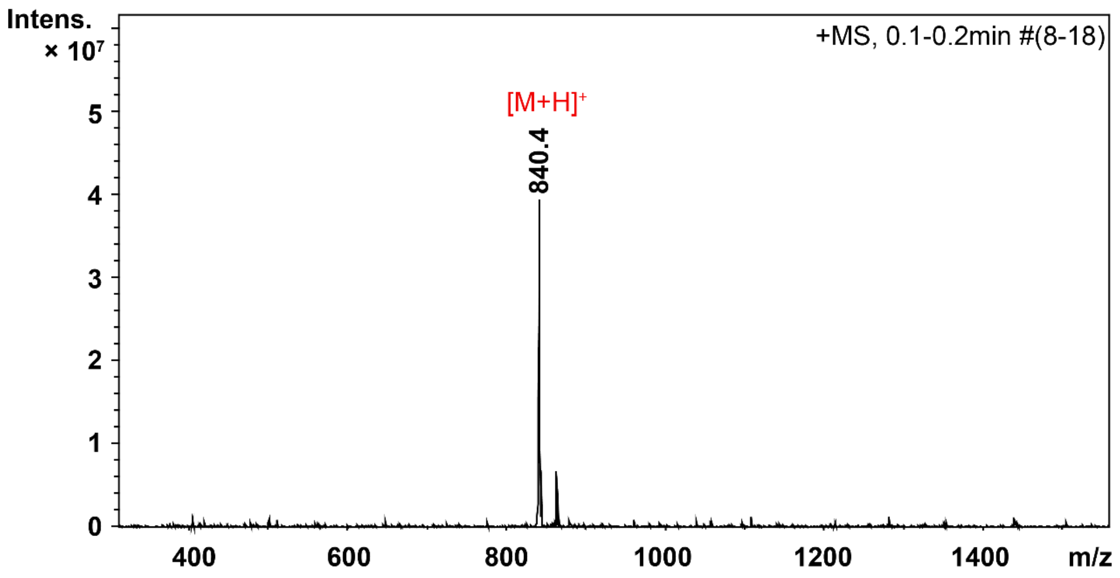

ESI MS analysis of peptide Fmoc-Asn(Trt)-Gly-D-Val^{t}-**D-Ala**-COOH.

I.

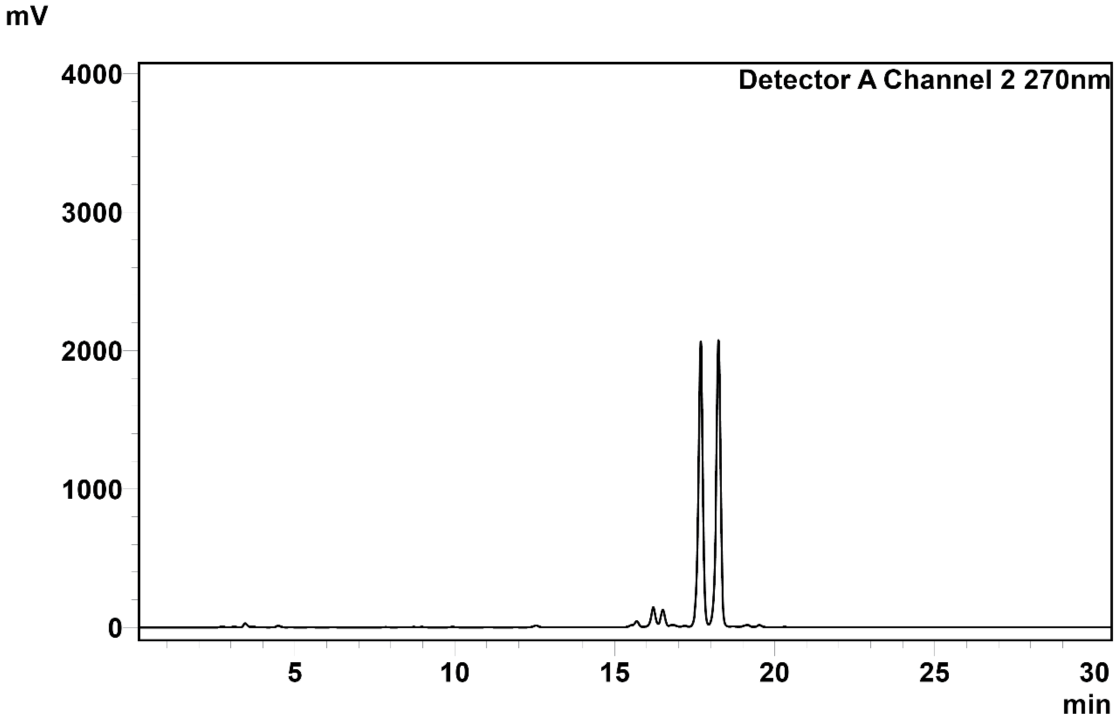

Analytical HPLC chromatogram of the co-injection of crude peptides Fmoc-Asn(Trt)-Gly-D-Val^{t}**-L-Ala-**COOH and Fmoc-Asn(Trt)-Gly-D-Val^{t}**-D-Ala-**COOH.

Figure S17.

A.

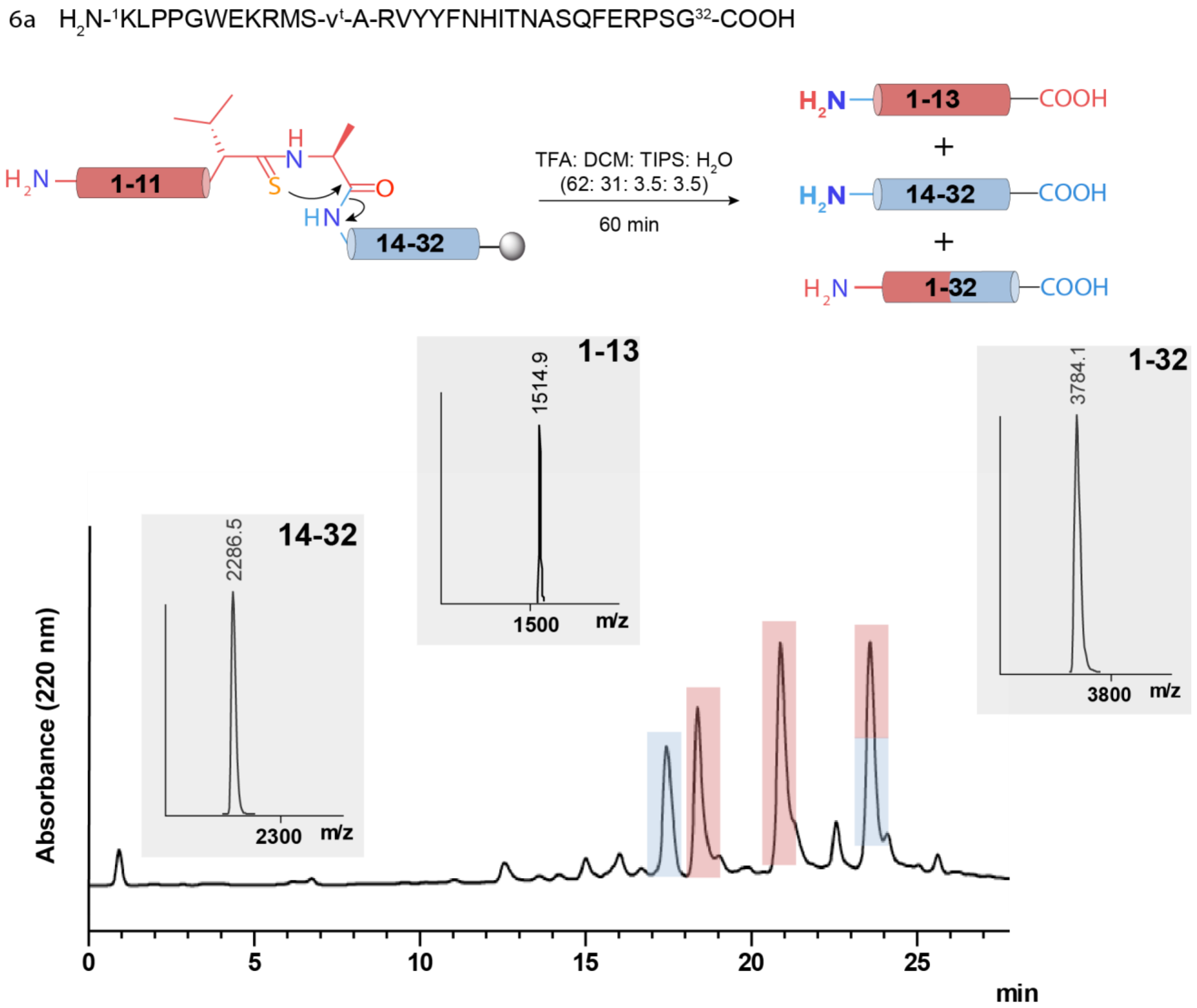

The acid-catalysed cleavage of **6a** (The polypeptides are color coded and the deconvoluted mass spectrum is shown inset).

.

B.

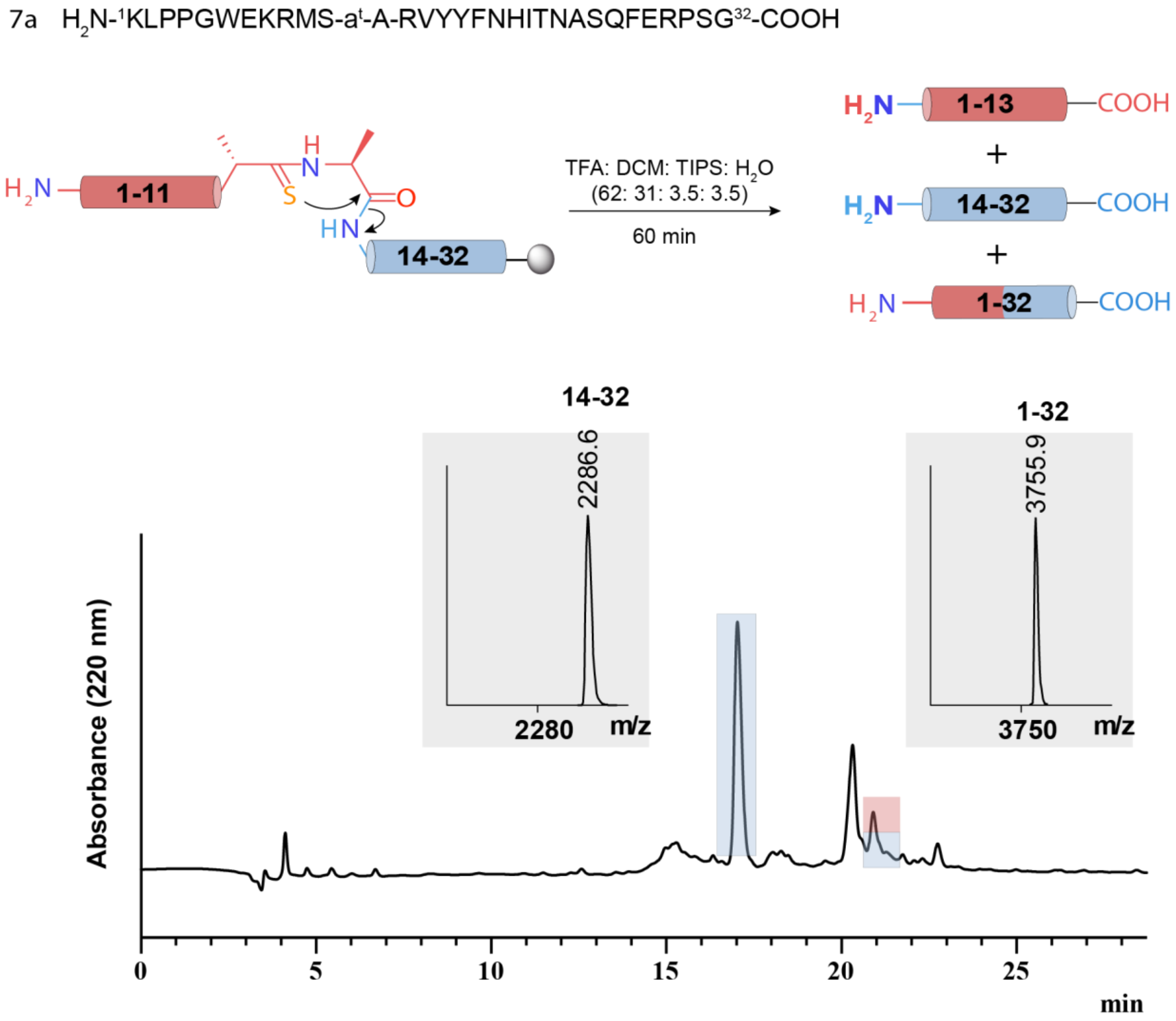

The acid-catalysed cleavage of **7a** (The polypeptides are color coded and the deconvoluted mass spectrum is shown inset).

Figure S18.

A.

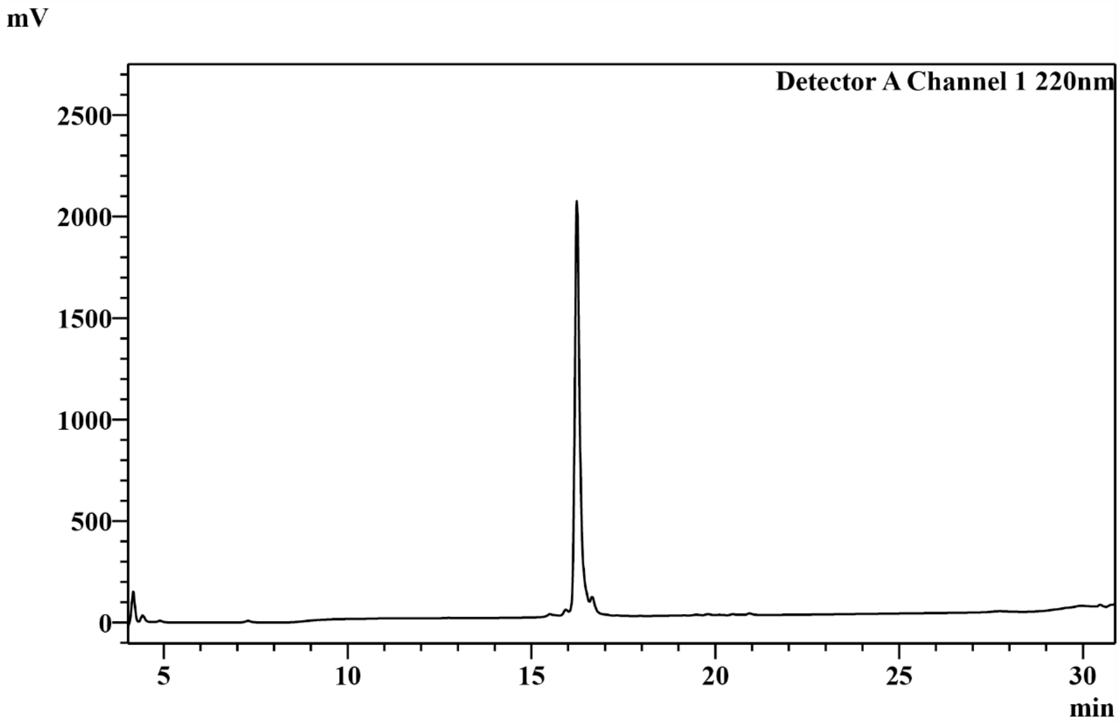

Analytical HPLC chromatogram of purified peptide **6.**

B.

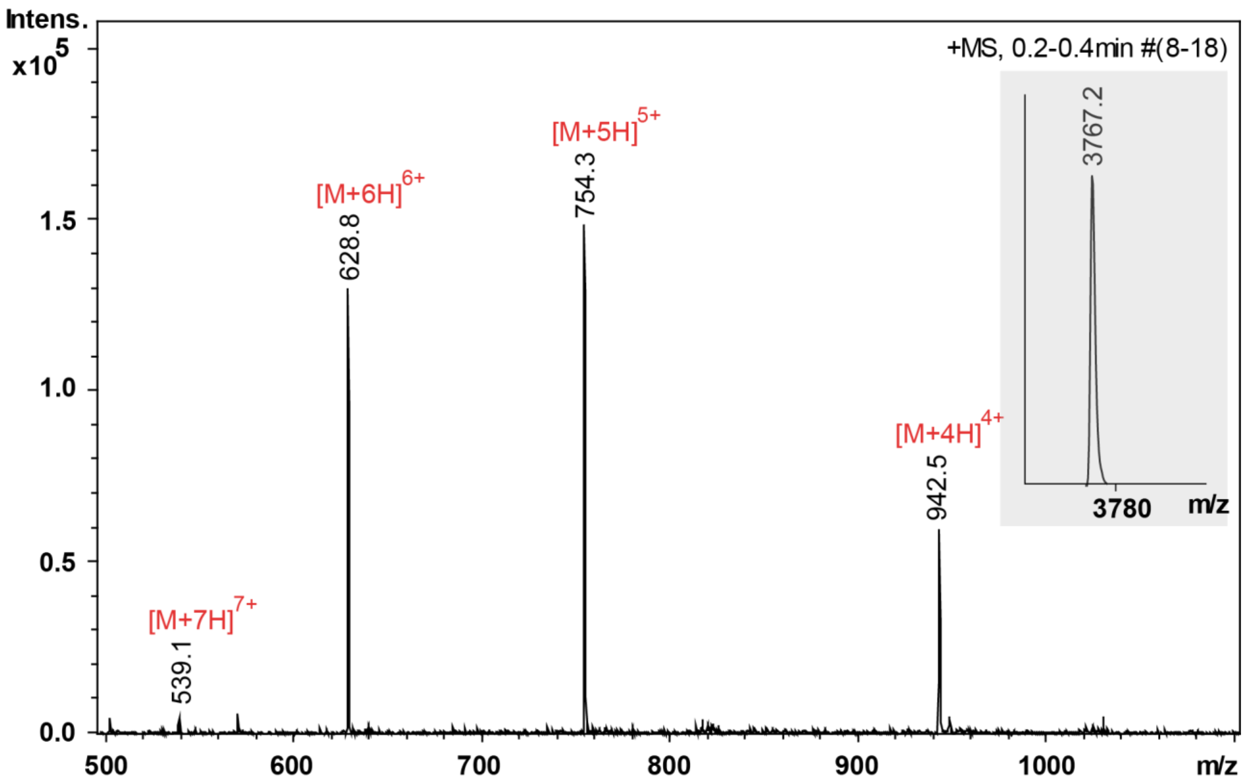

ESI MS analysis of purified peptide **6** with the deconvoluted mass spectrum.

Figure S19.

A.

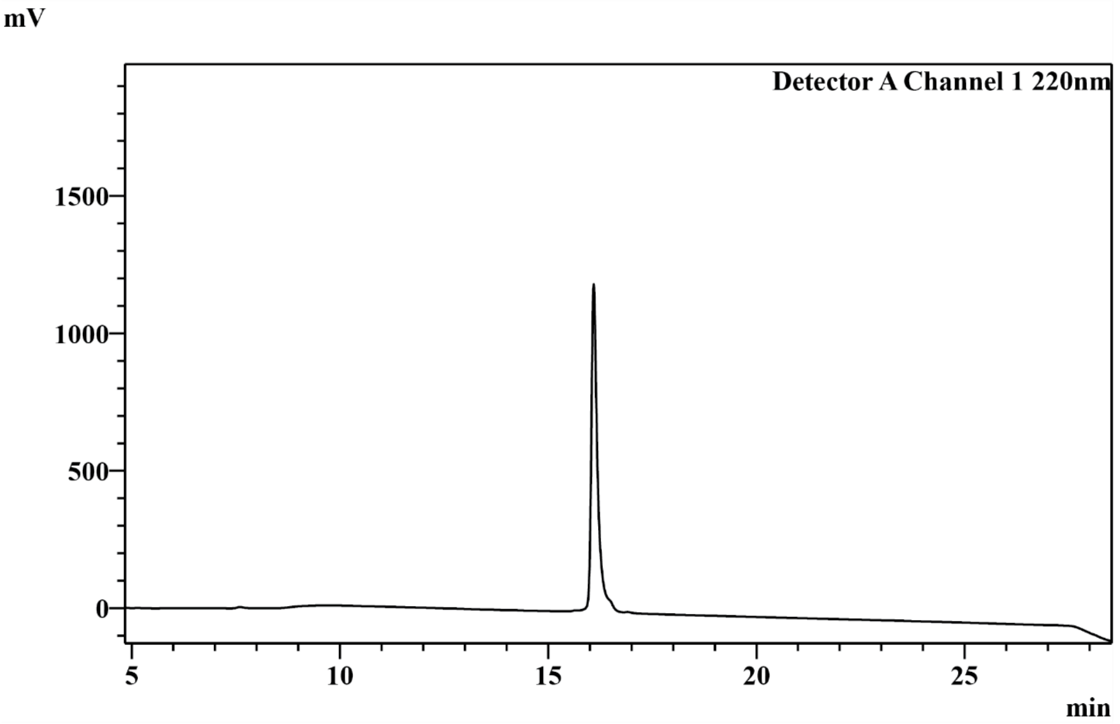

Analytical HPLC chromatogram of purified peptide **6a.**

B.

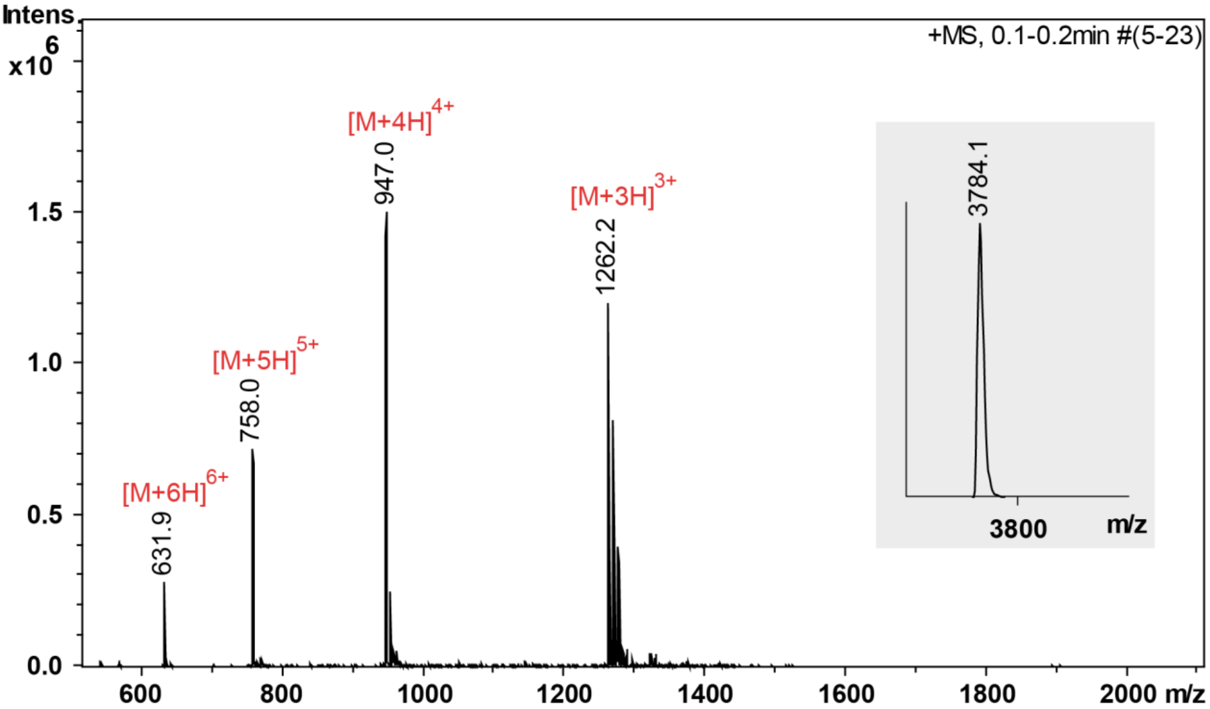

ESI MS analysis of purified peptide **6a** with the deconvoluted mass spectrum.

Figure S20.

A.

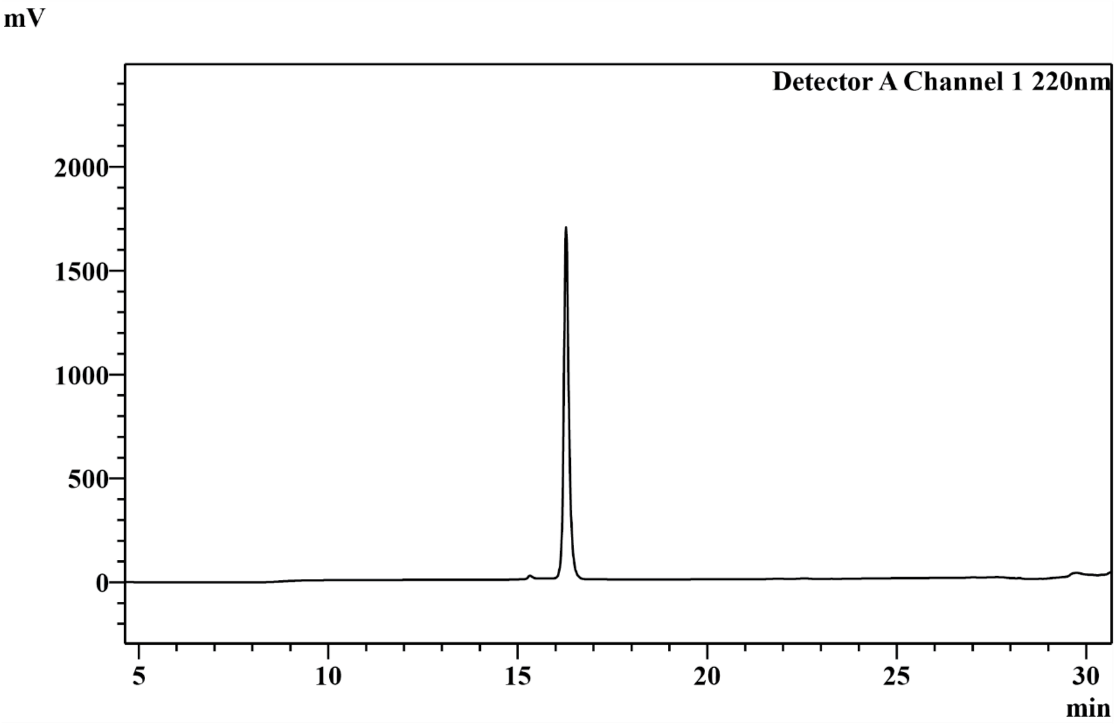

Analytical HPLC chromatogram of purified peptide **7.**

B.

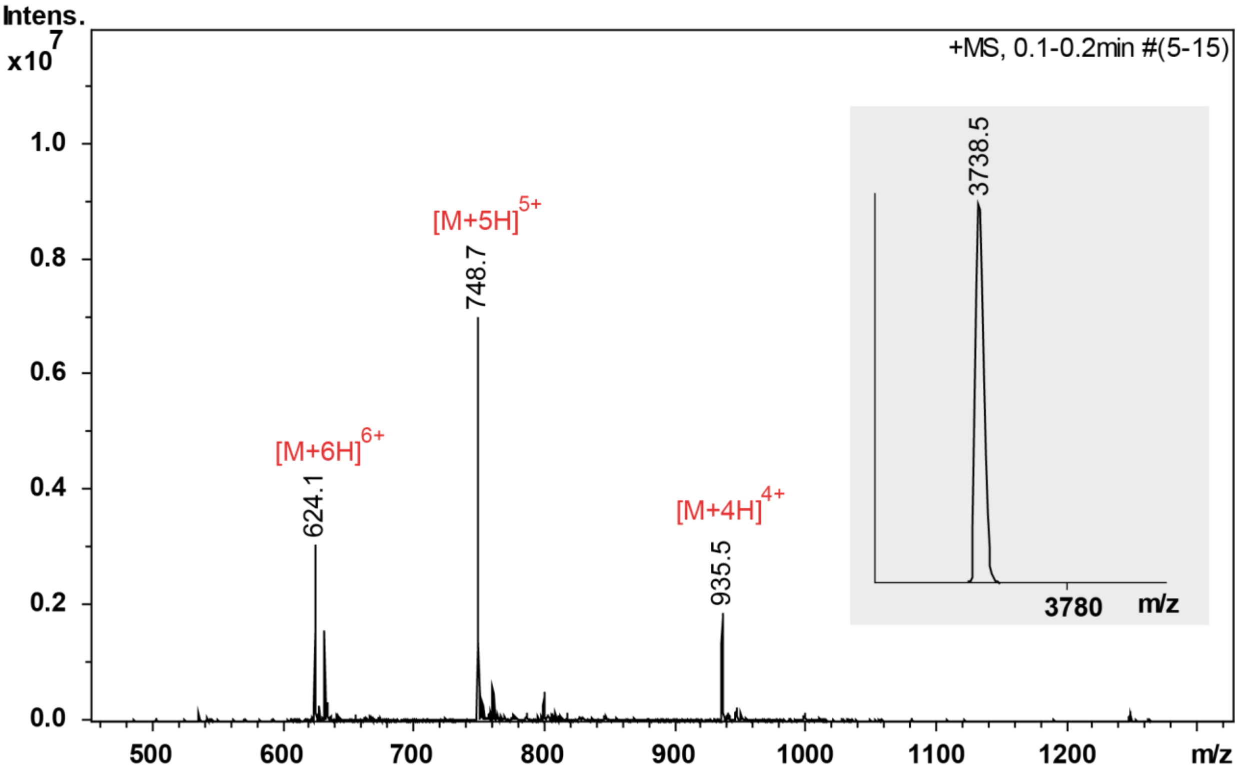

ESI MS analysis of purified peptide **7** with the deconvoluted mass spectrum.

Figure S21.

A.

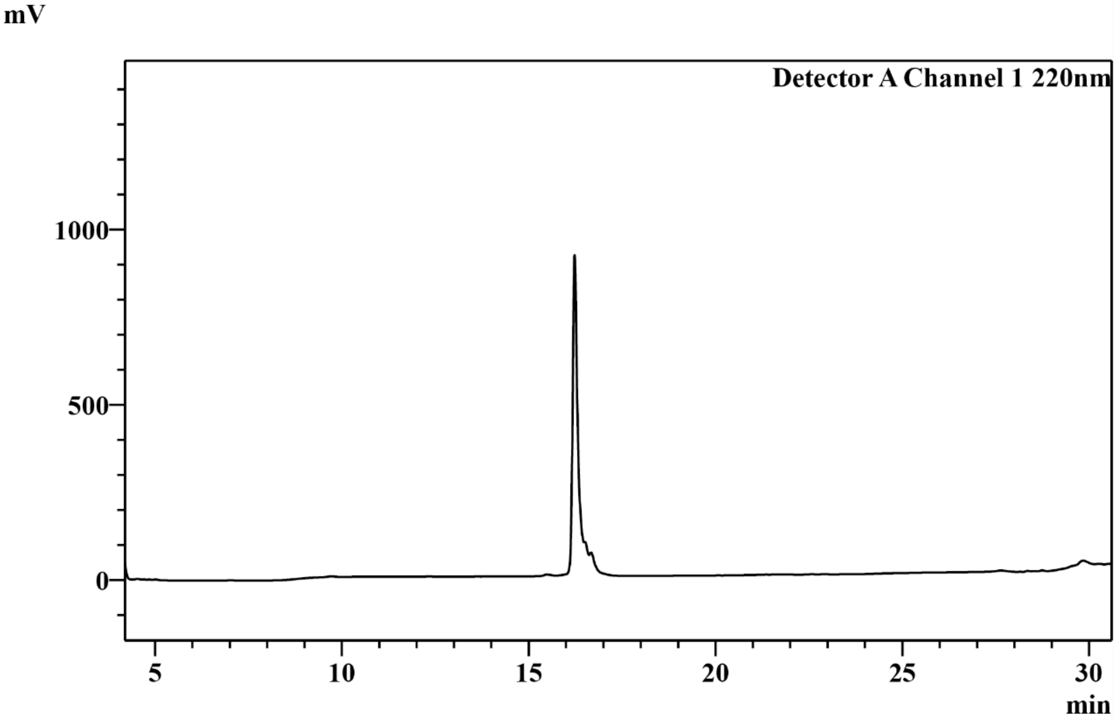

Analytical HPLC chromatogram of purified peptide **7a.**

B.

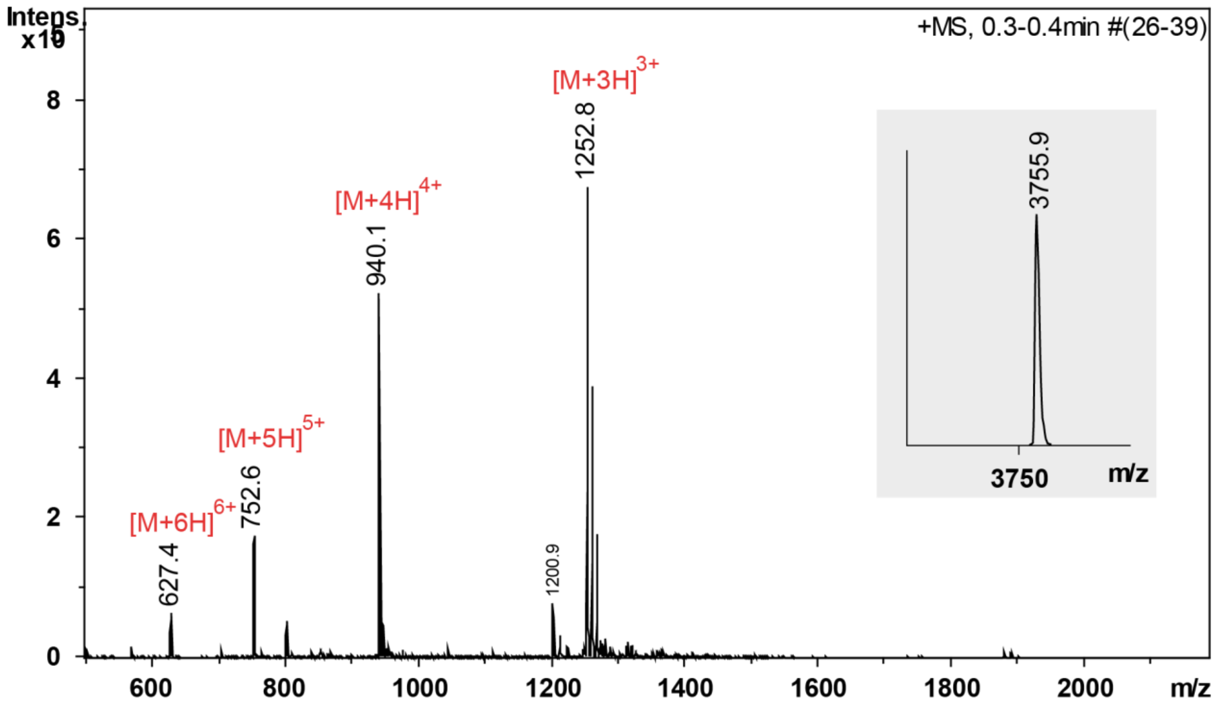

ESI MS analysis of purified peptide **7a** with the deconvoluted mass spectrum.

Figure S22

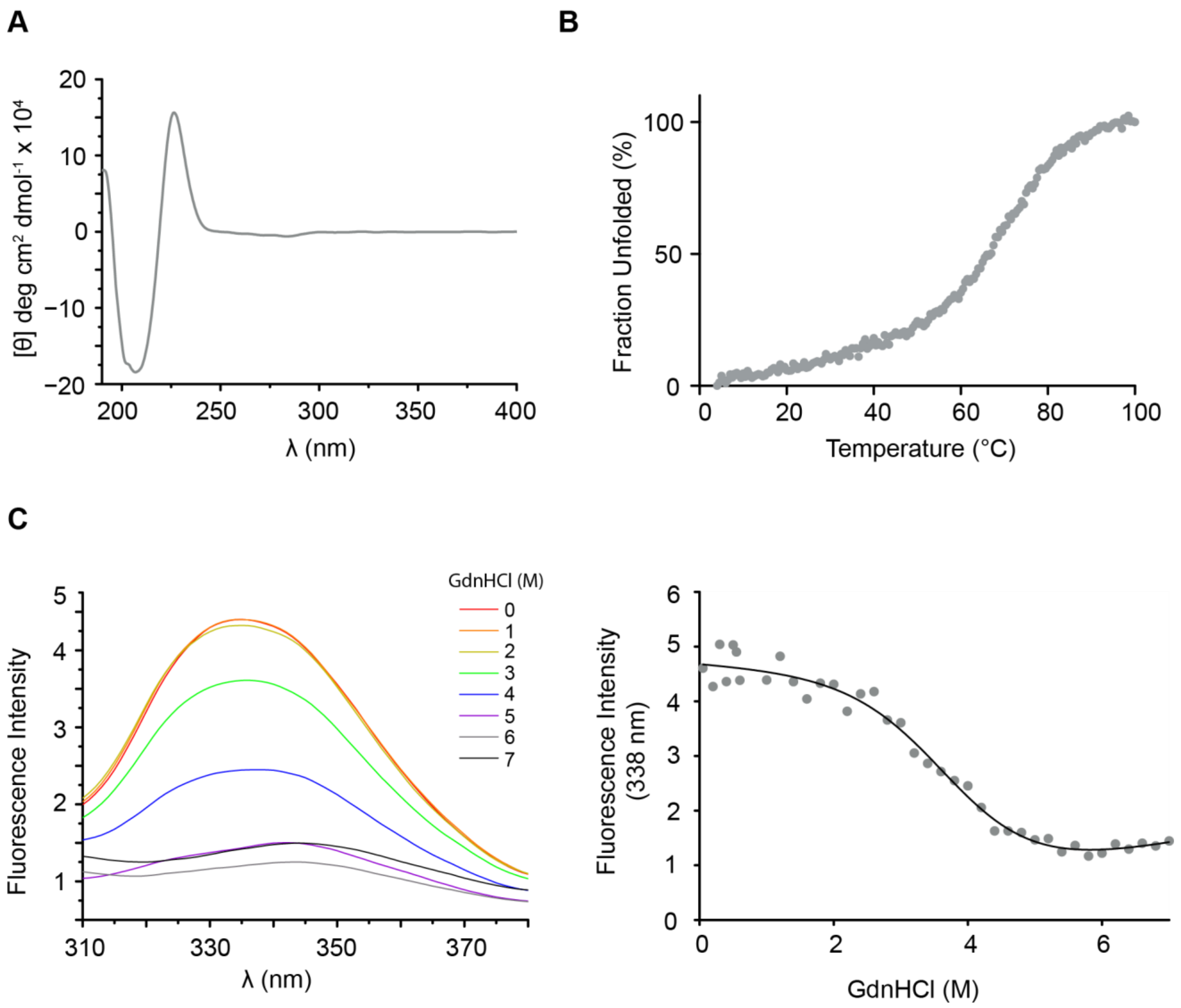

(A) CD scan, (B) temperature-dependent denaturation, and (C) Change in fluorescence intensity with different concentration of GdnHCl and GdnHCl-induced denaturation profile of **6.**

Figure S23.

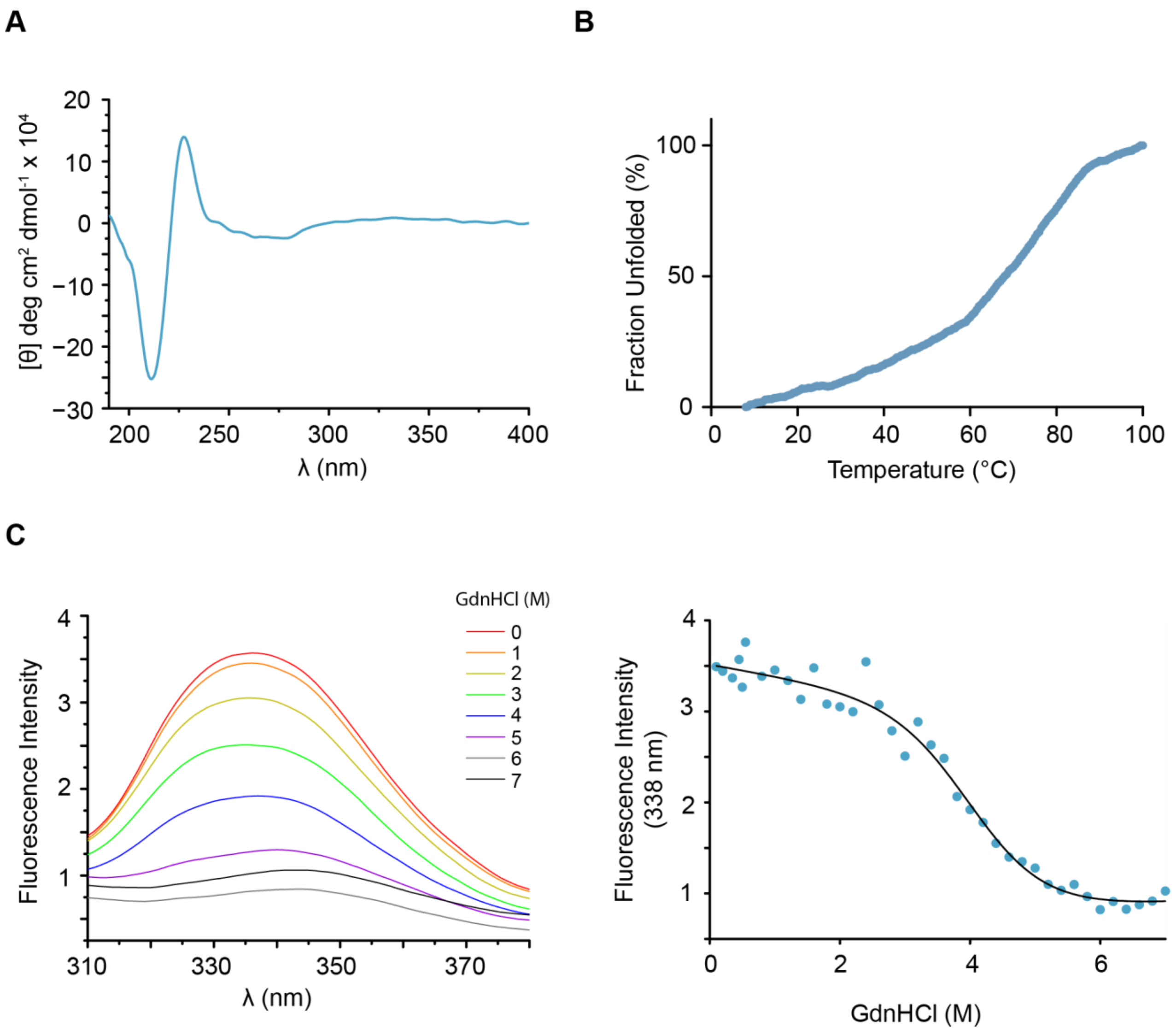

(A) CD scan, (B) temperature-dependent denaturation, and (C) Change in fluorescence intensity with different concentration of GdnHCl and GdnHCl-induced denaturation profile of **6a.**

Figure S24.

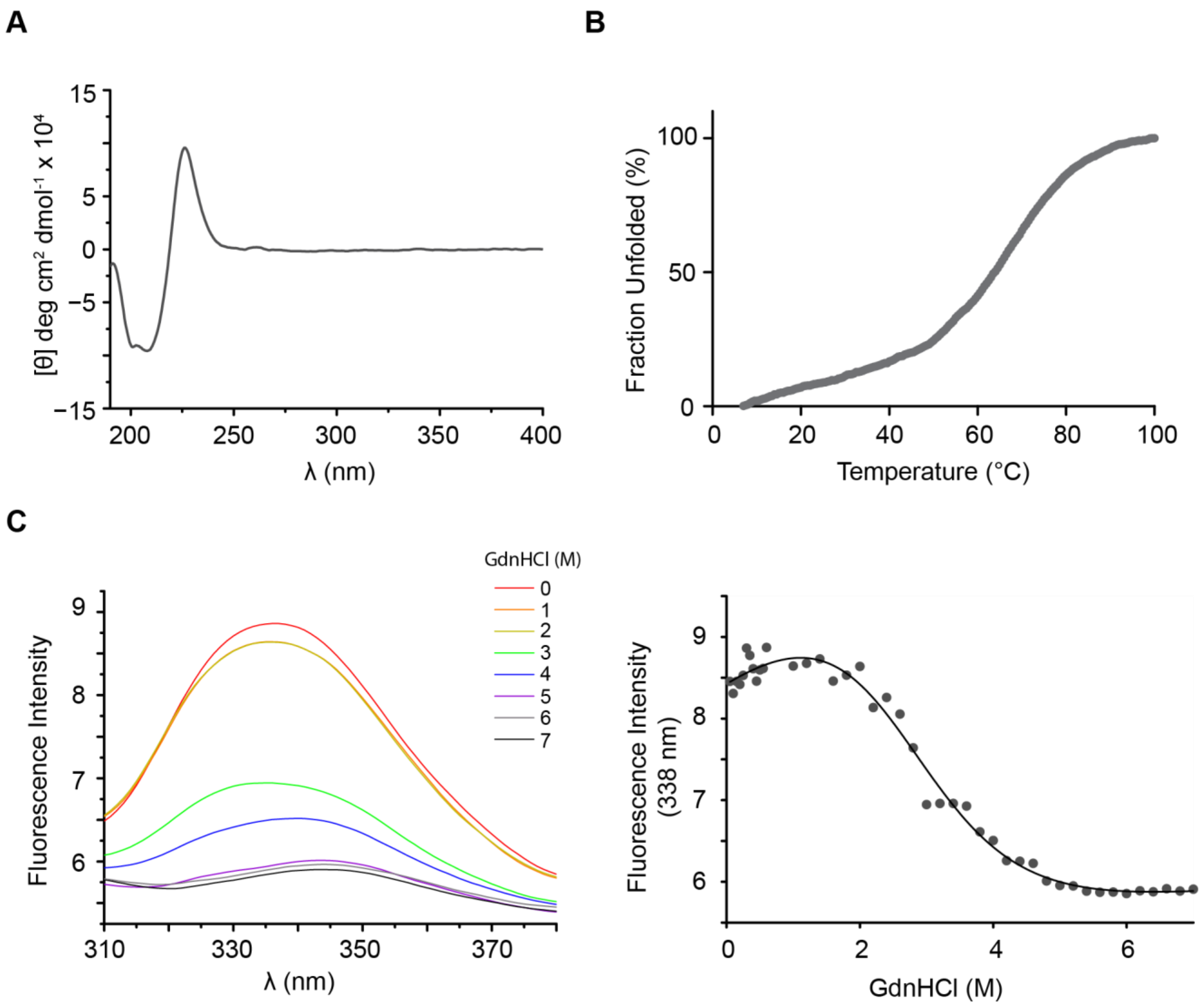

(A) CD scan, (B) temperature-dependent denaturation, and (C) Change in fluorescence intensity with different concentration of GdnHCl and GdnHCl-induced denaturation profile of **7.**

Figure S25.

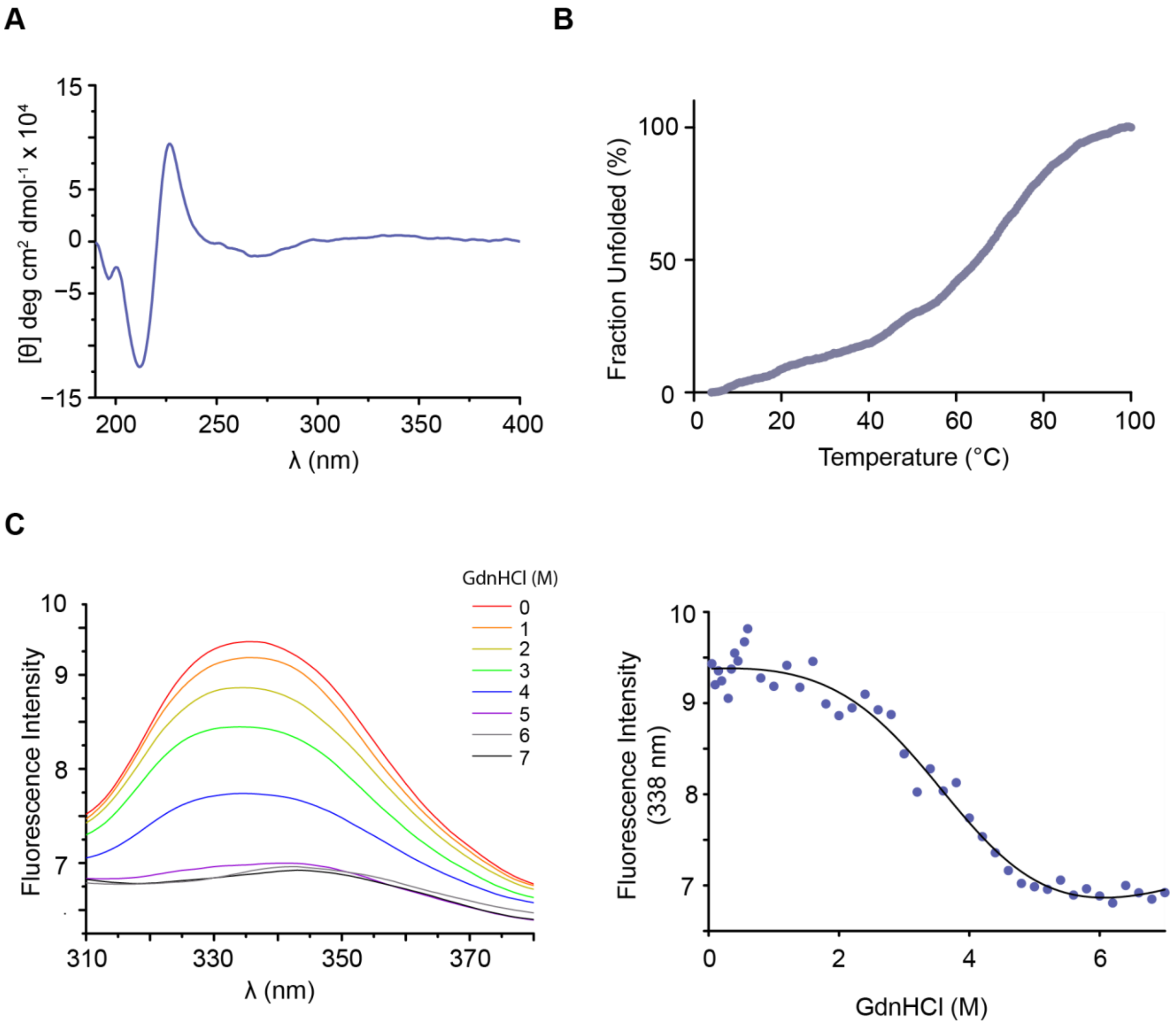

(A) CD scan, (B) temperature-dependent denaturation, and (C) Change in fluorescence intensity with different concentration of GdnHCl and GdnHCl-induced denaturation profile of **7a.**

Figure S26.

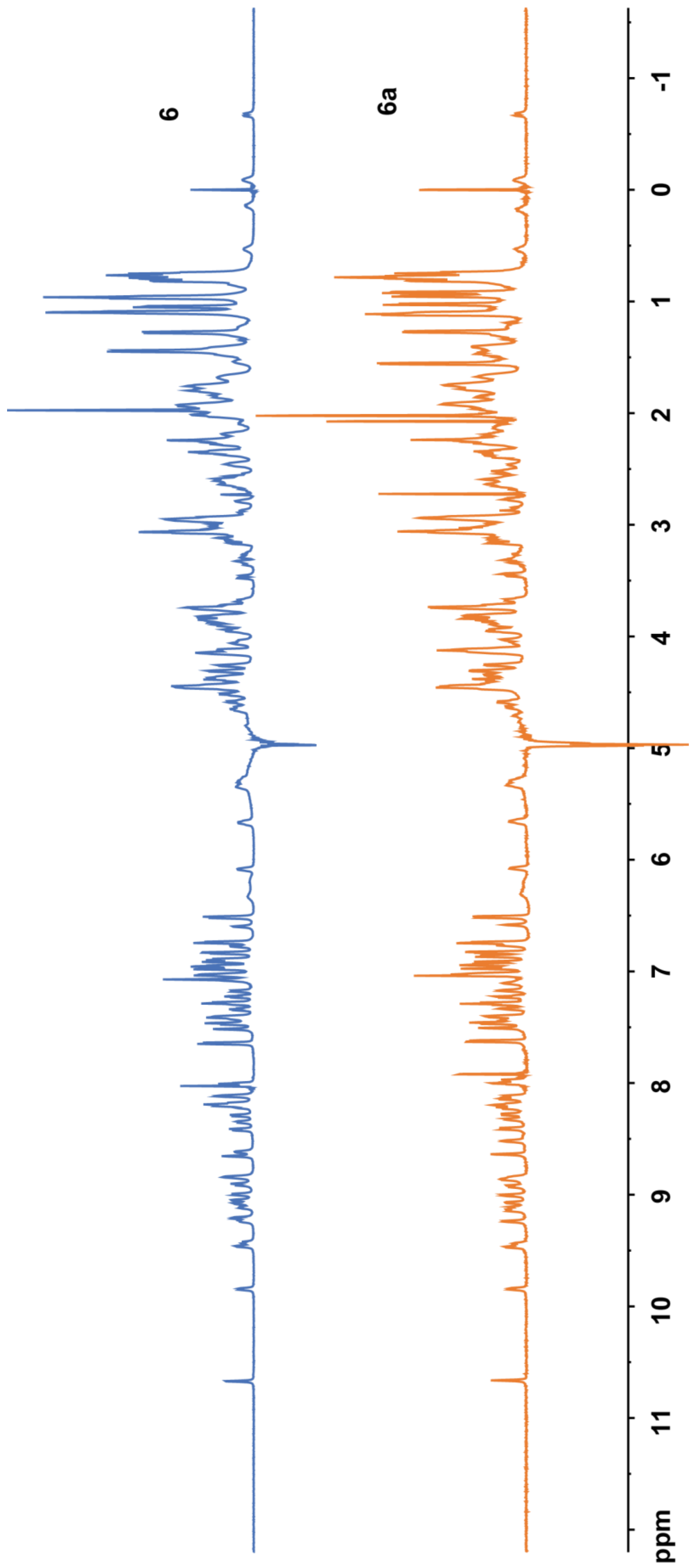

^{1}H NMR overlay of Pin1-WW variants **6** and **6a**.

Figure S27.

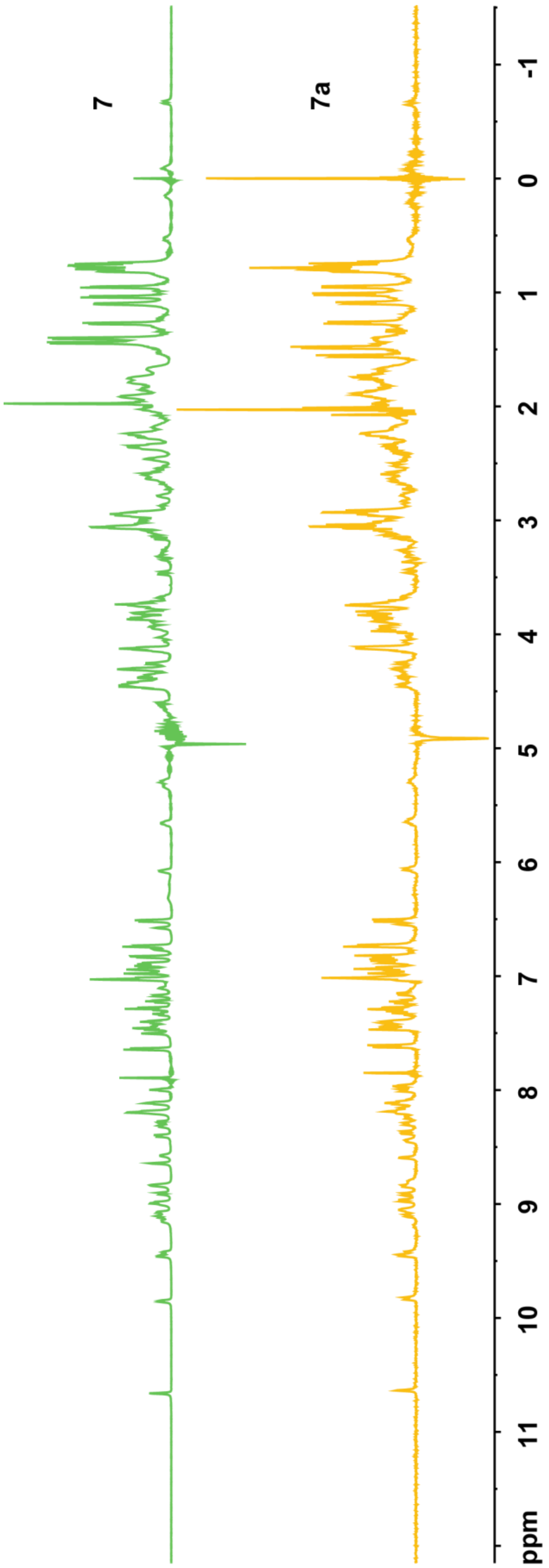

^{1}H NMR overlay of Pin1-WW variants **7** and **7a**.

Table S5. Chemical shifts of Pin1-WW analogs.

	**6**		**6a**		**7**		**7a**	
	H-N	H α	H-N	H α	H-N	H α	H-N	H α
**Lys1**								
**Leu2**		4.64		4.63		4.64		4.63
**Pro3**		4.88		4.89		4.89		4.88
**Pro4**		4.39		4.39		4.4		4.38
**Gly5**	8.84	4.05/3.3	8.84	4.04/3.32	8.83	4.04/3.33	8.81	4.05/3.35
**Trp6**	7.39	5.28	7.39	5.28	7.38	5.28	7.4	5.3
**Glu7**	9.83	4.86	9.83	4.85	9.84	4.85	9.83	4.86
**Lys8**	8.98	4.42	8.99	4.41	8.98	4.41	8.97	4.42
**Arg9**	8.89	4.46	8.91	4.46	8.89	4.46	8.92	4.46
**Met10**	8.18	5.34	8.21	5.33	8.18	5.33	8.2	5.31
**Ser11**	9.22	4.8	9.13	4.75	9.14	4.75	9.06	4.72
**Xaa12**	9.04	3.84	9.23	4.1	8.9	4.45		
**Yaa13**	9.19	4.5	10.65	6.57	8.98	4.96		
**Arg14**	8.18	4.58	7.97	4.58	8.19	4.59	8	4.6
**Val15**	8.6	4.66	8.52	4.7	8.56	4.66	8.45	4.71
**Tyr16**	8.83	4.8	8.86	4.8	8.83	4.8	8.84	4.81
**Tyr17**	9.1	5.3	9.1	5.3	9.1	5.29	9.1	5.31
**Phe18**	9.41	5.65	9.43	5.65	9.41	5.65	9.43	5.65
**Asn19**	8.21	4.44	8.18	4.45	8.18	4.45	8.17	4.47
**His20**	8.18	4.15	8.19	4.14	8.18	4.13	8.19	4.14
**Ile21**	8.34	3.86	8.32	3.86	8.3	3.85	8.3	3.85
**Thr22**	7.41	4.12	7.42	4.11	7.42	4.11	7.43	4.12
**Asn23**	8.1	4.15	8.1	4.15	8.11	4.14	8.11	4.14
**Ala24**	7.16	4.44	7.16	4.44	7.16	4.44	7.16	4.45
**Ser25**	8.4	6.07	8.4	6.07	8.39	6.07	8.38	6.07
**Gln26**	9.45	4.94	9.46	4.94	9.44	4.92	9.45	4.96
**Phe27**	9.06	4.84	9.06	4.83	9.06	4.84	9.05	4.84
**Glu28**	8.1	4.35	8.13	4.33	8.11	4.35	8.12	4.35
**Arg29**	8.64	2.78	8.63	2.77	8.63	2.77	8.59	2.78
**Pro30**		3.9		3.89		3.89		3.9
**Ser31**	8.27	4.3	8.27	4.3	8.27	4.3	8.24	4.32
**Gly32**	7.99	3.83/3.72	7.99	3.83/3.73	7.98	3.83/3.72	7.97	3.84/3.72

Figure S28. Comparison of H$^{\alpha}$ chemical shift deviation of Pin1 WW analogs.

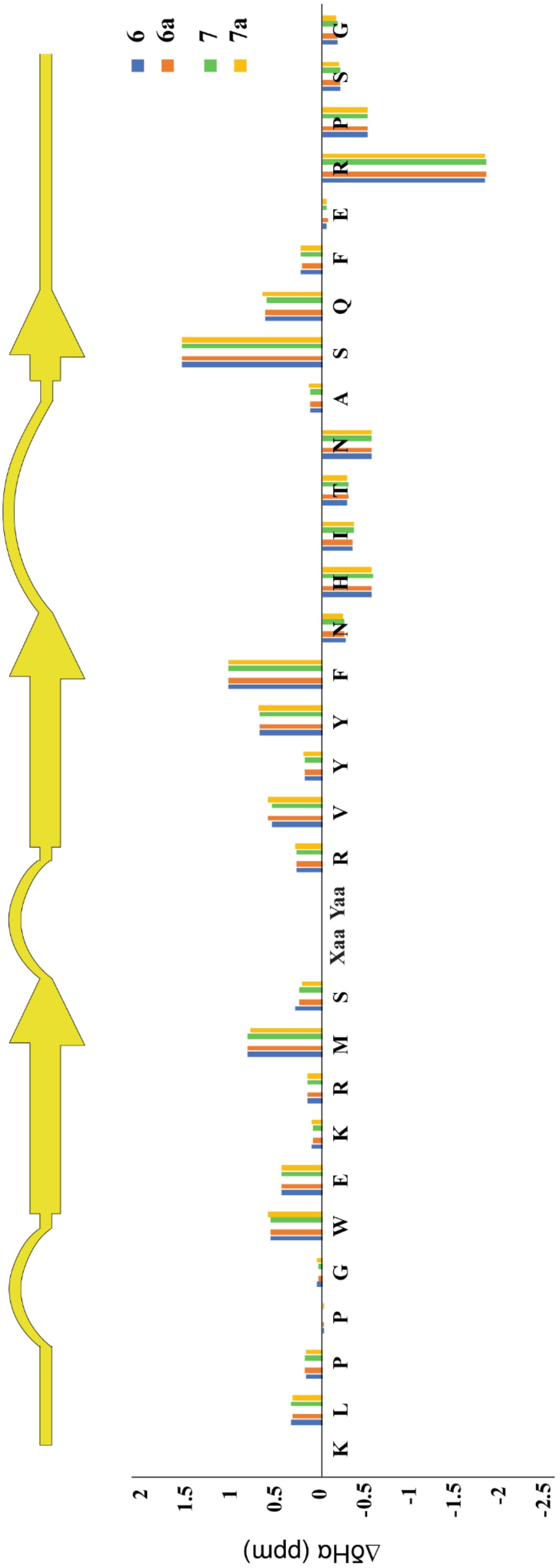

Figure S29.

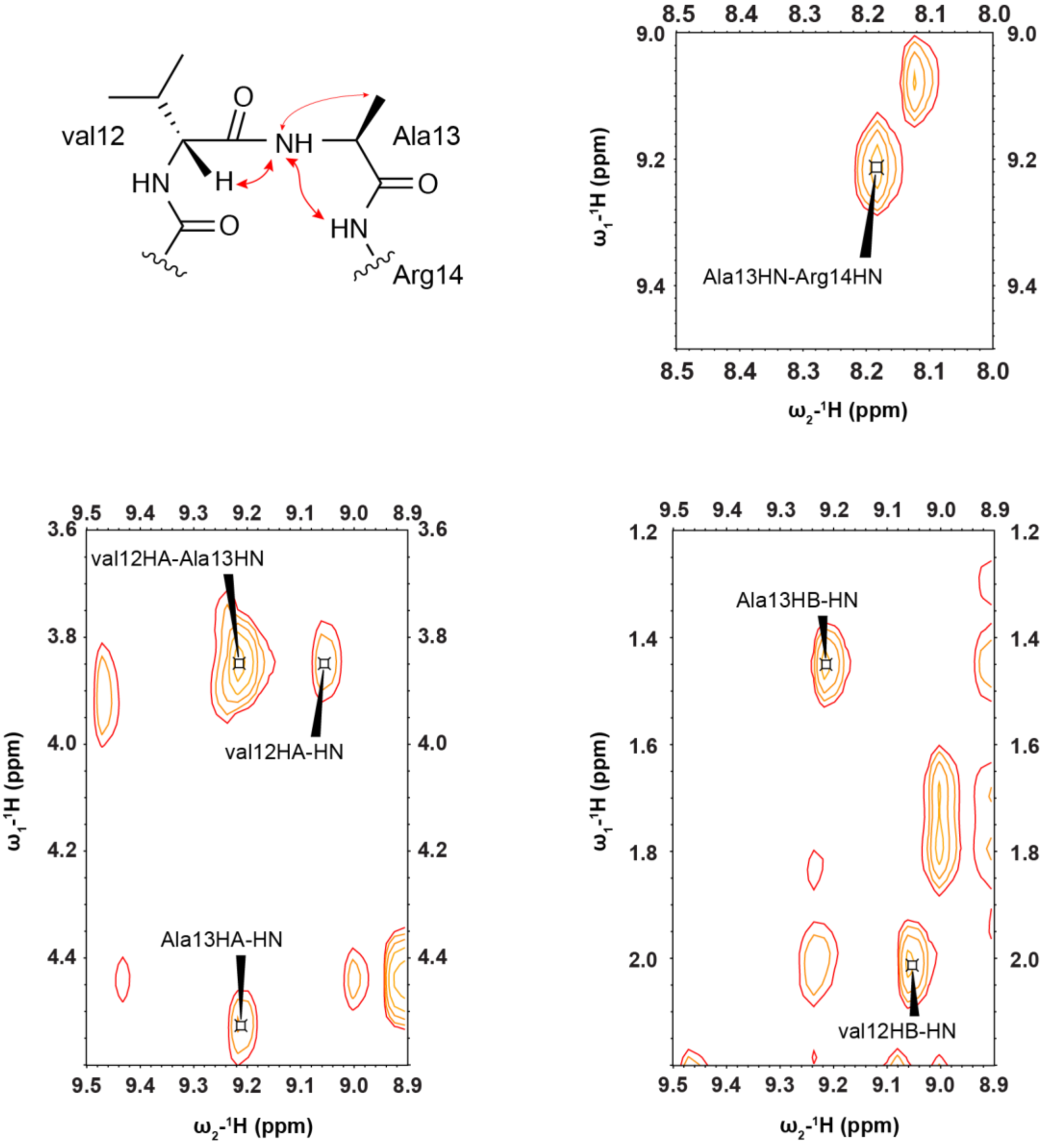

NOEs observed in the loop1 of **6**.

Figure S30.

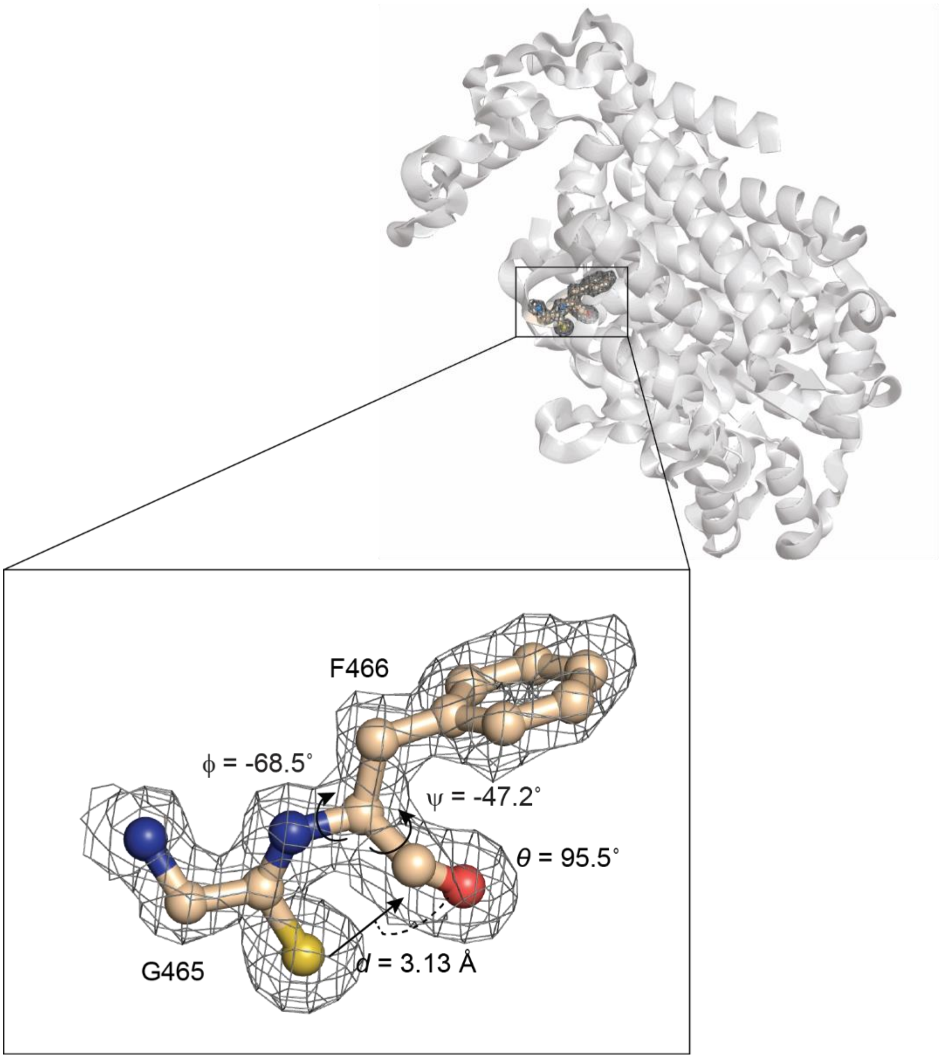

n→π* interaction between thioglycine and phenylalanine in a natural protein Methyl-coenzyme M reductase (PDB code – 1e6y). The electron density map is contoured at 1.0σ.

References:

1. Kaul, R.; Angeles, A. R.; Jager, M.; Powers, E. T.; Kelly, J. W., Incorporating beta-turns and a turn mimetic out of context in loop 1 of the WW domain affords cooperatively folded beta-sheets. *J. Am. Chem. Soc.* **2001,** *123* (22), 5206-12.

2. Greene, R. F., Jr.; Pace, C. N., Urea and guanidine hydrochloride denaturation of ribonuclease, lysozyme, alpha-chymotrypsin, and beta-lactoglobulin. *J. Biol. Chem.* **1974,** *249* (17), 5388-93.

3. Singh, H.; Chauhan, J. S.; Gromiha, M. M.; Open Source Drug Discovery, C.; Raghava, G. P., ccPDB: compilation and creation of data sets from Protein Data Bank. *Nucleic Acids Res.* **2012,** *40* (Database issue), D486-9.

4. Chai, J. D.; Head-Gordon, M., Long-range corrected hybrid density functionals with damped atom-atom dispersion corrections. *Phys. Chem. Chem. Phys.* **2008,** *10* (44), 6615-20.

5. Weinhold, F.; Landis, C. R., *Valency and Bonding: A Natural Bond Orbital Donor–Acceptor Perspective*. Cambridge University Press: 2005.

6. M. J. Frisch, G. W. T., H. B. Schlegel, G. E. Scuseria, M. A. Robb, J. R. Cheeseman, G. Scalmani, V. Barone, B. Mennucci, G. A. Petersson, H. Nakatsuji, M. Caricato, X. Li, H. P. Hratchian, A. F. Izmaylov, J. Bloino, G. Zheng, J. L. Sonnenberg, M. Hada, M. Ehara, K. Toyota, R. Fukuda, J. Hasegawa, M. Ishida, T. Nakajima, Y. Honda, O. Kitao, H. Nakai, T. Vreven, J. A. Montgomery, Jr., J. E. Peralta, F. Ogliaro, M. Bearpark, J. J. Heyd, E. Brothers, K. N. Kudin, V. N. Staroverov, R. Kobayashi, J. Normand, K. Raghavachari, A. Rendell, J. C. Burant, S. S. Iyengar, J. Tomasi, M. Cossi, N. Rega, J. M. Millam, M. Klene, J. E. Knox, J. B. Cross, V. Bakken, C. Adamo, J. Jaramillo, R. Gomperts, R. E. Stratmann, O. Yazyev, A. J. Austin, R. Cammi, C. Pomelli, J. W. Ochterski, R. L. Martin, K. Morokuma, V. G. Zakrzewski, G. A. Voth, P. Salvador, J. J. Dannenberg, S. Dapprich, A. D. Daniels, Ö. Farkas, J. B. Foresman, J. V. Ortiz, J. Cioslowski, and D. J. Fox *Gaussian 09*, Gaussian, Inc., : 2009.

7. Glendening, E. D.; Landis, C. R.; Weinhold, F., NBO 6.0: natural bond orbital analysis program. *J. Comput. Chem.* **2013,** *34* (16), 1429-37.

8. Kabsch, W., Xds. *Acta Crystallogr. D Biol. Crystallogr.* **2010,** *66* (Pt 2), 125-32.

9. Winn, M. D.; Ballard, C. C.; Cowtan, K. D.; Dodson, E. J.; Emsley, P.; Evans, P. R.; Keegan, R. M.; Krissinel, E. B.; Leslie, A. G.; McCoy, A.; McNicholas, S. J.; Murshudov, G. N.; Pannu, N. S.; Potterton, E. A.; Powell, H. R.; Read, R. J.; Vagin, A.; Wilson, K. S., Overview of the CCP4 suite and current developments. *Acta Crystallogr. D Biol. Crystallogr.* **2011,** *67* (Pt 4), 235-42.

10. Emsley, P.; Cowtan, K., Coot: model-building tools for molecular graphics. *Acta Crystallogr. D Biol. Crystallogr.* **2004,** *60* (Pt 12 Pt 1), 2126-32.

11. Adams, P. D.; Afonine, P. V.; Bunkoczi, G.; Chen, V. B.; Davis, I. W.; Echols, N.; Headd, J. J.; Hung, L. W.; Kapral, G. J.; Grosse-Kunstleve, R. W.; McCoy, A. J.; Moriarty, N. W.; Oeffner, R.; Read, R. J.; Richardson, D. C.; Richardson, J. S.; Terwilliger, T. C.; Zwart, P. H., PHENIX: a comprehensive Python-based system for macromolecular structure solution. *Acta Crystallogr. D Biol. Crystallogr.* **2010,** *66* (Pt 2), 213-21.
